# Supplementary material for: Mechanisms of Action of Autophagy Modulators Dissected by Quantitative Systems Pharmacology Analysis
Source: Int J Mol Sci. 2020 Apr 19;21(8):2855. doi: 10.3390/ijms21082855 (PMC7215584; doi:10.3390/ijms21082855)
Supplement: Supplementary file 1 [file ijms-21-02855-s001.pdf]

Supplementary Material

Table of Contents

Figure S1 .... **2**

Figure S2 .... **3**

Figure S3 .... **4**

Table S1. 225 Autophagy Modulators. **5**

Table S2. 993 known targets and 12 predicted targets of autophagy modulators **9**

Table S3. 368 Predicted ATG interactions and experimental validation **33**

Table S4. GO annotation enrichments based on ATG targets **39**

Table S5. Pathways enriched in targets of autophagy modulators, corresponding targets, and autophagy modulators . **42**

Supplementary References **65**

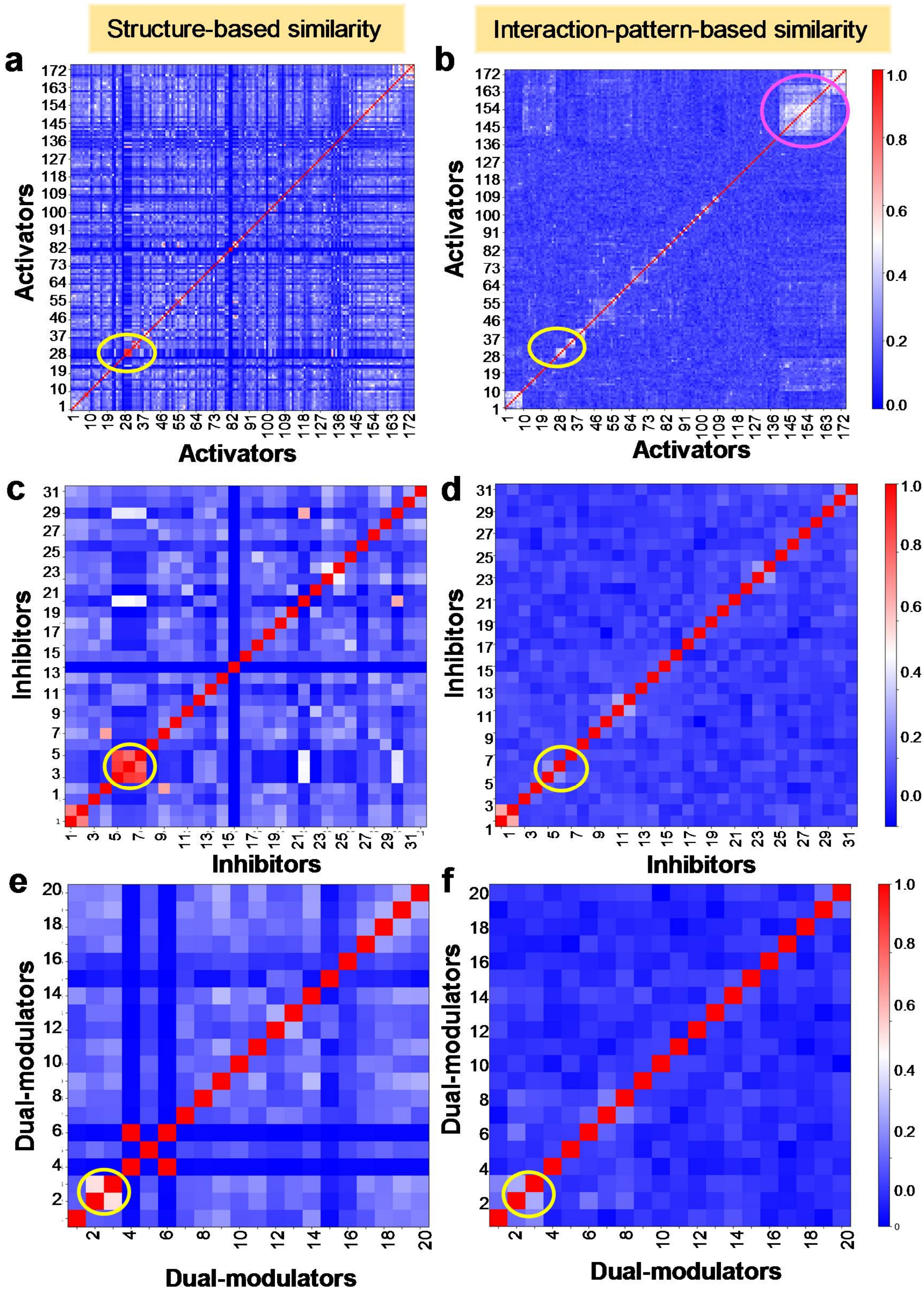

Figure S1. Distribution of the autophagy modulators based on their structure (*left*) and interaction pattern (*right*) similarities. (**a**, **b**) Activators. (**c**, **d**) Inhibitors. (**e**, **f**) Dual-modulators. Structure is characterized by 2D fingerprints, and patterns by the identity of their targets listed in DrugBank.

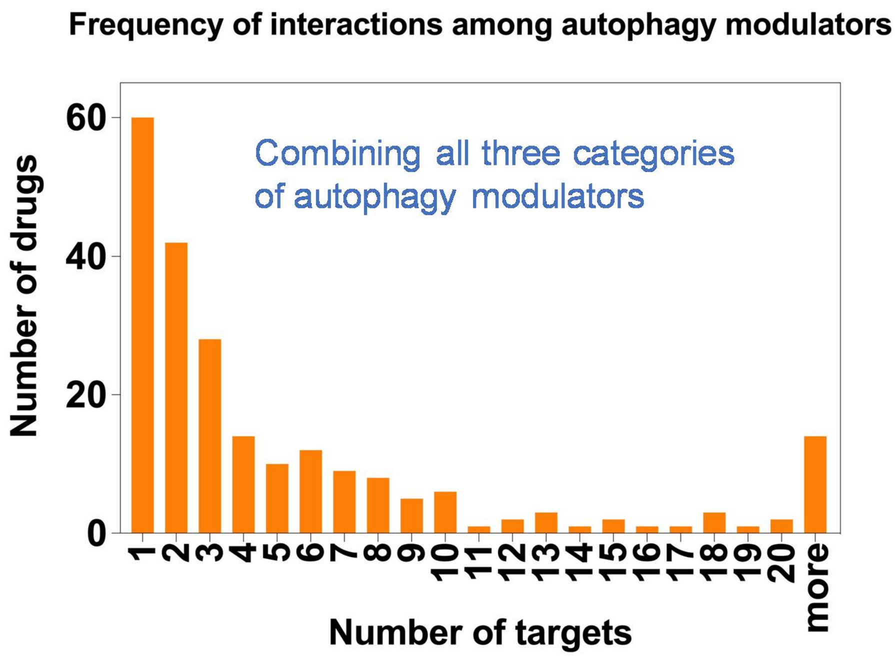

Figure S2. Population of autophagy modulators with different degrees (different numbers of associated targets). Note that ~60 modulators (out of 225) interact with a single protein, while 17 of them interact each with more than 20 targets.

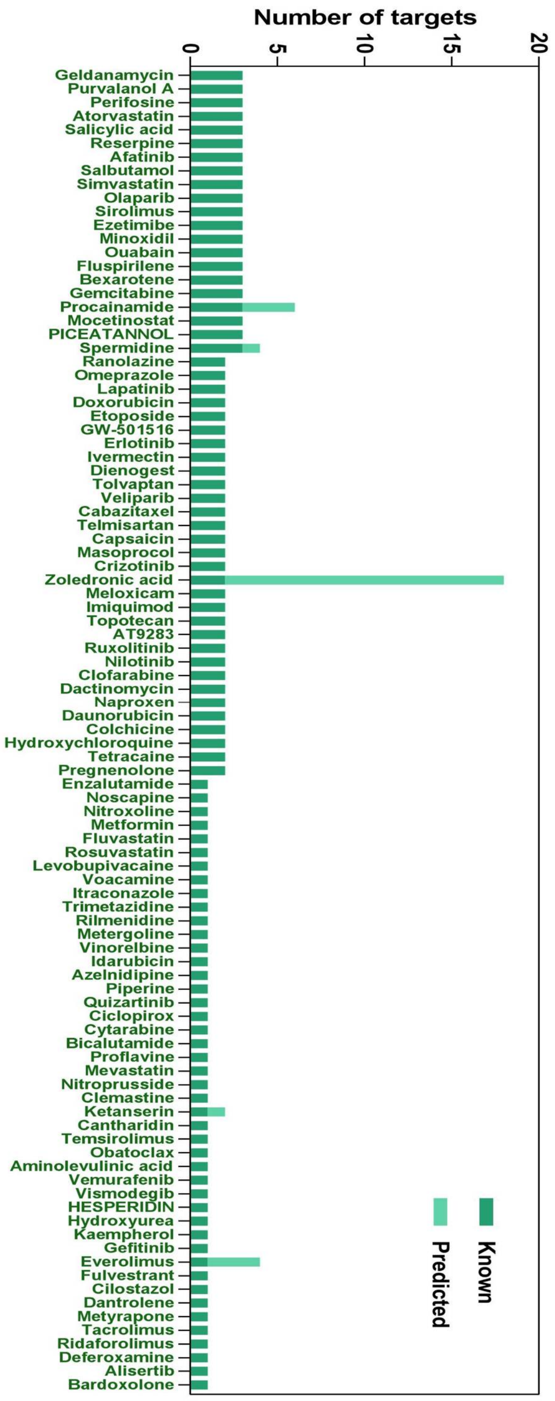

Figure S3. Additional data on the number of known and predicted targets of activators.

Table S1. 225 autophagy modulators.

Activators				
**No.**	**Drug**	**DrugBank ID**	**No. of Known Targets**	**Reference**
1	6-Mercaptopurine	DB01033	4	1
2	Acetazolamide	DB00819	8	2
3	Actinomycin D	DB00970	2	3
4	Afatinib	DB08916	3	4
5	Alisertib	DB05220	1	5
6	Amiodarone	DB01118	7	6
7	Aspirin	DB00945	18	7
8	AT9283	DB05169	2	8
9	Bexarotene	DB00307	3	9
10	Bicalutamide	DB01128	1	10
11	Cabazitaxel	DB06772	2	11
12	Cabergoline	DB00248	20	12
13	Capsaicin	DB06774	2	13
14	Carbamazepine	DB00564	4	14
15	Carfilzomib	DB08889	6	15
16	Ciclopirox	DB01188	1	16
17	Cilostazol	DB01166	1	17
18	Clofarabine	DB00631	2	18
19	Colchicine	DB01394	2	19
20	Crizotinib	DB08865	2	20
21	Curcumin	DB11672	5	21
22	Cytarabine	DB00987	1	22
23	Dasatinib	DB01254	23	23
24	Dexamethasone	DB01234	5	24
25	Diazoxide	DB01119	6	25
26	Dienogest	DB09123	2	26
27	Enzalutamide	DB08899	1	27
28	Erlotinib	DB00530	2	28
29	Etoposide	DB00773	2	29
30	Ezetimibe	DB00973	3	30
31	Fasudil	DB08162	4	31
32	Fenofibrate	DB01039	5	32
33	Flavopiridol	DB03496	12	33
34	Fluoxetine	DB00472	7	34
35	Fulvestrant	DB00947	1	35
36	Gefitinib	DB00317	1	36
37	Gemcitabine	DB00441	3	37
38	Genistein	DB01645	13	38
39	Glibenclamide	DB01016	9	39
40	GW 501516	DB05416	2	40
41	Hesperidin	DB04703	1	41
42	Hydroxyurea	DB01005	1	42
43	Imatinib	DB00619	9	43
44	Imiquimod	DB00724	2	44
45	Isoniazid	DB00951	4	45
46	Itraconazole	DB01167	1	46
47	Ivermectin	DB00602	2	47
48	Ketanserin	DB12465	1	48
49	Lamotrigine	DB00555	35	49
50	Lapatinib	DB01259	2	50
51	Meloxicam	DB00814	2	51
52	Metyrapone	DB01011	1	52
53	Mevastatin	DB06693	1	53
54	Mifepristone	DB00834	4	54
55	Mocetinostat	DB11830	3	55
56	Niacin	DB00627	4	56
57	Nifedipine	DB01115	9	57
58	Nilotinib	DB04868	2	58
59	Nitroxoline	DB01422	1	59

60	Nordihydroguaiaretic acid	DB00179	2	60
61	Noscapine	DB06174	1	61
62	Obatoclax	DB12191	1	62
63	Olanzapine	DB00334	48	63
64	Olaparib	DB09074	3	64
65	Panobinostat	DB06603	11	65
66	Pazopanib	DB06589	10	66
67	Pemetrexed	DB00642	4	67
68	Pentoxifylline	DB00806	6	68
69	Perifosine	DB06641	3	69
70	Piceatannol	DB08399	3	70
71	Piperine	DB12582	1	71
72	Ponatinib	DB08901	15	72
73	Pregnenolone	DB02789	2	73
74	Purvalanol A	DB04751	3	74
75	Quercetin	DB04216	27	75
76	Quizartinib	DB12874	1	76
77	Regorafenib	DB08896	18	77
78	Resveratrol	DB02709	25	78
79	Ruxolitinib	DB08877	2	79,80
80	Salicylic acid	DB00936	3	81
81	Sertindole	DB06144	8	82
82	Simvastatin	DB00641	3	83
83	Sorafenib	DB00398	10	84
84	Spironolactone	DB00421	27	85
85	Sulfasalazine	DB00795	10	86
86	Sulindac	DB00605	7	87
87	Sunitinib	DB01268	8	88
88	Tacrolimus	DB00864	1	89
89	Tamoxifen	DB00675	16	90
90	Taurine	DB01956	21	91
91	Telmisartan	DB00966	2	92
92	Tolvaptan	DB06212	2	93
93	Topotecan	DB01030	2	94
94	Troglitazone	DB00197	9	95
95	Valproic acid	DB00313	23	96
96	Vemurafenib	DB08881	1	97
97	Vinorelbine	DB00361	1	98
98	Vismodegib	DB08828	1	99
99	Kaempferol	DB01852	1	100
100	L-Nicotine	DB00184	13	101
101	4-Hydroxytamoxifen	DB04468	7	102
102	Acetylcholine	DB03128	6	103
103	Atorvastatin	DB01076	3	104
104	Aminolevulinic acid	DB00855	1	105
105	Bardoxolone	DB12651	1	106
106	Chlorpromazine	DB00477	26	107
107	Citalopram	DB00215	5	108
108	Clemastine	DB00283	1	109
109	Deferoxamine	DB00746	1	110
110	Reserpine	DB00206	3	111
111	Doxazosin	DB00590	6	112
112	Dronedarone	DB04855	18	113
113	Fluvastatin	DB01095	1	114
114	Idarubicin	DB01177	1	115
115	Levobupivacaine	DB01002	1	116
116	Loperamide	DB00836	7	117
117	Maprotiline	DB00934	17	118
118	Memantine	DB01043	7	119
119	Metformin	DB00331	1	120
120	Naproxen	DB00788	2	121
121	Nicardipine	DB00622	15	122

122	Nitroprusside	DB00325	1	123
123	Nortriptyline	DB00540	24	124
124	Daunorubicin	DB00694	2	125
125	Doxorubicin	DB00997	2	126
126	Glucosamine	DB01296	4	127
127	Ouabain	DB01092	3	128
128	Paroxetine	DB00715	8	129
129	Prazosin	DB00457	8	130
130	Proflavine	DB01123	1	131
131	Ranolazine	DB00243	2	132
132	Rosuvastatin	DB01098	1	133
133	Triflupromazine	DB00508	5	134
134	Trimetazidine	DB09069	1	135
135	Veliparib	DB07232	2	136
136	Zoledronic acid	DB00399	2	137
137	Procainamide	DB01035	3	138
138	Raloxifene	DB00481	4	139
139	Salbutamol	DB01001	3	140
140	Vinblastine	DB00570	6	141
141	Dantrolene	DB01219	1	142
142	Theophylline	DB00277	14	143, 144
143	Clonidine	DB00575	6	145
144	Tetracaine	DB09085	2	146
145	Hydroxychloroquine	DB01611	2	147
146	Flunarizine	DB04841	5	148
147	XL765 (Voxtalisib)	DB05241	5	149
148	Felodipine	DB01023	13	150
149	Isradipine	DB00270	7	151
150	Geldanamycin	DB02424	3	152
151	Thalidomide	DB01041	7	153
152	Zinc	DB01593	124	154
153	Copper	DB09130	145	155
154	Voacamine	DB04877	1	156
155	Artenimol	DB11638	78	157
156	Rapamycin	DB00877	3	158
157	Everolimus	DB01590	1	158
158	Temsirolimus	DB06287	1	159
159	Spermidine	DB03566	3	160
160	Verapamil	DB00661	19	160
161	Ridaforolimus	DB06233	1	161
162	Fluspirilene	DB04842	3	158
163	Minoxidil	DB00350	3	158
164	Rilmenidine	DB11738	1	158
165	Metergoline	DB13520	1	158
166	Fluphenazine	DB00623	6	162
167	Cantharidin	DB12328	1	163
168	Pimozide	DB01100	4	158
169	Levosimendan	DB00922	4	164
170	Omeprazole	DB00338	2	165
171	Azelnidipine	DB09230	1	166
172	Lacidipine	DB09236	8	166
173	Nilvadipine	DB06712	6	166
174	Nitrendipine	DB01054	8	158
**Inhibitors**				
**No.**	**Drug**	**DrugBank ID**	**No. of Known Targets**	**Reference**
1	Azithromycin	DB00207	1	167
2	Heparin	DB01109	12	168
3	Isosorbide mononitrate	DB01020	1	169
4	PX-478	DB06082	1	170
5	Mefloquine	DB00358	2	171
6	Pantoprazole	DB00213	1	172
7	Chloroquine	DB00608	3	173
8	Atropine	DB00572	8	103

9	Propranolol	DB00571	5	174
10	Quinacrine	DB01103	3	175
11	Esmolol	DB00187	1	176
12	Clarithromycin	DB01211	3	177
13	Clotrimazole	DB00257	3	178
14	Ebselen	DB12610	1	179
15	Erythromycin	DB00199	3	180
16	LY294002	DB02656	24	181
17	Methylprednisolone	DB00959	1	182
18	Rhein	DB13174	3	183
19	Rupatadine	DB11614	2	184
20	Enalaprilat	DB09477	2	185
21	Rutin	DB01698	2	186,187
22	Sildenafil	DB00203	3	188
23	Wortmannin	DB08059	4	189
24	Estradiol	DB00783	10	190
25	Nocodazole	DB08313	1	160
26	Bafilomycin A1	DB06733	1	160
27	Concanamycin A	DB14062	1	158
28	Indomethacin	DB00762	2	191
29	Fostamatinib	DB12010	303	192
30	SR-9011	DB14014	2	193
31	SR-9009	DB14013	2	193
**Dual-modulators**				
**No.**	**Drug**	**DrugBank ID**	**No. of Known Targets**	**Reference**
1	Dexmedetomidine	DB00633	1	194,195
2	Emodin	DB07715	2	196,197
3	Sitagliptin	DB01261	1	198,199
4	2-Methoxyestradiol	DB02342	5	200,201
5	Ginsenoside Rb	DB06749	1	202,203
6	Rosiglitazone	DB00412	7	204,205
7	Berberine	DB04115	1	206
8	Irinotecan	DB00328	9	207,208
9	Esomeprazole	DB00736	1	209
10	Thioridazine	DB00679	6	210
11	Adenosine	DB00640	4	211
12	Bortezomib	DB00188	2	212,213
13	Paclitaxel	DB01229	6	214,215
14	Vincristine	DB00541	2	216,217
15	Calcium	DB01373	20	166,218
16	Lithium	DB01356	4	219,220
17	Melatonin	DB01065	10	159
18	Nimodipine	DB00393	10	221,222
19	Trifluoperazine	DB00831	6	223,224
20	Vorinostat	DB02546	5	225,226

Table S2. 993 known targets and 12 predicted targets of autophagy modulators. Rows highlighted in *yellow* (6 of them) are the targets that interact with all three categories of modulators; those highlighted in *green* are the 32 ATG proteins participating in autophagy pathways (KEGG id: hsa04140)

#	Gene Name	Protein Name	No. Drugs	Activator	Inhibitor	Dual-modulator
**Known Targets**						
1	NR1I2	Nuclear receptor subfamily 1 group I member 2	17	Dexamethasone, Piperine, Fenofibrate, Tamoxifen, Genistein, Spironolactone, Resveratrol, Mifepristone, Nifedipine, Pregnenolone, Erlotinib, Carbamazepine, Quercetin, Afimoxifene	Estradiol, Clotrimazole	Paclitaxel
2	CACNB2	Voltage-dependent L-type calcium channel subunit beta-2	11	Spironolactone, Lacidipine, Felodipine, Isradipine, Nilvadipine, Nicardipine, Nifedipine, Nitrendipine, Dronedarone, Verapamil	-	Nimodipine
3	CACNA1D	Voltage-dependent L-type calcium channel subunit alpha-1D	11	Spironolactone, Lacidipine, Felodipine, Isradipine, Nilvadipine, Nicardipine, Nifedipine, Nitrendipine, Dronedarone, Verapamil	-	Nimodipine
4	KCNH2	Potassium voltage-gated channel subfamily H member 2	14	Sertindole, Pimozide, Tamoxifen, Chlorpromazine, Prazosin, Fluoxetine, Amiodarone, Procainamide, Dronedarone, Verapamil, Doxazosin	Clarithromycin, Erythromycin	Thioridazine
5	CALM1	Calmodulin	11	Loperamide, Felodipine, Flunarizine, Fluphenazine, Nicardipine, Chlorpromazine, Nifedipine, Pimozide	-	Melatonin, Calcium, Trifluoperazine
6	KIT	Mast/stem cell growth factor receptor Kit	9	Nilotinib, Sunitinib, Regorafenib, Pazopanib, Sorafenib, Ponatinib, Dasatinib, Imatinib	Fostamatinib	-
7	CACNA1C	Voltage-dependent L-type calcium channel subunit alpha-1C	12	Spironolactone, Lacidipine, Felodipine, Isradipine, Nilvadipine, Nicardipine, Nifedipine, Nitrendipine, Dronedarone, Verapamil	-	Nimodipine, Calcium
8	CACNA1S	Voltage-dependent L-type calcium channel subunit alpha-1S	10	Spironolactone, Lacidipine, Felodipine, Isradipine, Nilvadipine, Nifedipine, Nitrendipine, Dronedarone, Verapamil	-	Nimodipine
9	PDGFRB	Platelet-derived growth factor receptor beta	7	Sunitinib, Regorafenib, Pazopanib, Sorafenib, Dasatinib, Imatinib	Fostamatinib	-
10	MTOR	Serine/threonine-protein kinase mTOR	6	XL765, Everolimus, Temsirolimus, Ridaforolimus, Sirolimus	Fostamatinib	-
11	CACNA2D1	Voltage-dependent calcium channel subunit alpha-2/delta-1	7	Spironolactone, Nilvadipine, Felodipine, Isradipine, Nicardipine, Nifedipine, Nitrendipine	-	-
12	PDGFRA	Platelet-derived growth factor receptor alpha	6	Ponatinib, Pazopanib, Regorafenib, Sunitinib, Imatinib	Fostamatinib	-
13	CACNA1H	Voltage-dependent T-type calcium channel subunit alpha-1H	7	Spironolactone, Felodipine, Flunarizine, Isradipine, Amiodarone, Nifedipine, Nitrendipine	-	-
14	AHR	Aryl hydrocarbon receptor	7	Atorvastatin, Cantharidin, Quercetin, Resveratrol, Omeprazole	-	Nimodipine, Emodin
15	ADRA1A	Alpha-1A adrenergic receptor	16	Sertindole, Doxazosin, Nicardipine, Lamotrigine, Chlorpromazine, Prazosin, Clonidine, Citalopram, Cabergoline, Olanzapine, Dronedarone, Verapamil, Nortriptyline, Maprotiline	-	Thioridazine, Trifluoperazine
16	CACNA2D2	Voltage-dependent calcium channel subunit alpha-2/delta-2	5	Spironolactone, Felodipine, Isradipine, Amiodarone, Nitrendipine	-	-
17	TUBB	Tubulin beta chain	6	Colchicine, Vinorelbine, Copper, Vinblastine, Artenimol	-	Vincristine
18	SHBG	Sex hormone-binding globulin	7	Spironolactone, Tamoxifen, Masoprocol, Zinc, Genistein, Quercetin, Afimoxifene	-	-
19	CBR1	Carbonyl reductase [NADPH] 1	4	Quercetin, Resveratrol, Curcumin	Rutin	-
20	ADRA1B	Alpha-1B adrenergic receptor	13	Sertindole, Doxazosin, Nicardipine, Chlorpromazine, Prazosin, Clonidine, Cabergoline, Olanzapine, Dronedarone, Verapamil, Nortriptyline, Maprotiline	-	Thioridazine
21	ABL1	Tyrosine-protein kinase ABL1	6	Ponatinib, Nilotinib, Regorafenib, Dasatinib, Imatinib	Fostamatinib	-
22	CACNB1	Voltage-dependent L-type calcium channel subunit beta-1	6	Spironolactone, Lacidipine, Dronedarone, Azelnidipine, Verapamil	-	Nimodipine
23	FRK	Tyrosine-protein kinase FRK	3	Regorafenib, Dasatinib	Fostamatinib	-
24	HDAC2	Histone deacetylase 2	5	Theophylline, Valproic Acid, Panobinostat, Mocetinostat	-	Vorinostat
25	ENO1	Alpha-enolase	3	Zinc, Copper, Artenimol	-	-
26	S100A8	Protein S100-A8	3	Zinc, Copper	-	Calcium
27	PRDX1	Peroxiredoxin-1	3	Zinc, Copper, Artenimol	-	-

28	ADRA1D	Alpha-1D adrenergic receptor	11	Sertindole, Doxazosin, Nicardipine, Chlorpromazine, Prazosin, Clonidine, Cabergoline, Dronedarone, Verapamil, Nortriptyline, Maprotiline	-	-
29	FGFR2	Fibroblast growth factor receptor 2	5	Ponatinib, Thalidomide, Regorafenib	Heparin, Fostamatinib	-
30	FGFR1	Fibroblast growth factor receptor 1	5	Ponatinib, Sorafenib, Regorafenib	Heparin, Fostamatinib	-
31	FLT3	Receptor-type tyrosine-protein kinase FLT3	5	Ponatinib, Sorafenib, Sunitinib, Quizartinib	Fostamatinib	-
32	FLT4	Vascular endothelial growth factor receptor 3	5	Pazopanib, Sorafenib, Regorafenib, Sunitinib	Fostamatinib	-
33	TNNC1	Troponin C, slow skeletal and cardiac muscles	4	Felodipine, Levosimendan	-	Calcium, Trifluoperazine
34	RET	Proto-oncogene tyrosine-protein kinase receptor Ret	4	Ponatinib, Sorafenib, Regorafenib	Fostamatinib	-
35	DRD2	D(2) dopamine receptor	14	Sertindole, Pimozide, Triflupromazine, Fluspirilene, Fluphenazine, Lamotrigine, Chlorpromazine, Memantine, Cabergoline, Olanzapine, Nortriptyline, Maprotiline	-	Thioridazine, Trifluoperazine
36	CACNB4	Voltage-dependent L-type calcium channel subunit beta-4	5	Spironolactone, Lacidipine, Dronedarone, Verapamil	-	Nimodipine
37	CACNB3	Voltage-dependent L-type calcium channel subunit beta-3	5	Spironolactone, Lacidipine, Dronedarone, Verapamil	-	Nimodipine
38	S100A2	Protein S100-A2	3	Zinc, Copper	-	Calcium
39	HSPA8	Heat shock cognate 71 kDa protein	3	Copper, Dasatinib, Artenimol	-	-
40	P4HB	Protein disulfide-isomerase	3	Zinc, Copper, Artenimol	-	-
41	EEF1A1	Elongation factor 1-alpha 1	3	Zinc, Copper, Artenimol	-	-
42	PIK3CG	Phosphatidylinositol 4,5-bisphosphate 3-kinase catalytic subunit γ isoform	5	XL765, Quercetin	LY-294002, Fostamatinib, Wortmannin	-
43	NTRK1	High affinity nerve growth factor receptor	3	Regorafenib, Imatinib	Fostamatinib	-
44	EPHA2	Ephrin type-A receptor 2	3	Regorafenib, Dasatinib	Fostamatinib	-
45	PRKAB1	5'-AMP-activated protein kinase subunit beta-1	3	Metformin, Acetylsalicylic acid	Fostamatinib	-
46	HMGCR	3-hydroxy-3-methylglutaryl-coenzyme A reductase	5	Simvastatin, Fluvastatin, Atorvastatin, Mevastatin, Rosuvastatin	-	-
47	PPARD	Peroxisome proliferator-activated receptor delta	6	Sulindac, Valproic Acid, Fenofibrate, Troglitazone, GW-501516	-	Rosiglitazone
48	BRAF	Serine/threonine-protein kinase B-raf	4	Vemurafenib, Sorafenib, Regorafenib	Fostamatinib	-
49	HDAC1	Histone deacetylase 1	4	Zinc, Panobinostat, Mocetinostat	-	Vorinostat
50	FLT1	Vascular endothelial growth factor receptor 1	5	Pazopanib, Sorafenib, Regorafenib, Sunitinib	Fostamatinib	-
51	CACNA1F	Voltage-dependent L-type calcium channel subunit alpha-1F	5	Spironolactone, Lacidipine, Dronedarone, Verapamil	-	Nimodipine
52	ADORA2 A	Adenosine receptor A2a	5	Theophylline, Pentoxifylline, Lamotrigine	Mefloquine	Adenosine
53	HDAC3	Histone deacetylase 3	3	Panobinostat, Mocetinostat	-	Vorinostat
54	AURKB	Aurora kinase B	3	AT9283, HESPERIDIN	Fostamatinib	-
55	PPARG	Peroxisome proliferator-activated receptor gamma	10	Fenofibrate, Sulfasalazine, Valproic Acid, Resveratrol, Amiodarone, Telmisartan, Troglitazone, Curcumin	-	Indomethacin, Rosiglitazone
56	KCNJ11	ATP-sensitive inward rectifier potassium channel 11	4	Diazoxide, Levosimendan, Verapamil, Glyburide	-	-
57	ADRB1	Beta-1 adrenergic receptor	10	Lamotrigine, Spermidine, Amiodarone, Cabergoline, Salbutamol, Olanzapine, Dronedarone, Nortriptyline	Propranolol, Esmolol	-
58	UGT3A1	UDP-glucuronosyltransferase 3A1	2	Quercetin, Kaempherol	-	-
59	RPS6KA1	Ribosomal protein S6 kinase alpha-1	2	Purvalanol A	Fostamatinib	-
60	APOBR	Apolipoprotein B receptor	2	Zinc, Copper	-	-
61	APOA1	Apolipoprotein A-I	2	Zinc, Copper	-	-
62	SFPQ	Splicing factor, proline- and glutamine-rich	2	Copper, Artenimol	-	-
63	ADRA2A	Alpha-2A adrenergic receptor	11	Rilmenidine, Lamotrigine, Chlorpromazine, Prazosin, Clonidine, Cabergoline, Olanzapine, Dronedarone, Nortriptyline, Maprotiline	-	Dexmedetomidine

64	KRT1	Keratin, type II cytoskeletal 1	2	Zinc, Copper	-	-
65	IGFALS	Insulin-like growth factor-binding protein complex acid labile subunit	2	Zinc, Copper	-	-
66	DDR1	Epithelial discoidin domain-containing receptor 1	2	Imatinib	Fostamatinib	-
67	CFH	Complement factor H	2	Zinc, Copper	-	-
68	APOE	Apolipoprotein E	2	Zinc, Copper	-	-
69	C8B	Complement component C8 beta chain	2	Zinc, Copper	-	-
70	C4BPA	C4b-binding protein alpha chain	2	Zinc, Copper	-	-
71	KNG1	Kininogen-1	2	Zinc, Copper	-	-
72	APOA2	Apolipoprotein A-II	2	Zinc, Copper	-	-
73	CAST	Calpastatin	2	Artenimol	-	Calcium
74	SNCA	Alpha-synuclein	2	Resveratrol, Copper	-	-
75	YES1	Tyrosine-protein kinase Yes	2	Dasatinib	Fostamatinib	-
76	HTR2A	5-hydroxytryptamine receptor 2A	12	Sertindole, Fluspirilene, Paroxetine, Ketanserin, Fluphenazine, Lamotrigine, Chlorpromazine, Cabergoline, Olanzapine, Nortriptyline, Maprotiline	-	Thioridazine
77	NR1D2	Nuclear receptor subfamily 1 group D member 2	2	-	SR-9009, SR-9011	-
78	NR1D1	Nuclear receptor subfamily 1 group D member 1	2	-	SR-9009, SR-9011	-
79	EGFR	Epidermal growth factor receptor	6	Erlotinib, Lapatinib, Afatinib, Alvocidib, Gefitinib	Fostamatinib	-
80	KHSRP	Far upstream element-binding protein 2	2	Resveratrol, Artenimol	-	-
81	APLP1	Amyloid-like protein 1	2	Zinc, Copper	-	-
82	PDIA3	Protein disulfide-isomerase A3	2	Zinc, Copper	-	-
83	-	Ig kappa chain V-III region GOL	2	Zinc, Copper	-	-
84	PPARA	Peroxisome proliferator-activated receptor alpha	7	Resveratrol, Fenofibrate, Valproic Acid, Troglitazone, GW-501516	-	Indomethacin, Rosiglitazone
85	ESRRA	Steroid hormone receptor ERR1	3	Genistein, Troglitazone, Afimoxifene	-	-
86	FGFR3	Fibroblast growth factor receptor 3	3	Ponatinib, Pazopanib	Fostamatinib	-
87	DSP	Desmoplakin	2	Zinc, Artenimol	-	-
88	SERPINA4	Kallistatin	2	Zinc, Copper	-	-
89	KRT9	Keratin, type I cytoskeletal 9	2	Zinc, Copper	-	-
90	KRT10	Keratin, type I cytoskeletal 10	2	Zinc, Copper	-	-
91	ITIH2	Inter-alpha-trypsin inhibitor heavy	2	Zinc, Copper	-	-
92	HPR	Haptoglobin-related protein	2	Zinc, Copper	-	-
93	CHRM4	Muscarinic acetylcholine receptor M4	8	Paroxetine, Nicardipine, Lamotrigine, Olanzapine, Maprotiline, Nortriptyline, Acetylcholine	Atropine	-
94	CLU	Clusterin	2	Zinc, Copper	-	-
95	CFI	Complement factor I	2	Zinc, Copper	-	-
96	APOA4	Apolipoprotein A-IV	2	Zinc, Copper	-	-
97	AHSG	Alpha-2-HS-glycoprotein	2	Zinc, Copper	-	-
98	A1BG	Alpha-1B-glycoprotein	2	Zinc, Copper	-	-
99	S100A9	Protein S100-A9	2	Zinc	-	Calcium
100	ATP5A1	ATP synthase subunit alpha, mitochondrial	3	PICEATANNOL, Quercetin, Artenimol	-	-
101	LYN	Tyrosine-protein kinase Lyn	3	Ponatinib, Dasatinib	Fostamatinib	-
102	TFF1	Trefoil factor 1	2	Raloxifene, Afimoxifene	-	-
103	HTR2C	5-hydroxytryptamine receptor 2C	9	Sertindole, Fluphenazine, Chlorpromazine, Fluoxetine, Citalopram, Cabergoline, Olanzapine, Nortriptyline, Maprotiline	-	-
104	TEK	Angiopoietin-1 receptor	3	Ponatinib, Regorafenib	Fostamatinib	-
105	PGAM1	Phosphoglycerate mutase 1	2	Copper, Artenimol	-	-
106	HNRNPA2 B1	Heterogeneous nuclear ribonucleoproteins A2/B1	2	Copper, Artenimol	-	-
107	CYP1B1	Cytochrome P450 1B1	3	Genistein, Quercetin	-	2-Methoxyestradiol
108	ZAK	Mitogen-activated protein kinase kinase kinase MLT	2	Dasatinib	Fostamatinib	-
109	CSF1R	Macrophage colony-stimulating factor 1 receptor	3	Sunitinib, Imatinib	Fostamatinib	-

110	ESRRG	Estrogen-related receptor gamma	4	Tamoxifen, Troglitazone, Afimoxifene	Estradiol	-
111	APP	Amyloid beta A4 protein	4	Zinc, Resveratrol, Copper, Deferoxamine	-	-
112	EPHA5	Ephrin type-A receptor 5	2	Dasatinib	Fostamatinib	-
113	ATP4A	K+-transporting ATPase α chain 1	3	Omeprazole	Pantoprazole	Esomeprazole
114	FGR	Tyrosine-protein kinase Fgr	2	Dasatinib	Fostamatinib	-
115	CLIC1	Chloride intracellular channel protein 1	2	Copper, Artenimol	-	-
116	ACTG1	Actin, cytoplasmic 2	2	Copper, Artenimol	-	-
117	PRKAG3	5'-AMP-activated protein kinase subunit gamma-3	2	Acetylsalicylic acid	Fostamatinib	-
118	PRKAG2	5'-AMP-activated protein kinase subunit gamma-2	2	Acetylsalicylic acid	Fostamatinib	-
119	PRKAG1	5'-AMP-activated protein kinase subunit gamma-1	2	Acetylsalicylic acid	Fostamatinib	-
120	PRKAA2	5'-AMP-activated protein kinase catalytic subunit alpha-2	2	Acetylsalicylic acid	Fostamatinib	-
121	PRKCG	Protein kinase C gamma type	2	Tamoxifen	Fostamatinib	-
122	RPS6KA3	Ribosomal protein S6 kinase alpha-3	2	Acetylsalicylic acid	Fostamatinib	-
123	DDR2	Discoidin domain-containing receptor 2	2	Regorafenib	Fostamatinib	-
124	CHRM1	Muscarinic acetylcholine receptor M1	11	Triflupromazine, Paroxetine, Nicardipine, Lamotrigine, Chlorpromazine, Citalopram, Olanzapine, Maprotiline, Nortriptyline, Acetylcholine	Atropine	-
125	KDR	Vascular endothelial growth factor receptor 2	6	Ponatinib, Pazopanib, Sorafenib, Regorafenib, Sunitinib	Fostamatinib	-
126	SERPINA6	Corticosteroid-binding globulin	2	Zinc, Copper	-	-
127	PGLYRP2	N-acetylmuramoyl-L-alanine amidase	2	Zinc, Copper	-	-
128	CACNA1A	Voltage-dependent P/Q-type calcium channel subunit alpha-1A	3	Spironolactone, Loperamide, Verapamil	-	-
129	ORM2	Alpha-1-acid glycoprotein 2	3	Chlorpromazine, Zinc, Thalidomide	-	-
130	SCN2A	Sodium channel protein type 2 subunit alpha	3	Valproic Acid, Metergoline, Lamotrigine	-	-
131	TOP1MT	DNA topoisomerase I, mitochondrial	2	Topotecan	Irinotecan	-
132	RRM1	Ribonucleoside-diphosphate reductase large subunit	3	Hydroxyurea, Gemcitabine, Clofarabine	-	-
133	RAF1	RAF proto-oncogene serine/threonine-protein kinase	3	Sorafenib, Regorafenib	Fostamatinib	-
134	S100A4	Protein S100-A4	2	Copper	-	Trifluoperazine
135	STK17B	Serine/threonine-protein kinase 17B	2	Quercetin	Fostamatinib	-
136	ALOX5	Arachidonate 5-lipoxygenase	5	Resveratrol, Sulfasalazine, Masoprocol	Rhein, Fostamatinib	-
137	CHRM5	Muscarinic acetylcholine receptor M5	7	Paroxetine, Nicardipine, Lamotrigine, Olanzapine, Nortriptyline, Maprotiline	Atropine	-
138	PTGS1	Prostaglandin G/H synthase 1	9	Meloxicam, Sulfasalazine, Acetylsalicylic acid, Naproxen, Sulindac, Minoxidil, Resveratrol, Salicylic acid	-	Indomethacin
139	TOP2A	DNA topoisomerase 2-alpha	6	Etoposide, Idarubicin, Doxorubicin, Genistein, Dactinomycin, Daunorubicin	-	-
140	GSN	Gelsolin	2	Zinc, Copper	-	-
141	CFL1	Cofilin-1	2	Copper, Artenimol	-	-
142	PRKCI	Protein kinase C iota type	2	Tamoxifen	Fostamatinib	-
143	TNNC2	Troponin C, skeletal muscle	2	Felodipine	-	Calcium
144	MAP4	Microtubule-associated protein 4	2	Artenimol	-	Paclitaxel
145	PRKCE	Protein kinase C epsilon type	2	Tamoxifen	Fostamatinib	-
146	BIRC5	Baculoviral IAP repeat-containing protein 5	2	Reserpine	-	Berberine
147	PSMB5	Proteasome subunit beta type-5	2	Carfilzomib	-	Bortezomib
148	ADORA1	Adenosine receptor A1	4	Theophylline, Pentoxifylline, Lamotrigine	-	Adenosine
149	PTGS2	Prostaglandin G/H synthase 2	9	Meloxicam, Sulfasalazine, Acetylsalicylic acid, Naproxen, Sulindac, Resveratrol, Thalidomide, Salicylic acid	-	Indomethacin
150	PSMB1	Proteasome subunit beta type-1	2	Carfilzomib	-	Bortezomib

151	C4B	Complement C4-B	2	Zinc, Copper	-	-
152	ITK	Tyrosine-protein kinase ITK/TSK	2	Pazopanib	Fostamatinib	-
153	SLC29A1	Equilibrative nucleoside transporter 1	2	Troglitazone	Fostamatinib	-
154	VTN	Vitronectin	2	Zinc, Copper	-	-
155	AGT	Angiotensinogen	2	Zinc, Copper	-	-
156	PDE5A	cGMP-specific 3',5'-cyclic phosphodiesterase	4	Theophylline, Pentoxifylline	Sildenafil, Fostamatinib	-
157	PARP1	Poly [ADP-ribose] polymerase 1	4	Veliparib, Theophylline, Olaparib, Zinc	-	-
158	ACSL4	Long-chain-fatty-acid--CoA ligase 4	2	Troglitazone	-	Rosiglitazone
159	PON1	Serum paraoxonase/arylesterase 1	2	Zinc, Copper	-	-
160	HDAC9	Histone deacetylase 9	2	Valproic Acid, Panobinostat	-	-
161	FYN	Tyrosine-protein kinase Fyn	2	Dasatinib	Fostamatinib	-
162	PRKAB2	5'-AMP-activated protein kinase subunit beta-2	2	Acetylsalicylic acid	Fostamatinib	-
163	KRT2	Keratin, type II cytoskeletal 2 epidermal	2	Zinc, Copper	-	-
164	PPAT	Amidophosphoribosyltransferase	2	Mercaptopurine, Dasatinib	-	-
165	CACNG1	Voltage-dependent calcium channel gamma-1 subunit	3	Spironolactone, Nitrendipine, Fluspirilene	-	-
166	IKBKB	Inhibitor of nuclear factor kappa-B kinase subunit beta	3	Sulfasalazine, Acetylsalicylic acid	Fostamatinib	-
167	TTR	Transthyretin	2	Zinc, Copper	-	-
168	ERBB4	Receptor tyrosine-protein kinase erbB-4	2	Afatinib	Fostamatinib	-
169	BCR	Breakpoint cluster region protein	2	Ponatinib, Dasatinib	-	-
170	AKT1	RAC-alpha serine/threonine-protein kinase	3	Perifosine, Genistein, Resveratrol	-	-
171	CHRM3	Muscarinic acetylcholine receptor M3	9	Paroxetine, Nicardipine, Lamotrigine, Chlorpromazine, Olanzapine, Maprotiline, Nortriptyline, Acetylcholine	Atropine	-
172	CHRM2	Muscarinic acetylcholine receptor M2	9	Triflupromazine, Paroxetine, Nicardipine, Lamotrigine, Olanzapine, Maprotiline, Nortriptyline, Acetylcholine	Atropine	-
173	HDAC8	Histone deacetylase 8	3	Zinc, Panobinostat	-	Vorinostat
174	TOP2B	DNA topoisomerase 2-beta	3	Daunorubicin, Dactinomycin, Etoposide	-	-
175	RYR1	Ryanodine receptor 1	2	Dantrolene, Tetracaine	-	-
176	PARP2	Poly [ADP-ribose] polymerase 2	2	Veliparib, Olaparib	-	-
177	SLCO1B3	Solute carrier organic anion transporter family member 1B3	2	-	Clarithromycin	ginsenoside Rb1
178	CACNA2D3	Voltage-dependent calcium channel subunit alpha-2/delta-3	2	Spironolactone, Nilvadipine	-	-
179	PRKCD	Protein kinase C delta type	2	Tamoxifen	Fostamatinib	-
180	CSK	Tyrosine-protein kinase CSK	2	Dasatinib	Fostamatinib	-
181	FGFR4	Fibroblast growth factor receptor 4	2	Ponatinib	Heparin	-
182	C5	Complement C5	2	Zinc, Copper	-	-
183	BDKRB1	B1 bradykinin receptor	2	Zinc	Enalaprilat	-
184	PDCD6	Programmed cell death protein 6	2	Zinc	-	Calcium
185	PGK1	Phosphoglycerate kinase 1	2	Copper, Artenimol	-	-
186	ADRA2B	Alpha-2B adrenergic receptor	8	Chlorpromazine, Prazosin, Clonidine, Cabergoline, Olanzapine, Dronedarone, Nortriptyline, Maprotiline	-	-
187	GLRA1	Glycine receptor subunit alpha-1	4	Zinc, Taurine, Copper	Atropine	-
188	TUBB1	Tubulin beta-1 chain	3	Cabazitaxel, Colchicine	-	Paclitaxel
189	ERBB2	Receptor tyrosine-protein kinase erbB-2	3	Lapatinib, Afatinib	Fostamatinib	-
190	SCN5A	Sodium channel protein type 5 subunit alpha	5	Carbamazepine, Verapamil, Valproic Acid, Ranolazine, Procainamide	-	-
191	CEBPB	CCAAT/enhancer-binding protein beta	1	Quercetin	-	-
192	NNMT	Nicotinamide N-methyltransferase	1	Niacin	-	-

193	MAP2K3	Dual specificity mitogen-activated protein kinase kinase 3	1	-	Fostamatinib	-
194	LRRK2	Leucine-rich repeat serine/threonine-protein kinase 2	1	-	Fostamatinib	-
195	HIST1H1E	Histone H1.4	1	Copper	-	-
196	IGHA1	Ig alpha-1 chain C region	1	Zinc	-	-
197	PZP	Pregnancy zone protein	1	Zinc	-	-
198	SERPIND1	Heparin cofactor 2	2	Zinc, Copper	-	-
199	ROCK2	Rho-associated protein kinase 2	2	Fasudil	Fostamatinib	-
200	RIOK2	Serine/threonine-protein kinase RIO2	1	-	Fostamatinib	-
201	ATP1A2	Sodium/potassium-transporting ATPase subunit alpha-2	1	Ouabain	-	-
202	ATP5C1	ATP synthase subunit gamma, mitochondrial	2	PICEATANNOL, Quercetin	-	-
203	HSPA2	Heat shock-related 70 kDa protein 2	1	Quercetin	-	-
204	TUBG1	Tubulin gamma-1 chain	1	Vinblastine	-	-
205	TUBE1	Tubulin epsilon chain	1	Vinblastine	-	-
206	C3	Complement C3	2	Zinc, Copper	-	-
207	TUBD1	Tubulin delta chain	1	Vinblastine	-	-
208	ABCC5	Multidrug resistance-associated protein 5	1	Curcumin	-	-
209	BCR/ABL fusion	BCR/ABL fusion protein isoform X9	1	Imatinib	-	-
210	TP53	Cellular tumor antigen p53	2	Zinc, Acetylsalicylic acid	-	-
211	EPX	Eosinophil peroxidase	1	-	-	Melatonin
212	DRD1	D(1A) dopamine receptor	7	Triflupromazine, Fluphenazine, Lamotrigine, Chlorpromazine, Cabergoline, Olanzapine	-	Thioridazine
213	RORB	Nuclear receptor ROR-beta	1	-	-	Melatonin
214	KCNH7	Potassium voltage-gated channel subfamily H member 7	2	Prazosin, Doxazosin	-	-
215	ASMT	Acetylserotonin O-methyltransferase	1	-	-	Melatonin
216	CYP11B2	Cytochrome P450 11B2, mitochondrial	1	Spironolactone	-	-
217	UGT1A1	UDP-glucuronosyltransferase 1-1	1	-	Fostamatinib	-
218	HIF1A	Hypoxia-inducible factor 1-alpha	2	-	PX-478	2-Methoxyestradiol
219	CPN2	Carboxypeptidase N subunit 2	1	Zinc	-	-
220	CPN1	Carboxypeptidase N catalytic chain	1	Zinc	-	-
221	HCK	Tyrosine-protein kinase HCK	2	Quercetin	Fostamatinib	-
222	CASP8	Caspase-8	1	Bardoxolone	-	-
223	APOC2	Apolipoprotein C-II	1	Copper	-	-
224	PRKCZ	Protein kinase C zeta type	1	Tamoxifen	-	-
225	PRKCQ	Protein kinase C theta type	2	Tamoxifen	Fostamatinib	-
226	EBP	3-beta-hydroxysteroid-Delta(8),Delta(7)-isomerase	1	Tamoxifen	-	-
227	PHB2	Prohibitin-2	1	Capsaicin	-	-
228	NIM1K	Serine/threonine-protein kinase NIM1	1	-	Fostamatinib	-
229	APCS	Serum amyloid P-component	2	Zinc, Copper	-	-
230	RIC3	Protein RIC-3	1	Theophylline	-	-
231	NQO2	Ribosyldihydronicotinamide dehydrogenase [quinone]	3	Quercetin, Resveratrol	-	Melatonin
232	PDE3A	cGMP-inhibited 3',5'-cyclic phosphodiesterase A	3	Theophylline, Cilostazol, Levosimendan	-	-
233	AFM	Afamin	1	Copper	-	-
234	TLR9	Toll-like receptor 9	2	Hydroxychloroquine	Chloroquine	-
235	CPNE1	Copine-1	1	Theophylline	-	-
236	ZYX	Zyxin	1	Artenimol	-	-
237	SRSF4	Serine/arginine-rich splicing factor 4	1	Artenimol	-	-
238	CP	Ceruloplasmin	2	Zinc	-	Calcium
239	RPS28	40S ribosomal protein S28	1	Artenimol	-	-
240	RPS19	40S ribosomal protein S19	1	Artenimol	-	-
241	PSMB8	Proteasome subunit beta type-8	1	Carfilzomib	-	-

242	HSPA5	78 kDa glucose-regulated protein	2	Copper, Acetylsalicylic acid	-	-
243	RPS17	40S ribosomal protein S17	1	Artenimol	-	-
244	RPL35	60S ribosomal protein L35	1	Artenimol	-	-
245	PTK2B	Protein-tyrosine kinase 2-beta	2	Genistein	Fostamatinib	-
246	CDK8	Cyclin-dependent kinase 8	1	Alvocidib	-	-
247	NR4A3	Nuclear receptor subfamily 4 group A member 3	1	Dasatinib	-	-
248	IQGAP1	Ras GTPase-activating-like protein IQGAP1	1	Artenimol	-	-
249	PRKAA1	5'-AMP-activated protein kinase catalytic subunit alpha-1	2	Acetylsalicylic acid	Fostamatinib	-
250	HP1BP3	Heterochromatin protein 1-binding protein 3	1	Artenimol	-	-
251	HNRNPD	Heterogeneous nuclear ribonucleoprotein D0	1	Artenimol	-	-
252	RPS2	40S ribosomal protein S2	1	Copper	-	-
253	MTNR1A	Melatonin receptor type 1A	2	Resveratrol	-	Melatonin
254	CSRP1	Cysteine and glycine-rich protein 1	1	Artenimol	-	-
255	KRT5	Keratin, type II cytoskeletal 5	1	Zinc	-	-
256	KCNH6	Potassium voltage-gated channel subfamily H member 6	2	Prazosin, Doxazosin	-	-
257	MAP4K4	Mitogen-activated protein kinase kinase kinase kinase 4	1	-	Fostamatinib	-
258	EIF2AK2	Interferon-induced, double-stranded RNA-activated protein kinase	1	-	Fostamatinib	-
259	SIK2	Serine/threonine-protein kinase SIK2	1	-	Fostamatinib	-
260	ABL2	Abelson tyrosine-protein kinase 2	2	Dasatinib	Fostamatinib	-
261	SEMG1	Semenogelin-1	1	Zinc	-	-
262	ACAA1	3-ketoacyl-CoA thiolase, peroxisomal	1	Trimetazidine	-	-
263	LUM	Lumican	1	Copper	-	-
264	ESR1	Estrogen receptor	10	Tamoxifen, Zinc, Genistein, Resveratrol, Fulvestrant, Quercetin, Raloxifene, Afimoxifene	Estradiol	Melatonin
265	ADORA3	Adenosine receptor A3	2	-	Fostamatinib	Adenosine
266	LRG1	Leucine-rich alpha-2-glycoprotein	1	Copper	-	-
267	PEBP1	Phosphatidylethanolamine-binding protein 1	1	Copper	-	-
268	MTNR1B	Melatonin receptor type 1B	2	Resveratrol	-	Melatonin
269	C9	Complement component C9	1	Copper	-	-
270	NT5E	5'-nucleotidase	1	Pentoxifylline	-	-
271	PLCL1	Inactive phospholipase C-like protein 1	1	-	Quinacrine	-
272	ADORA2B	Adenosine receptor A2b	2	Theophylline	-	Adenosine
273	PLA2G6	85/88 kDa calcium-independent phospholipase A2	1	-	Quinacrine	-
274	ITGB2	Integrin beta-2	1	Simvastatin	-	-
275	PARK7	Protein deglycase DJ-1	1	Copper	-	-
276	PDE1A	Calcium/calmodulin-dependent 3',5'-cyclic nucleotide phosphodiesterase 1A	2	Felodipine, Nicardipine	-	-
277	IMPA2	Inositol monophosphatase 2	1	-	-	Lithium
278	IAPP	Islet amyloid polypeptide	1	Copper	-	-
279	SNRK	SNF-related serine/threonine-protein kinase	1	-	Fostamatinib	-
280	COMP	Cartilage oligomeric matrix protein	1	-	-	Calcium
281	SF3B3	Splicing factor 3B subunit 3	1	Quercetin	-	-
282	STAT5B	Signal transducer and activator of	1	Dasatinib	-	-
283	HBA1	Hemoglobin subunit alpha	3	Zinc, Copper	Mefloquine	-
284	STK17A	Serine/threonine-protein kinase 17A	1	-	Fostamatinib	-
285	STK16	Serine/threonine-protein kinase 16	1	-	Fostamatinib	-
286	SRMS	Tyrosine-protein kinase Srms	1	-	Fostamatinib	-
287	SGK3	Serine/threonine-protein kinase Sgk3	1	-	Fostamatinib	-

288	SBK3	Uncharacterized serine/threonine-protein kinase SBK3	1	-	Fostamatinib	-
289	SBK1	Serine/threonine-protein kinase SBK1	1	-	Fostamatinib	-
290	ROS1	Proto-oncogene tyrosine-protein kinase ROS	1	-	Fostamatinib	-
291	RIPK4	Receptor-interacting serine/threonine-protein kinase 4	1	-	Fostamatinib	-
292	RIPK1	Receptor-interacting serine/threonine-protein kinase 1	1	-	Fostamatinib	-
293	RIOK3	Serine/threonine-protein kinase RIO3	1	-	Fostamatinib	-
294	RIOK1	Serine/threonine-protein kinase RIO1	1	-	Fostamatinib	-
295	PRPF4B	Serine/threonine-protein kinase PRP4 homolog	1	-	Fostamatinib	-
296	NR1I3	Nuclear receptor subfamily 1 group I member 3	3	Loperamide, Resveratrol	Clotrimazole	-
297	PRKG2	cGMP-dependent protein kinase 2	1	-	Fostamatinib	-
298	PLK3	Serine/threonine-protein kinase PLK3	1	-	Fostamatinib	-
299	PLK2	Serine/threonine-protein kinase PLK2	1	-	Fostamatinib	-
300	PKN1	Serine/threonine-protein kinase N1	1	-	Fostamatinib	-
301	PKMYT1	Membrane-associated tyrosine- and threonine-specific cdc2-inhibitory kinase	1	-	Fostamatinib	-
302	PIP4K2C	Phosphatidylinositol 5-phosphate 4-kinase type-2 gamma	1	-	Fostamatinib	-
303	PIP4K2B	Phosphatidylinositol 5-phosphate 4-kinase type-2 beta	1	-	Fostamatinib	-
304	PIM3	Serine/threonine-protein kinase pim-3	1	-	Fostamatinib	-
305	PIK3C2G	Phosphatidylinositol 4-phosphate 3-kinase C2 domain-containing subunit gamma	1	-	Fostamatinib	-
306	PAK5	Serine/threonine-protein kinase PAK 5	1	-	Fostamatinib	-
307	PAK6	Serine/threonine-protein kinase PAK 6	1	-	Fostamatinib	-
308	PAK4	Serine/threonine-protein kinase PAK 4	1	-	Fostamatinib	-
309	PAK3	Serine/threonine-protein kinase PAK 3	1	-	Fostamatinib	-
310	PAK2	Serine/threonine-protein kinase PAK 2	1	-	Fostamatinib	-
311	PAK1	Serine/threonine-protein kinase PAK 1	1	-	Fostamatinib	-
312	OXSR1	Serine/threonine-protein kinase OSR1	1	-	Fostamatinib	-
313	NUAK2	NUAK family SNF1-like kinase 2	1	-	Fostamatinib	-
314	NUAK1	NUAK family SNF1-like kinase 1	1	-	Fostamatinib	-
315	JAK2	Tyrosine-protein kinase JAK2	2	Ruxolitinib	Fostamatinib	-
316	PTGDR2	Prostaglandin D2 receptor 2	2	Sulindac	-	Indomethacin
317	NTRK3	NT-3 growth factor receptor	1	-	Fostamatinib	-
318	SERPINE1	Plasminogen activator inhibitor 1	2	Copper, Troglitazone	-	-
319	NEK9	Serine/threonine-protein kinase Nek9	1	-	Fostamatinib	-
320	CALR	Calreticulin	2	Copper	-	Melatonin
321	NEK5	Serine/threonine-protein kinase Nek5	1	-	Fostamatinib	-
322	TUBA1A	Tubulin alpha-1A chain	2	Vinblastine, Artenimol	-	-
323	NEK4	Serine/threonine-protein kinase Nek4	1	-	Fostamatinib	-
324	HSP90B1	Endoplasmin	2	Geldanamycin, Copper	-	-
325	CDK1	Cyclin-dependent kinase 1	2	Alvocidib	Fostamatinib	-
326	NEK3	Serine/threonine-protein kinase Nek3	1	-	Fostamatinib	-
327	AKR1C3	Aldo-keto reductase family 1 member C3	2	-	Rutin	Indomethacin
328	NEK1	Serine/threonine-protein kinase Nek1	1	-	Fostamatinib	-
329	MYO3A	Myosin-IIIa	1	-	Fostamatinib	-
330	MYLK4	Myosin light chain kinase family member 4	1	-	Fostamatinib	-
331	MYLK3	Myosin light chain kinase 3	1	-	Fostamatinib	-
332	MYLK	Myosin light chain kinase, smooth muscle	1	-	Fostamatinib	-
333	MUSK	Muscle, skeletal receptor tyrosine-protein kinase	1	-	Fostamatinib	-

334	MST1R	Macrophage-stimulating protein receptor	1	-	Fostamatinib	-
335	MKNK2	MAP kinase-interacting serine/threonine-protein kinase 2	1	-	Fostamatinib	-
336	MKNK1	MAP kinase-interacting serine/threonine-protein kinase 1	1	-	Fostamatinib	-
337	MINK1	Misshapen-like kinase 1	1	-	Fostamatinib	-
338	MATK	Megakaryocyte-associated tyrosine-protein kinase	1	-	Fostamatinib	-
339	MAST1	Microtubule-associated serine/threonine-protein kinase 1	1	-	Fostamatinib	-
340	CLASP1	CLIP-associating protein 1	1	-	Fostamatinib	-
341	MARK4	MAP/microtubule affinity-regulating kinase 4	1	-	Fostamatinib	-
342	MARK3	MAP/microtubule affinity-regulating kinase 3	1	-	Fostamatinib	-
343	MARK2	Serine/threonine-protein kinase MARK2	1	-	Fostamatinib	-
344	MARK1	Serine/threonine-protein kinase MARK1	1	-	Fostamatinib	-
345	NPC1L1	Niemann-Pick C1-like protein 1	1	Ezetimibe	-	-
346	MAPK7	Mitogen-activated protein kinase 7	1	-	Fostamatinib	-
347	MAPK15	Mitogen-activated protein kinase 15	1	-	Fostamatinib	-
348	MAP4K5	Mitogen-activated protein kinase kinase kinase kinase 5	1	-	Fostamatinib	-
349	MAP4K3	Mitogen-activated protein kinase kinase kinase kinase 3	1	-	Fostamatinib	-
350	MAP4K2	Mitogen-activated protein kinase kinase kinase kinase 2	1	-	Fostamatinib	-
351	MAP4K1	Mitogen-activated protein kinase kinase kinase kinase 1	1	-	Fostamatinib	-
352	MAP3K6	Mitogen-activated protein kinase kinase kinase 6	1	-	Fostamatinib	-
353	DRD5	D(1B) dopamine receptor	4	Chlorpromazine, Olanzapine, Lamotrigine, Cabergoline	-	-
354	ADRB3	Beta-3 adrenergic receptor	4	Salbutamol, Olanzapine, Nortriptyline	Propranolol	-
355	MAP3K4	Mitogen-activated protein kinase kinase kinase 4	1	-	Fostamatinib	-
356	MAP3K3	Mitogen-activated protein kinase kinase kinase 3	1	-	Fostamatinib	-
357	MAP3K15	Mitogen-activated protein kinase kinase kinase 15	1	-	Fostamatinib	-
358	MAP3K13	Mitogen-activated protein kinase kinase kinase 13	1	-	Fostamatinib	-
359	MAP3K12	Mitogen-activated protein kinase kinase kinase 12	1	-	Fostamatinib	-
360	MAP3K11	Mitogen-activated protein kinase kinase kinase 11	1	-	Fostamatinib	-
361	MAP3K10	Mitogen-activated protein kinase kinase kinase 10	1	-	Fostamatinib	-
362	MAP2K6	Dual specificity mitogen-activated protein kinase kinase 6	1	-	Fostamatinib	-
363	MAP2K5	Dual specificity mitogen-activated protein kinase kinase 5	1	-	Fostamatinib	-
364	LTK	Leukocyte tyrosine kinase receptor	1	-	Fostamatinib	-
365	LIMK2	LIM domain kinase 2	1	-	Fostamatinib	-
366	LATS1	Serine/threonine-protein kinase LATS1	1	-	Fostamatinib	-
367	CSNK2A1	Casein kinase II subunit alpha	4	Quercetin, Resveratrol	Fostamatinib	Emodin
368	SIK3	Serine/threonine-protein kinase SIK3	1	-	Fostamatinib	-
369	IRAK3	Interleukin-1 receptor-associated kinase 3	1	-	Fostamatinib	-
370	IRAK1	Interleukin-1 receptor-associated kinase 1	1	-	Fostamatinib	-
371	INSRR	Insulin receptor-related protein	1	-	Fostamatinib	-
372	IKBKE	Inhibitor of nuclear factor kappa-B kinase subunit epsilon	1	-	Fostamatinib	-

373	ICK	Serine/threonine-protein kinase ICK	1	-	Fostamatinib	-
374	HIPK3	Homeodomain-interacting protein kinase 3	1	-	Fostamatinib	-
375	HIPK2	Homeodomain-interacting protein kinase 2	1	-	Fostamatinib	-
376	GSK3A	Glycogen synthase kinase-3 alpha	1	-	Fostamatinib	-
377	GAK	Cyclin-G-associated kinase	1	-	Fostamatinib	-
378	FES	Tyrosine-protein kinase Fes/Fps	1	-	Fostamatinib	-
379	FER	Tyrosine-protein kinase Fer	1	-	Fostamatinib	-
380	EPHB6	Ephrin type-B receptor 6	1	-	Fostamatinib	-
381	EPHB1	Ephrin type-B receptor 1	1	-	Fostamatinib	-
382	EPHA8	Ephrin type-A receptor 8	1	-	Fostamatinib	-
383	EPHA6	Ephrin type-A receptor 6	1	-	Fostamatinib	-
384	EPHA4	Ephrin type-A receptor 4	1	-	Fostamatinib	-
385	PLK1	Serine/threonine-protein kinase PLK1	2	-	Fostamatinib,	-
386	ATP5B	ATP synthase subunit beta, mitochondrial	2	PICEATANNOL, Quercetin	-	-
387	EPHA3	Ephrin type-A receptor 3	1	-	Fostamatinib	-
388	EPHA1	Ephrin type-A receptor 1	1	-	Fostamatinib	-
389	JAK1	Tyrosine-protein kinase JAK1	2	Ruxolitinib	Fostamatinib	-
390	EIF2AK4	eIF-2-alpha kinase GCN2	1	-	Fostamatinib	-
391	HDAC4	Histone deacetylase 4	2	Zinc, Panobinostat	-	-
392	EIF2AK1	Eukaryotic translation initiation factor 2-alpha kinase 1	1	-	Fostamatinib	-
393	DYRK1B	Dual specificity tyrosine-phosphorylation-regulated kinase 1B	1	-	Fostamatinib	-
394	GAPDHS	Glyceraldehyde-3-phosphate dehydrogenase, testis-specific	2	Zinc, Artenimol	-	-
395	DCLK3	Serine/threonine-protein kinase DCLK3	1	-	Fostamatinib	-
396	DCLK2	Serine/threonine-protein kinase DCLK2	1	-	Fostamatinib	-
397	PDE1B	Calcium/calmodulin-dependent 3',5'-cyclic nucleotide phosphodiesterase 1B	2	Felodipine, Nicardipine	-	-
398	DCLK1	Serine/threonine-protein kinase DCLK1	1	-	Fostamatinib	-
399	LCK	Tyrosine-protein kinase Lck	3	Ponatinib, Dasatinib	Fostamatinib	-
400	DAPK2	Death-associated protein kinase 2	1	-	Fostamatinib	-
401	PIK3CD	Phosphatidylinositol 4,5-bisphosphate 3-kinase catalytic subunit delta isoform	2	XL765	Fostamatinib	-
402	CSNK1A1	Casein kinase I isoform alpha	1	-	Fostamatinib	-
403	CLK4	Dual specificity protein kinase CLK4	1	-	Fostamatinib	-
404	PIK3CA	Phosphatidylinositol 4,5-bisphosphate 3-kinase catalytic subunit alpha isoform	2	XL765	Wortmannin	-
405	CLK2	Dual specificity protein kinase CLK2	1	-	Fostamatinib	-
406	CIT	Citron Rho-interacting kinase	1	-	Fostamatinib	-
407	HDAC6	Histone deacetylase 6	2	Panobinostat	-	Vorinostat
408	CDKL2	Cyclin-dependent kinase-like 2	1	-	Fostamatinib	-
409	CDK4	Cyclin-dependent kinase 4	2	Alvocidib	Fostamatinib	-
410	CDKL1	Cyclin-dependent kinase-like 1	1	-	Fostamatinib	-
411	CDC42BPG	Serine/threonine-protein kinase MRCK gamma	1	-	Fostamatinib	-
412	ALDOA	Fructose-bisphosphate aldolase A	2	Zinc, Artenimol	-	-
413	CSN3	Kappa-casein	1	-	Fostamatinib	-
414	CAMKK2	Calcium/calmodulin-dependent protein kinase kinase 2	1	-	Fostamatinib	-
415	BTK	Tyrosine-protein kinase BTK	2	Dasatinib	Fostamatinib	-
416	CAMKK1	Calcium/calmodulin-dependent protein kinase kinase 1	1	-	Fostamatinib	-
417	CAMK1	Calcium/calmodulin-dependent protein kinase type 1	1	-	Fostamatinib	-

418	SERPINA1	Alpha-1-antitrypsin	2	Zinc, Copper	-	-
419	BMX	Cytoplasmic tyrosine-protein kinase BMX	1	-	Fostamatinib	-
420	BMPR1B	Bone morphogenetic protein receptor type-1B	1	-	Fostamatinib	-
421	ALK	ALK tyrosine kinase receptor	2	Crizotinib	Fostamatinib	-
422	BMP2K	BMP-2-inducible protein kinase	1	-	Fostamatinib	-
423	BLK	Tyrosine-protein kinase Blk	1	-	Fostamatinib	-
424	TLR7	Toll-like receptor 7	2	Imiquimod, Hydroxychloroquine	-	-
425	GPI	Glucose-6-phosphate isomerase	2	Copper, Artenimol	-	-
426	RXRG	Retinoic acid receptor RXR-gamma	2	Bexarotene	-	Rosiglitazone
427	PRKCA	Protein kinase C alpha type	2	Perifosine, Tamoxifen	-	-
428	AXL	Tyrosine-protein kinase receptor UFO	1	-	Fostamatinib	-
429	ESR2	Estrogen receptor beta	6	Genistein, Afimoxifene, Quercetin, Raloxifene,	Estradiol	-
430	HRH4	Histamine H4 receptor	2	Chlorpromazine, Olanzapine	-	-
431	ANKK1	Ankyrin repeat and protein kinase domain-containing protein 1	1	-	Fostamatinib	-
432	COQ8B	Atypical kinase COQ8B, mitochondrial	1	-	Fostamatinib	-
433	AAK1	AP2-associated protein kinase 1	1	-	Fostamatinib	-
434	MAP3K19	Mitogen-activated protein kinase kinase kinase 19	1	-	Fostamatinib	-
435	ULK3	Serine/threonine-protein kinase ULK3	1	-	Fostamatinib	-
436	ULK2	Serine/threonine-protein kinase ULK2	1	-	Fostamatinib	-
437	ULK1	Serine/threonine-protein kinase ULK1	1	-	Fostamatinib	-
438	TYRO3	Tyrosine-protein kinase receptor TYRO3	1	-	Fostamatinib	-
439	TXK	Tyrosine-protein kinase TXK	1	-	Fostamatinib	-
440	TSSK1B	Testis-specific serine/threonine-protein kinase 1	1	-	Fostamatinib	-
441	TNNI3K	Serine/threonine-protein kinase TNNI3K	1	-	Fostamatinib	-
442	TNK1	Non-receptor tyrosine-protein kinase TNK1	1	-	Fostamatinib	-
443	TNIK	TRAF2 and NCK-interacting protein kinase	1	-	Fostamatinib	-
444	TLK2	Serine/threonine-protein kinase tousled-like 2	1	-	Fostamatinib	-
445	HTR7	5-hydroxytryptamine receptor 7	4	Chlorpromazine, Olanzapine, Maprotiline, Cabergoline	-	-
446	TLK1	Serine/threonine-protein kinase tousled-like 1	1	-	Fostamatinib	-
447	TIE1	Tyrosine-protein kinase receptor Tie-1	1	-	Fostamatinib	-
448	TESK1	Dual specificity testis-specific protein kinase 1	1	-	Fostamatinib	-
449	TBK1	Serine/threonine-protein kinase TBK1	1	-	Fostamatinib	-
450	TAOK3	Serine/threonine-protein kinase TAO3	1	-	Fostamatinib	-
451	TAOK1	Serine/threonine-protein kinase TAO1	1	-	Fostamatinib	-
452	STK39	STE20/SPS1-related proline-alanine-rich protein kinase	1	-	Fostamatinib	-
453	STK38L	Serine/threonine-protein kinase 38-like	1	-	Fostamatinib	-
454	STK38	Serine/threonine-protein kinase 38	1	-	Fostamatinib	-
455	STK36	Serine/threonine-protein kinase 36	1	-	Fostamatinib	-
456	AURKA	Aurora kinase A	3	Alisertib, AT9283	Fostamatinib	-
457	STK35	Serine/threonine-protein kinase 35	1	-	Fostamatinib	-
458	STK33	Serine/threonine-protein kinase 33	1	-	Fostamatinib	-
459	HRH1	Histamine H1 receptor	9	Flunarizine, Lamotrigine, Chlorpromazine, Clemastine, Citalopram, Olanzapine, Nortriptyline, Maprotiline	Rupatadine	-
460	HTR6	5-hydroxytryptamine receptor 6	4	Sertindole, Chlorpromazine, Olanzapine, Nortriptyline	-	-

461	TEC	Tyrosine-protein kinase Tec	1	-	Fostamatinib	-
462	STK3	Serine/threonine-protein kinase 3	1	-	Fostamatinib	-
463	STK24	Serine/threonine-protein kinase 24	1	-	Fostamatinib	-
464	PIM1	Serine/threonine-protein kinase pim-1	3	Quercetin	LY-294002, Fostamatinib	-
465	COQ8A	Atypical kinase COQ8A, mitochondrial	1	-	Fostamatinib	-
466	RPS6KA6	Ribosomal protein S6 kinase alpha-6	1	-	Fostamatinib	-
467	CACNA1E	Voltage-dependent R-type calcium channel subunit alpha-1E	1	Lamotrigine	-	-
468	DDX5	Probable ATP-dependent RNA helicase DDX5	1	Artenimol	-	-
469	MYH9	Myosin-9	1	Artenimol	-	-
470	SRSF1	Serine/arginine-rich splicing factor 1	1	Copper	-	-
471	TAGLN2	Transgelin-2	1	Artenimol	-	-
472	SNRPD2	Small nuclear ribonucleoprotein Sm D2	1	Artenimol	-	-
473	SF1	Splicing factor 1	1	Artenimol	-	-
474	RPS9	40S ribosomal protein S9	1	Artenimol	-	-
475	RPS8	40S ribosomal protein S8	1	Artenimol	-	-
476	ADRA2C	Alpha-2C adrenergic receptor	7	Chlorpromazine, Clonidine, Cabergoline, Olanzapine, Dronedarone, Nortriptyline, Maprotiline	-	-
477	RPS6	40S ribosomal protein S6	1	Artenimol	-	-
478	RPS5	40S ribosomal protein S5	1	Artenimol	-	-
479	RPS18	40S ribosomal protein S18	1	Artenimol	-	-
480	RPS13	40S ribosomal protein S13	1	Artenimol	-	-
481	RPL4	60S ribosomal protein L4	1	Artenimol	-	-
482	ALPP	Alkaline phosphatase, placental type	1	-	-	Calcium
483	CAMK1D	Calcium/calmodulin-dependent protein kinase type 1D	1	-	Fostamatinib	-
484	RPL18	60S ribosomal protein L18	1	Artenimol	-	-
485	MERTK	Tyrosine-protein kinase Mer	1	-	Fostamatinib	-
486	CAMK1G	Calcium/calmodulin-dependent protein kinase type 1G	1	-	Fostamatinib	-
487	RPL14	60S ribosomal protein L14	1	Artenimol	-	-
488	EPHA7	Ephrin type-A receptor 7	1	-	Fostamatinib	-
489	PFN1	Profilin-1	1	Artenimol	-	-
490	RPL10	60S ribosomal protein L10	1	Artenimol	-	-
491	STK26	Serine/threonine-protein kinase 26	1	-	Fostamatinib	-
492	CDK16	Cyclin-dependent kinase 16	1	-	Fostamatinib	-
493	FLNA	Filamin-A	1	Artenimol	-	-
494	DYRK1A	Dual specificity tyrosine-phosphorylation-regulated kinase 1A	1	-	Fostamatinib	-
495	CSNK2A2	Casein kinase II subunit alpha'	1	-	Fostamatinib	-
496	ATP5O	ATP synthase subunit O, mitochondrial	1	Artenimol	-	-
497	ERN1	Serine/threonine-protein kinase/endoribonuclease IRE1	1	-	Fostamatinib	-
498	CCT3	T-complex protein 1 subunit gamma	1	Artenimol	-	-
499	APLP2	Amyloid-like protein 2	1	Zinc	-	-
500	CAMK2B	Calcium/calmodulin-dependent protein kinase type II subunit beta	1	-	Fostamatinib	-
501	TTK	Dual specificity protein kinase TTK	1	-	Fostamatinib	-
502	SERPINA3	Alpha-1-antichymotrypsin	1	Zinc	-	-
503	S100A7	Protein S100-A7	1	Zinc	-	-
504	NTRK2	BDNF/NT-3 growth factors receptor	1	-	Fostamatinib	-

505	KRT6A	Keratin, type II cytoskeletal 6A	1	Zinc	-	-
506	PTGR2	Prostaglandin reductase 2	1	-	-	Indomethacin
507	PDE6H	Retinal cone rhodopsin-sensitive cGMP 3',5'-cyclic phosphodiesterase subunit gamma	1	-	Sildenafil	-
508	KRT16	Keratin, type I cytoskeletal 16	1	Zinc	-	-
509	CYP1A1	Cytochrome P450 1A1	1	-	-	2-Methoxyestradiol
510	CYP2C19	Cytochrome P450 2C19	1	Isoniazid	-	-
511	KRT14	Keratin, type I cytoskeletal 14	1	Zinc	-	-
512	MLNR	Motilin receptor	1	-	Erythromycin	-
513	TLR8	Toll-like receptor 8	1	Imiquimod	-	-
514	JUP	Junction plakoglobin	1	Zinc	-	-
515	NEK2	Serine/threonine-protein kinase Nek2	1	-	Fostamatinib	-
516	NPEPPS	Puromycin-sensitive aminopeptidase	1	Artenimol	-	-
517	JCHAIN	Immunoglobulin J chain	1	Zinc	-	-
518	TUBB6	Tubulin beta-6 chain	1	Artenimol	-	-
519	MAPK13	Mitogen-activated protein kinase 13	1	-	Fostamatinib	-
520	ITIH4	Inter-alpha-trypsin inhibitor heavy chain H4	1	Zinc	-	-
521	MAPK11	Mitogen-activated protein kinase 11	1	Regorafenib	-	-
522	ITIH3	Inter-alpha-trypsin inhibitor heavy chain H3	1	Zinc	-	-
523	AZGP1	Zinc-alpha-2-glycoprotein	1	Copper	-	-
524	APOH	Beta-2-glycoprotein 1	1	Copper	-	-
525	ITIH1	Inter-alpha-trypsin inhibitor heavy chain H1	1	Zinc	-	-
526	HCAR3	Hydroxycarboxylic acid receptor 3	1	Niacin	-	-
527	CYP19A1	Aromatase	2	Nicotine	-	2-Methoxyestradiol
528	FGF1	Fibroblast growth factor 1	2	Pazopanib	Heparin	-
529	ALDH7A1	Alpha-aminoadipic semialdehyde dehydrogenase	1	Artenimol	-	-
530	NFKB2	Nuclear factor NF-kappa-B p100 subunit	2	Glucosamine, Acetylsalicylic acid	-	-
531	EPHB4	Ephrin type-B receptor 4	2	Dasatinib	Fostamatinib	-
532	ALB	Serum albumin	2	Artenimol	Erythromycin	-
533	IGLV3-21	Immunoglobulin lambda variable 3-21	1	Zinc	-	-
534	CASK	Peripheral plasma membrane protein CASK	1	-	Fostamatinib	-
535	SCN1A	Sodium channel protein type 1 subunit alpha	2	Valproic Acid, Dronedarone	-	-
536	CACNA1B	Voltage-dependent N-type calcium channel subunit alpha-1B	2	Spironolactone, Verapamil	-	-
537	PTGES3	Prostaglandin E synthase 3	1	Copper	-	-
538	IGKV1-17	Immunoglobulin kappa variable 1-17	1	Zinc	-	-
539	CDK9	Cyclin-dependent kinase 9	1	Alvocidib	-	-
540	HDAC5	Histone deacetylase 5	1	Panobinostat	-	-
541	HRNR	Hornerin	1	Zinc	-	-
542	PIK3CB	Phosphatidylinositol 4,5-bisphosphate 3-kinase catalytic subunit beta isoform	1	XL765	-	-
543	RPSA	40S ribosomal protein SA	1	Copper	-	-
544	FCN3	Ficolin-3	1	Zinc	-	-
545	PSMB2	Proteasome subunit beta type-2	1	Carfilzomib	-	-
546	PTK6	Protein-tyrosine kinase 6	1	-	Fostamatinib	-
547	DCD	Dermcidin	1	Zinc	-	-
548	KCNJ5	G protein-activated inward rectifier potassium channel 4	1	Glyburide	-	-
549	GSTA2	Glutathione S-transferase A2	1	-	Chloroquine	-

550	C8A	Complement component C8 alpha chain	1	Zinc	-	-
551	PYGB	Glycogen phosphorylase, brain form	1	Alvocidib	-	-
552	ZAP70	Tyrosine-protein kinase ZAP-70	1	-	Fostamatinib	-
553	BRCC3	Lys-63-specific deubiquitinase BRCC36	1	Zinc	-	-
554	IMPA1	Inositol monophosphatase 1	1	-	-	Lithium
555	PF4	Platelet factor 4	1	-	Heparin	-
556	C4BPB	C4b-binding protein beta chain	1	Zinc	-	-
557	SERPINF2	Alpha-2-antiplasmin	1	Copper	-	-
558	AURKC	Aurora kinase C	1	-	Fostamatinib	-
559	APOL1	Apolipoprotein L1	1	Zinc	-	-
560	DDX39B	Spliceosome RNA helicase DDX39B	1	Artenimol	-	-
561	RET	RET proto-oncogene	1	Imatinib	-	-
562	SERPINF1	Pigment epithelium-derived factor	1	Copper	-	-
563	ANPEP	Aminopeptidase N	1	Ezetimibe	-	-
564	SLC2A1	Solute carrier family 2, facilitated glucose transporter member 1	1	Resveratrol	-	-
565	IGLL1	Immunoglobulin lambda-like polypeptide 1	1	Copper	-	-
566	mmp20	Matrix metalloproteinase-25	1	Fenofibrate	-	-
567	LDHA	L-lactate dehydrogenase A chain	2	Copper, Artenimol	-	-
568	PPIA	Peptidyl-prolyl cis-trans isomerase A	2	Copper, Artenimol	-	-
569	TPM1	Tropomyosin alpha-1 chain	1	Artenimol	-	-
570	CBX5	Chromobox protein homolog 5	1	Copper	-	-
571	PPBP	Platelet basic protein	1	Copper	-	-
572	ATP5L	ATP synthase subunit g, mitochondrial	1	Artenimol	-	-
573	ORM1	Alpha-1-acid glycoprotein 1	2	Chlorpromazine, Thalidomide	-	-
574	TAC3	Tachykinin-3	1	Copper	-	-
575	CHRNB2	Neuronal acetylcholine receptor subunit beta-2	3	Carbamazepine, Nicotine	Atropine	-
576	CACNA1G	Voltage-dependent T-type calcium channel subunit alpha-1G	3	Spironolactone, Flunarizine, Verapamil	-	-
577	BMPR2	Bone morphogenetic protein receptor type-2	1	-	Fostamatinib	-
578	ACVR1B	Activin receptor type-1B	1	-	Fostamatinib	-
579	SERPINA7	Thyroxine-binding globulin	1	Copper	-	-
580	GAPDH	Glyceraldehyde-3-phosphate dehydrogenase	2	Copper, Artenimol	-	-
581	UGDH	UDP-glucose 6-dehydrogenase	1	Copper	-	-
582	CACNA1I	Voltage-dependent T-type calcium channel subunit alpha-1I	3	Spironolactone, Flunarizine, Verapamil	-	-
583	HCAR2	Hydroxycarboxylic acid receptor 2	1	Niacin	-	-
584	RUVBL2	RuvB-like 2	1	Quercetin	-	-
585	ACADSB	Short/branched chain specific acyl-CoA dehydrogenase, mitochondrial	1	Valproic Acid	-	-
586	TF	Serotransferrin	2	Zinc, Copper	-	-
587	HNRNPK	Heterogeneous nuclear ribonucleoprotein K	1	Artenimol	-	-
588	EIF3F	Eukaryotic translation initiation factor 3 subunit F	1	Quercetin	-	-
589	KLK3	Prostate-specific antigen	1	Mifepristone	-	-
590	RANBP1	Ran-specific GTPase-activating protein	1	Copper	-	-
591	QPRT	Nicotinate-nucleotide pyrophosphorylase [carboxylating]	1	Niacin	-	-
592	YWHAB	14-3-3 protein beta/alpha	1	Copper	-	-

593	ACTN1	Alpha-actinin-1	1	Copper	-	-
594	TTLL3	Tubulin monoglycylase TTLL3	1	Theophylline	-	-
595	MPG	DNA-3-methyladenine glycosylase	1	Zinc	-	-
596	MT3	Metallothionein-3	1	Zinc	-	-
597	CHAT	Choline O-acetyltransferase	1	Nicotine	-	-
598	CSNK2B	Casein kinase II subunit beta	1	Quercetin	-	-
599	APOD	Apolipoprotein D	1	Copper	-	-
600	NOMO1	Nodal modulator 1	1	Theophylline	-	-
601	ACTB	Actin, cytoplasmic 1	1	Quercetin	-	-
602	MYLK2	Myosin light chain kinase 2, skeletal/cardiac muscle	1	-	Fostamatinib	-
603	HM13	Minor histocompatibility antigen H13	1	Theophylline	-	-
604	C1QBP	Complement component 1 Q subcomponent-binding protein, mitochondrial	1	Copper	-	-
605	HDAC11	Histone deacetylase 11	1	Panobinostat	-	-
606	STK32A	Serine/threonine-protein kinase 32A	1	-	Fostamatinib	-
607	ALOX15	Arachidonate 15-lipoxygenase	1	Resveratrol	-	-
608	MT2A	Metallothionein-2	1	Zinc	-	-
609	BMP4	Bone morphogenetic protein 4	1	-	-	Calcium
610	LIMK1	LIM domain kinase 1	1	-	Fostamatinib	-
611	AR	Androgen receptor	6	Spironolactone, Tamoxifen, Bicalutamide, Fluphenazine, Dienogest, Enzalutamide	-	-
612	HTT	Huntingtin	1	Copper	-	-
613	NEK11	Serine/threonine-protein kinase Nek11	1	-	Fostamatinib	-
614	SIK1	Serine/threonine-protein kinase SIK1	1	-	Fostamatinib	-
615	TP73	Tumor protein p73	1	Zinc	-	-
616	MAP3K2	Mitogen-activated protein kinase kinase kinase 2	1	-	Fostamatinib	-
617	KCNK2	Potassium channel subfamily K member 2	1	Dronedarone	-	-
618	PRNP	Alternative prion protein	1	Copper	-	-
619	TAOK2	Serine/threonine-protein kinase TAO2	1	-	Fostamatinib	-
620	MAPK9	Mitogen-activated protein kinase 9	1	-	Fostamatinib	-
621	HTR2B	5-hydroxytryptamine receptor 2B	4	Chlorpromazine, Olanzapine, Triflupromazine, Cabergoline	-	-
622	ITGA5	Integrin alpha-5	1	Resveratrol	-	-
623	ASPA	Aspartoacylase	1	Zinc	-	-
624	SPTBN1	Spectrin beta chain, non-erythrocytic 1	1	-	-	Calcium
625	PI4K2B	Phosphatidylinositol 4-kinase type 2-beta	1	Resveratrol	-	-
626	PSPH	Phosphoserine phosphatase	1	Zinc	-	-
627	NEIL2	Endonuclease 8-like 2	1	Copper	-	-
628	STK10	Serine/threonine-protein kinase 10	1	-	Fostamatinib	-
629	UBA1	Ubiquitin-like modifier-activating enzyme 1	1	Quercetin	-	-
630	CKS1B	Cyclin-dependent kinases regulatory subunit 1	1	Fluoxetine	-	-
631	SERPINB9	Serpin B9	1	Raloxifene	-	-
632	EPHB2	Ephrin type-B receptor 2	1	-	Fostamatinib	-
633	TUFM	Elongation factor Tu, mitochondrial	1	Zinc	-	-
634	PSME1	Proteasome activator complex subunit 1	1	Copper	-	-
635	SOAT1	Sterol O-acyltransferase 1	1	Ezetimibe	-	-

636	CHRNA4	Neuronal acetylcholine receptor subunit alpha-4	4	Carbamazepine, Nicotine	Estradiol, Atropine	-
637	NR0B1	Nuclear receptor subfamily 0 group B member 1	1	Dexamethasone	-	-
638	HDGF	Hepatoma-derived growth factor	1	Copper	-	-
639	PRKACB	cAMP-dependent protein kinase catalytic subunit beta	1	-	Fostamatinib	-
640	VIM	Vimentin	1	Artenimol	-	-
641	EIF4A1	Eukaryotic initiation factor 4A-I	1	Copper	-	-
642	MAP3K9	Mitogen-activated protein kinase kinase kinase 9	1	-	Fostamatinib	-
643	IRAK4	Interleukin-1 receptor-associated kinase 4	1	-	Fostamatinib	-
644	EIF6	Eukaryotic translation initiation factor 6	1	Copper	-	-
645	EEF1A1L14	Translation elongation factor 1 alpha 1-like 14	1	Copper	-	-
646	STIP1	Stress-induced-phosphoprotein 1	1	Copper	-	-
647	ANXA4	Annexin A4	1	Copper	-	-
648	RACK1	Receptor of activated protein C kinase 1	1	Copper	-	-
649	SF3A2	Splicing factor 3A subunit 2	1	Copper	-	-
650	HNRNPL	Heterogeneous nuclear ribonucleoprotein L	1	Copper	-	-
651	HNRNPH3	Heterogeneous nuclear ribonucleoprotein H3	1	Copper	-	-
652	HNRNPH1	Heterogeneous nuclear ribonucleoprotein H	1	Copper	-	-
653	NFKB1	Nuclear factor NF-kappa-B p105 subunit	2	Thalidomide, Acetylsalicylic acid	-	-
654	NME1	Nucleoside diphosphate kinase A	2	Zinc, Copper	-	-
655	HSPA13	Heat shock 70 kDa protein 13	1	Copper	-	-
656	SIGMAR1	Sigma non-opioid intracellular receptor 1	2	Noscapine, Nortriptyline	-	-
657	MMP9	Matrix metalloproteinase-9	2	Glucosamine, Zinc	-	-
658	HSPD1	60 kDa heat shock protein, mitochondrial	1	Copper	-	-
659	APOC3	Apolipoprotein C-III	1	Copper	-	-
660	RIPK2	Receptor-interacting serine/threonine-protein kinase 2	1	-	Fostamatinib	-
661	UTRN	Utrophin	1	Zinc	-	-
662	SIVA1	Apoptosis regulatory protein Siva	1	Zinc	-	-
663	FGF19	Fibroblast growth factor 19	1	-	Heparin	-
664	CCS	Copper chaperone for superoxide dismutase	1	Zinc	-	-
665	PCDH19	Protocadherin-19	1	-	-	Calcium
666	MGP	Matrix Gla protein	1	-	-	Calcium
667	ATP1A1	Sodium/potassium-transporting ATPase subunit alpha-1	3	Diazoxide, Ciclopirox, Ouabain	-	-
668	DPYSL2	Dihydropyrimidinase-related protein 2	1	Artenimol	-	-
669	FTO	Alpha-ketoglutarate-dependent dioxygenase FTO	1	Artenimol	-	-
670	DAND5	DAN domain family member 5	1	Zinc	-	-
671	SEPP1	Selenoprotein P	1	Zinc	-	-
672	PRKD1	Serine/threonine-protein kinase D1	1	-	Fostamatinib	-
673	CLEC14A	C-type lectin domain family 14 member A	1	Resveratrol	-	-
674	PLK4	Serine/threonine-protein kinase PLK4	1	-	Fostamatinib	-
675	RYR2	Ryanodine receptor 2	1	Tetracaine	-	-
676	HIST1H2BC	Histone H2B type 1-C/E/F/G/I	1	Copper	-	-

677	IGHM	Ig mu chain C region	1	Zinc	-	-
678	IGHG4	Ig gamma-4 chain C region	1	Copper	-	-
679	ATP1A3	Sodium/potassium-transporting ATPase subunit alpha-3	1	Ouabain	-	-
680	PKN2	Serine/threonine-protein kinase N2	1	-	Fostamatinib	-
681	PIK3C2B	Phosphatidylinositol 4-phosphate 3-kinase C2 domain-containing subunit beta	1	-	Fostamatinib	-
682	PI4KB	Phosphatidylinositol 4-kinase beta	1	-	Fostamatinib	-
683	PHKG1	Phosphorylase b kinase gamma catalytic chain, skeletal muscle/heart isoform	1	-	Fostamatinib	-
684	CDK17	Cyclin-dependent kinase 17	1	-	Fostamatinib	-
685	PSMB10	Proteasome subunit beta type-10	1	Carfilzomib	-	-
686	PSMB9	Proteasome subunit beta type-9	1	Carfilzomib	-	-
687	CAMK2D	Calcium/calmodulin-dependent protein kinase type II subunit delta	1	-	Fostamatinib	-
688	TYK2	Non-receptor tyrosine-protein kinase TYK2	1	-	Fostamatinib	-
689	SH2B3	SH2B adapter protein 3	1	Pazopanib	-	-
690	CLK3	Dual specificity protein kinase CLK3	1	-	Fostamatinib	-
691	SLK	STE20-like serine/threonine-protein kinase	1	-	Fostamatinib	-
692	AOC1	Amiloride-sensitive amine oxidase [copper-containing]	1	-	-	Calcium
693	NOLC1	Nucleolar and coiled-body phosphoprotein 1	1	Doxorubicin	-	-
694	CA14	Carbonic anhydrase 14	1	Acetazolamide	-	-
695	CYP11B1	Cytochrome P450 11B1, mitochondrial	1	Metyrapone	-	-
696	CHRNB3	Neuronal acetylcholine receptor subunit beta-3	1	Nicotine	-	-
697	SCO1	Protein SCO1 homolog, mitochondrial	1	Copper	-	-
698	CHRNA5	Neuronal acetylcholine receptor subunit alpha-5	1	Nicotine	-	-
699	CACNB1	Voltage-dependent L-type calcium channel subunit beta-1	1	Spironolactone	-	-
700	PDE6G	Retinal rod rhodopsin-sensitive cGMP 3',5'-cyclic phosphodiesterase subunit gamma	1	-	Sildenafil	-
701	HEPHL1	Hephaestin-like protein 1	1	Copper	-	-
702	HDAC7	Histone deacetylase 7	1	Panobinostat	-	-
703	INS	Insulin	1	Zinc	-	-
704	NFKBIA	NF-kappa-B inhibitor alpha	1	Acetylsalicylic acid	-	-
705	MELK	Maternal embryonic leucine zipper kinase	1	-	Fostamatinib	-
706	HSPB1	Heat shock protein beta-1	1	Artenimol	-	-
707	CHRNA3	Neuronal acetylcholine receptor subunit alpha-3	2	Fluoxetine, Nicotine	-	-
708	TPI1	Triosephosphate isomerase	2	Zinc, Artenimol	-	-
709	MAP3K1	Mitogen-activated protein kinase kinase kinase 1	1	-	Fostamatinib	-
710	SERPINC1	Antithrombin-III	2	Copper	Heparin	-
711	SRP14	Signal recognition particle 14 kDa protein	1	Artenimol	-	-
712	GPER1	G-protein coupled estrogen receptor 1	2	Genistein	Estradiol	-
713	CHRNB4	Neuronal acetylcholine receptor subunit beta-4	2	Fluoxetine, Nicotine	-	-
714	SCN7A	Sodium channel protein type 7 subunit alpha	1	Valproic Acid	-	-
715	TOP1	DNA topoisomerase 1	2	Topotecan	Irinotecan	-

716	BDNF	Brain-derived neurotrophic factor	1	Copper	-	-
717	CHEK2	Serine/threonine-protein kinase Chk2	1	-	Fostamatinib	-
718	MET	Hepatocyte growth factor receptor	2	Crizotinib	Fostamatinib	-
719	ACAT1	Acetyl-CoA acetyltransferase, mitochondrial	1	Sulfasalazine	-	-
720	KCNJ8	ATP-sensitive inward rectifier potassium channel 8	2	Glyburide, Levosimendan	-	-
721	C8G	Complement component C8 γ chain	1	Zinc	-	-
722	BCL2	Apoptosis regulator Bcl-2	2	Obatoclax	-	Paclitaxel
723	NEIL1	Endonuclease 8-like 1	1	Copper	-	-
724	PTK2	Focal adhesion kinase 1	1	-	Fostamatinib	-
725	MAPKAP	MAP kinase-activated protein kinase 5	1	-	Fostamatinib	-
726	TUBA4A	Tubulin alpha-4A chain	2	Cabazitaxel	-	Vincristine
727	SERPING1	Plasma protease C1 inhibitor	1	Copper	-	-
728	CYP2C8	Cytochrome P450 2C8	1	Isoniazid	-	-
729	CYP1A2	Cytochrome P450 1A2	1	Isoniazid	-	-
730	MAPK4	Mitogen-activated protein kinase 4	1	-	Fostamatinib	-
731	SCN9A	Na+ channel protein type 9 subunit α	2	Valproic Acid, Ranolazine	-	-
732	ABCB11	Bile salt export pump	1	Glyburide	-	-
733	CTSL	Cathepsin L1	1	-	Fostamatinib	-
734	NR3C2	Mineralocorticoid receptor	3	Spironolactone, Felodipine	-	Nimodipine
735	KRT8	Keratin, type II cytoskeletal 8	1	Copper	-	-
736	TKT	Transketolase	1	Copper	-	-
737	FGF2	Fibroblast growth factor 2	2	Sirolimus	Heparin	-
738	CLEC3B	Tetranectin	1	Copper	-	-
739	PRDX2	Peroxiredoxin-2	1	Copper	-	-
740	CFB	Complement factor B	1	Zinc	-	-
741	HIBCH	3-hydroxyisobutyryl-CoA hydrolase, mitochondrial	1	Quercetin	-	-
742	GLRA3	Glycine receptor subunit alpha-3	2	Taurine, Ivermectin	-	-
743	MDH1	Malate dehydrogenase, cytoplasmic	1	Artenimol	-	-
744	FN1	Fibronectin	1	Zinc	-	-
745	ANXA2	Annexin A2	1	Artenimol	-	-
746	AKR1C1	Aldo-keto reductase family 1 member C1	2	Acetylsalicylic acid, Salicylic acid	-	-
747	MAP2	Microtubule-associated protein 2	1	-	-	Paclitaxel
748	SLCO1B1	Solute carrier organic anion transporter family member 1B1	1	-	Clarithromycin	-
749	RXRB	Retinoic acid receptor RXR-beta	2	Bexarotene	-	Rosiglitazone
750	C1S	Complement C1s subcomponent	2	Zinc, Copper	-	-
751	MDM2	E3 ubiquitin-protein ligase Mdm2	1	Zinc	-	-
752	ADRB2	Beta-2 adrenergic receptor	6	Salbutamol, Olanzapine, Nortriptyline, Spermidine, Cabergoline	Propranolol	-
753	PKM	Pyruvate kinase PKM	2	Copper, Artenimol	-	-
754	SMPD1	Sphingomyelin phosphodiesterase	1	Chlorpromazine	-	-
755	POMC	Pro-opiomelanocortin	1	Loperamide	-	-
756	GART	Trifunctional purine biosynthetic protein adenosine-3	1	Pemetrexed	-	-
757	AQP1	Aquaporin-1	1	Acetazolamide	-	-
758	TNK2	Activated CDC42 kinase 1	1	-	Fostamatinib	-
759	ACY1	Aminoacylase-1	1	Copper	-	-
760	CDK15	Cyclin-dependent kinase 15	1	-	Fostamatinib	-
761	VMAT2	Vesicle monoamine transporter type 2	1	-	Fostamatinib	-
762	ANXA1	Annexin A1	1	Dexamethasone	-	-
763	CPT1A	Carnitine O-palmitoyltransferase 1, liver isoform	1	Glyburide	-	-

764	PTAFR	Platelet-activating factor receptor	1	-	Rupatadine	-
765	ESRRB	Steroid hormone receptor ERR2	1	Genistein	-	-
766	SLC6A4	Sodium-dependent serotonin transporter	5	Paroxetine, Fluoxetine, Citalopram, Nortriptyline, Verapamil	-	-
767	FGF4	Fibroblast growth factor 4	1	-	Heparin	-
768	PGRMC1	Membrane-associated progesterone	1	Nortriptyline	-	-
769	HDAC10	Histone deacetylase 10	1	Panobinostat	-	-
770	MT1A	Metallothionein-1A	1	Zinc	-	-
771	CRBN	Protein cereblon	1	Thalidomide	-	-
772	ACVR1	Activin receptor type-1	1	-	Fostamatinib	-
773	HTR5A	5-hydroxytryptamine receptor 5A	1	Olanzapine	-	-
774	PRDX6	Peroxiredoxin-6	1	Copper	-	-
775	ABCC9	ABC transporter subfamily C member 9	1	Glyburide	-	-
776	OGDH	2-oxoglutarate dehydrogenase, mitochondrial	1	Valproic Acid	-	-
777	CYB5R3	NADH-cytochrome b5 reductase 3	1	Copper	-	-
778	CAMK2G	Calcium/calmodulin-dependent protein kinase type II subunit gamma	1	-	Fostamatinib	-
779	IL3	Interleukin-3	1	Zinc	-	-
780	ABCA1	ATP-binding cassette sub-family A member 1	1	Glyburide	-	-
781	AKR1A1	Alcohol dehydrogenase [NADP(+)]	1	Copper	-	-
782	SRC	Proto-oncogene tyrosine-protein kinase Src	4	Ponatinib, Dasatinib, Purvalanol A	Fostamatinib	-
783	FKBP1A	Peptidyl-prolyl cis-trans isomerase FKBP1A	2	Tacrolimus, Sirolimus	-	-
784	DRD3	D(3) dopamine receptor	4	Chlorpromazine, Olanzapine, Pimozide, Cabergoline	-	-
785	CSNK1G3	Casein kinase I isoform gamma-3	1	Purvalanol A	-	-
786	CAMK2A	Calcium/calmodulin-dependent protein kinase type II subunit alpha	1	-	Fostamatinib	-
787	RPL23A	60S ribosomal protein L23a	1	Artenimol	-	-
788	DAPK3	Death-associated protein kinase 3	1	-	Fostamatinib	-
789	NR3C1	Glucocorticoid receptor	4	Dexamethasone, Spironolactone, Mifepristone	Methylprednisolo	-
790	B2M	Beta-2-microglobulin	1	Copper	-	-
791	MAPT	Microtubule-associated protein tau	1	-	-	Paclitaxel
792	SCN4B	Sodium channel subunit beta-4	1	Valproic Acid	-	-
793	SCN3B	Sodium channel subunit beta-3	1	Valproic Acid	-	-
794	SCN2B	Sodium channel subunit beta-2	1	Valproic Acid	-	-
795	CHUK	Inhibitor of nuclear factor kappa-B	1	Sulfasalazine	-	-
796	JAK3	Tyrosine-protein kinase JAK3	1	-	Fostamatinib	-
797	SMO	Smoothened homolog	1	Vismodegib	-	-
798	SCN8A	Na+ channel type 8 subunit alpha	1	Valproic Acid	-	-
799	CDK7	Cyclin-dependent kinase 7	1	Alvocidib	-	-
800	TBXAS1	Thromboxane-A synthase	1	Sulfasalazine	-	-
801	TGFBR2	TGF-beta receptor type-2	1	-	Fostamatinib	-
802	CLK1	Dual specificity protein kinase CLK1	1	-	Fostamatinib	-
803	DAPK1	Death-associated protein kinase 1	1	-	Fostamatinib	-
804	SULT2B1	Sulfotransferase family cytosolic 2B	1	Pregnenolone	-	-
805	SELP	P-selectin	1	-	Heparin	-
806	G6PD	Glucose-6-phosphate 1-dehydrogenase	1	Artenimol	-	-
807	PIK3R1	Phosphatidylinositol 3-kinase regulatory subunit alpha	1	-	Wortmannin	-
808	HBB	Hemoglobin subunit beta	2	Zinc, Copper	-	-
809	PLA2G4A	Cytosolic phospholipase A2	1	-	Quinacrine	-
810	SRD5A2	3-oxo-5-alpha-steroid 4-dehydrogenase 2	1	Spironolactone	-	-
811	POLA1	DNA polymerase alpha catalytic subunit	1	Clofarabine	-	-
812	SHMT2	Serine hydroxymethyltransferase, mitochondrial	1	Artenimol	-	-

813	MAP2K2	Dual specificity mitogen-activated protein kinase kinase 2	1	-	Fostamatinib	-
814	ALDH5A1	Succinate-semialdehyde dehydrogenase, mitochondrial	1	Valproic Acid	-	-
815	POLB	DNA polymerase beta	1	Cytarabine	-	-
816	LGALS1	Galectin-1	1	Artenimol	-	-
817	IDH3A	Isocitrate dehydrogenase [NAD] subunit alpha, mitochondrial	1	Copper	-	-
818	PGR	Progesterone receptor	3	Spironolactone, Mifepristone, Dienogest	-	-
819	SCN10A	Sodium channel protein type 10 subunit alpha	2	Valproic Acid, Levobupivacaine	-	-
820	DRD4	D(4) dopamine receptor	3	Chlorpromazine, Olanzapine, Cabergoline	-	-
821	SCN1B	Sodium channel subunit beta-1	1	Valproic Acid	-	-
822	GLRA2	Glycine receptor subunit alpha-2	1	Taurine	-	-
823	CHRNA6	Neuronal acetylcholine receptor subunit alpha-6	1	Nicotine	-	-
824	ATP6V1A	V-type proton ATPase catalytic subunit A	1	-	Bafilomycin A1	-
825	HGF	Hepatocyte growth factor	1	-	Heparin	-
826	PARP3	Poly [ADP-ribose] polymerase 3	1	Olaparib	-	-
827	KLKB1	Plasma kallikrein	1	Zinc	-	-
828	CMPK1	UMP-CMP kinase	1	Gemcitabine	-	-
829	AHCY	Adenosylhomocysteinase	1	Copper	-	-
830	CA12	Carbonic anhydrase 12	1	Acetazolamide	-	-
831	TUBA3C	Tubulin alpha-3C/D chain	1	Copper	-	-
832	TUBB4A	Tubulin beta-4A chain	1	Artenimol	-	-
833	MGMT	Methylated-DNA--protein-cysteine methyltransferase	1	Zinc	-	-
834	MPO	Myeloperoxidase	1	-	-	Melatonin
835	JUN	Transcription factor AP-1	1	Vinblastine	-	-
836	CALY	Neuron-specific vesicular protein calcyon	1	-	-	Trifluoperazine
837	F13B	Coagulation factor XIII B chain	1	Zinc	-	-
838	SRD5A1	3-oxo-5-alpha-steroid 4-dehydrogenase 1	1	Spironolactone	-	-
839	SLC18A1	Chromaffin granule amine transporter	1	Reserpine	-	-
840	F12	Coagulation factor XII	1	Zinc	-	-
841	SCN11A	Sodium channel protein type 11 subunit alpha	1	Valproic Acid	-	-
842	LDHB	L-lactate dehydrogenase B chain	1	Artenimol	-	-
843	PDE4A	cAMP-specific 3',5'-cyclic phosphodiesterase 4A	2	Theophylline, Pentoxifylline	-	-
844	C1QC	Complement C1q subcomponent subunit C	2	Zinc, Copper	-	-
845	RXRA	Retinoic acid receptor RXR-alpha	2	Bexarotene	-	Rosiglitazone
846	GSK3B	Glycogen synthase kinase-3 beta	2	-	Fostamatinib	Lithium
847	TNF	Tumor necrosis factor	3	Glucosamine, Thalidomide	Chloroquine	-
848	CA7	Carbonic anhydrase 7	1	Acetazolamide	-	-
849	NR1H3	Oxysterols receptor LXR-alpha	1	-	Rhein	-
850	GUCY1A2	Guanylate cyclase soluble subunit alpha-2	1	-	Isosorbide Mononitrate	-
851	CHRNA7	Alpha-7 nicotinic cholinergic receptor subunit	1	Memantine	-	-
852	CYP3A4	Cytochrome P450 3A4	1	Isoniazid	-	-
853	CDK6	Cyclin-dependent kinase 6	1	Alvocidib	-	-
854	PADI4	Protein-arginine deiminase type-4	1	-	Azithromycin	-
855	ROCK1	Rho-associated protein kinase 1	1	Fasudil	-	-
856	NR1H2	Oxysterols receptor LXR-beta	1	-	Rhein	-
857	A2M	Alpha-2-macroglobulin	1	Zinc	-	-
858	DNMT1	DNA (cytosine-5)-methyltransferase 1	1	Procainamide	-	-
859	S100A13	Protein S100-A13	1	-	-	Calcium

860	CYP51A1	Lanosterol 14-alpha demethylase	1	Itraconazole	-	-
861	GOT1	Aspartate aminotransferase, cytoplasmic	1	Copper	-	-
862	BECN1	Beclin-1	1	-	Estradiol	-
863	MT-ATP6	ATP synthase subunit a	1	-	Estradiol	-
864	HPRT1	Hypoxanthine-guanine phosphoribosyltransferase	1	Mercaptopurine	-	-
865	SLC7A11	Cystine/glutamate transporter	1	Sulfasalazine	-	-
866	AKR1B10	Aldo-keto reductase family 1 member B10	1	Sulindac	-	-
867	TYMS	Thymidylate synthase	2	Gemcitabine, Pemetrexed	-	-
868	GABRB3	GABA receptor subunit beta-3	4	Olanzapine, Taurine, Ivermectin, Lamotrigine	-	-
869	GRIN2B	IGluR , NMDA 2B	2	Memantine, Taurine	-	-
870	CHRNA9	Neuronal acetylcholine receptor subunit alpha-9	1	Nicotine	-	-
871	SOD1	Superoxide dismutase [Cu-Zn]	1	Zinc	-	-
872	FAAH	Fatty-acid amide hydrolase 1	1	-	Fostamatinib	-
873	SCN3A	Sodium channel protein type 3 subunit alpha	1	Valproic Acid	-	-
874	TXNRD1	Thioredoxin reductase 1, cytoplasmic	1	Spermidine	-	-
875	SYK	Tyrosine-protein kinase SYK	1	-	Fostamatinib	-
876	GRIA3	Glutamate receptor 3	1	-	-	Lithium
877	ATIC	Bifunctional purine biosynthesis protein	1	Pemetrexed	-	-
878	MAPK3	Mitogen-activated protein kinase 3	1	Sulindac	-	-
879	TGFBR1	TGF-beta receptor type-1	1	-	Fostamatinib	-
880	IFNG	Interferon gamma	1	Glucosamine	-	-
881	HTR1B	5-hydroxytryptamine receptor 1B	3	Olanzapine, Cabergoline	Propranolol	-
882	HTR3A	5-hydroxytryptamine receptor 3A	3	Memantine, Olanzapine, Lamotrigine	-	-
883	CDK5	Cyclin-dependent-like kinase 5	1	Alvocidib	-	-
884	ABCB1	Multidrug resistance protein 1	2	Voacamine	Concanamycin A	-
885	NPR1	Atrial natriuretic peptide receptor 1	1	Nitroprusside	-	-
886	KCNN4	Intermediate conductance calcium-activated potassium channel protein 4	1	-	Clotrimazole	-
887	YARS	Tyrosine--tRNA ligase, cytoplasmic	1	Resveratrol	-	-
888	ATP2C1	Calcium-transporting ATPase type 2C member 1	1	-	-	Calcium
889	GSS	Glutathione synthetase	1	Copper	-	-
890	HSD17B2	Estradiol 17-beta-dehydrogenase 2	1	-	Estradiol	-
891	GSTP1	Glutathione S-transferase P	2	Troglitazone, Curcumin	-	-
892	CHRNA2	Neuronal acetylcholine receptor subunit	2	Fluoxetine, Nicotine	-	-
893	IMPDH1	Inosine-5'-monophosphate	1	Mercaptopurine	-	-
894	ANXA5	Annexin A5	1	Copper	-	-
895	WEE1	Wee1-like protein kinase	1	-	Fostamatinib	-
896	THRA	Thyroid hormone receptor alpha	1	Amiodarone	-	-
897	S100B	Protein S100-B	1	-	-	Calcium
898	ITGB3	Integrin beta-3	1	Resveratrol	-	-
899	CYCS	Cytochrome c	1	Artenimol	-	-
900	GLO1	Lactoylglutathione lyase	1	-	-	Indomethacin
901	EPHX2	Bifunctional epoxide hydrolase 2	1	-	Ebselen	-
902	PRKCB	Protein kinase C beta type	1	Tamoxifen	-	-
903	AVPR2	Vasopressin V2 receptor	1	Tolvaptan	-	-
904	ALAD	Delta-aminolevulinic acid dehydratase	1	Aminolevulinic acid	-	-
905	HTR1A	5-hydroxytryptamine receptor 1A	5	Chlorpromazine, Olanzapine, Nortriptyline, Cabergoline	Propranolol	-
906	CA1	Carbonic anhydrase 1	2	Diazoxide, Acetazolamide	-	-
907	SCN4A	Sodium channel protein type 4 subunit alpha	1	Valproic Acid	-	-
908	KCNJ1	ATP-sensitive inward rectifier potassium channel 1	1	Minoxidil	-	-
909	IMPDH2	Inosine-5'-monophosphate dehydrogenase 2	1	Mercaptopurine	-	-

910	ABAT	4-aminobutyrate aminotransferase, mitochondrial	1	Valproic Acid	-	-
911	AVPR1A	Vasopressin V1a receptor	1	Tolvaptan	-	-
912	PDE4B	cAMP-specific 3',5'-cyclic phosphodiesterase 4B	2	Theophylline, Pentoxifylline	-	-
913	HSP90AA1	Heat shock protein HSP 90-alpha	3	Quercetin, Geldanamycin, Copper	-	-
914	GABRQ	GABA receptor subunit theta	3	Olanzapine, Taurine, Lamotrigine	-	-
915	ITGAL	Integrin alpha-L	1	Simvastatin	-	-
916	KCNA1	Potassium voltage-gated channel subfamily A member 1	1	Nifedipine	-	-
917	SLC12A3	Solute carrier family 12 member 3	1	Diazoxide	-	-
918	GABBR1	GABA type B receptor subunit 1	1	Taurine	-	-
919	NCOA2	Nuclear receptor coactivator 2	2	Genistein	Estradiol	-
920	KCNMA1	Calcium-activated potassium channel subunit alpha-1	1	Diazoxide	-	-
921	COMT	Catechol O-methyltransferase	1	-	-	2-Methoxyestradiol
922	CHRNA10	Neuronal acetylcholine receptor subunit alpha-10	1	Nicotine	-	-
923	GABRP	GABA receptor subunit pi	3	Olanzapine, Taurine, Lamotrigine	-	-
924	GABRE	GABA receptor subunit epsilon	3	Olanzapine, Taurine, Lamotrigine	-	-
925	GABRD	GABA receptor subunit delta	3	Olanzapine, Taurine, Lamotrigine	-	-
926	GABRG3	GABA receptor subunit gamma-3	3	Olanzapine, Taurine, Lamotrigine	-	-
927	PLA2G1B	Phospholipase A2	1	Sulfasalazine	-	-
928	FDPS	Farnesyl pyrophosphate synthase	1	Zoledronic acid	-	-
929	GABRB2	GABA receptor subunit beta-2	3	Olanzapine, Taurine, Lamotrigine	-	-
930	GABRG1	GABA receptor subunit gamma-1	3	Olanzapine, Taurine, Lamotrigine	-	-
931	GABRB1	GABA receptor subunit beta-1	3	Olanzapine, Taurine, Lamotrigine	-	-
932	GGPS1	Geranylgeranyl pyrophosphate synthase	1	Zoledronic acid	-	-
933	CA3	Carbonic anhydrase 3	1	Acetazolamide	-	-
934	CTSS	Cathepsin S	1	-	Fostamatinib	-
935	CFTR	Cystic fibrosis transmembrane conductance regulator	1	Glyburide	-	-
936	HPGDS	Hematopoietic prostaglandin D synthase	1	-	nocodazole	-
937	GSR	Glutathione reductase, mitochondrial	1	Copper	-	-
938	PLG	Plasminogen	1	Copper	-	-
939	ABCC8	ATP-binding cassette sub-family C member 8	1	Glyburide	-	-
940	PDPK1	3-phosphoinositide-dependent protein kinase 1	1	-	Fostamatinib	-
941	EDNRA	Endothelin-1 receptor	1	Acetylsalicylic acid	-	-
942	THRB	Thyroid hormone receptor beta	1	Amiodarone	-	-
943	HTR1D	5-hydroxytryptamine receptor 1D	2	Olanzapine, Cabergoline	-	-
944	CHRNA7	Neuronal acetylcholine receptor subunit alpha-7	2	Acetylcholine, Nicotine	-	-
945	GABRG2	GABA receptor subunit gamma-2	3	Olanzapine, Taurine, Lamotrigine	-	-
946	HSP90AB1	Heat shock protein HSP 90-beta	1	Geldanamycin	-	-
947	HTR1E	5-hydroxytryptamine receptor 1E	1	Olanzapine	-	-
948	FGA	Fibrinogen alpha chain	1	Zinc	-	-
949	AGTR1	Type-1 angiotensin II receptor	1	Telmisartan	-	-
950	PYGL	Glycogen phosphorylase, liver form	1	Alvocidib	-	-
951	GABRA4	GABA receptor subunit alpha-4	3	Olanzapine, Taurine, Lamotrigine	-	-
952	GABRA6	GABA receptor subunit alpha-6	3	Olanzapine, Taurine, Lamotrigine	-	-
953	INSR	Insulin receptor	1	-	Fostamatinib	-
954	CA4	Carbonic anhydrase 4	1	Acetazolamide	-	-
955	TRPV1	Transient receptor potential cation channel subfamily V member 1	1	Capsaicin	-	-

956	PLA2G2A	Phospholipase A2, membrane associated	1	-	-	Indomethacin
957	SLC6A2	Sodium-dependent noradrenaline transporter	3	Paroxetine, Nortriptyline, Maprotiline	-	-
958	MAPK10	Mitogen-activated protein kinase 10	1	-	Fostamatinib	-
959	METAP2	Methionine aminopeptidase 2	1	Nitroxoline	-	-
960	MAPK1	Mitogen-activated protein kinase 1	1	Perifosine	-	-
961	HRH2	Histamine H2 receptor	1	Olanzapine	-	-
962	GABRA5	GABA receptor subunit alpha-5	3	Olanzapine, Taurine, Lamotrigine	-	-
963	PRKACA	cAMP-dependent protein kinase catalytic subunit alpha	2	Fasudil	Fostamatinib	-
964	C1QB	Complement C1q subcomponent subunit B	1	Zinc	-	-
965	ACE	Angiotensin-converting enzyme	1	-	Enalaprilat	-
966	REN	Renin	1	Minoxidil	-	-
967	GABRA3	GABA receptor subunit alpha-3	3	Olanzapine, Taurine, Lamotrigine	-	-
968	GRIN2A	IGluR , NMDA 2A	1	Memantine	-	-
969	VDR	Vitamin D3 receptor	1	Curcumin	-	-
970	NCOA1	Nuclear receptor coactivator 1	1	Genistein	-	-
971	C1R	Complement C1r subcomponent	1	Zinc	-	-
972	GABRA2	GABA receptor subunit alpha-2	3	Olanzapine, Taurine, Lamotrigine	-	-
973	OPRK1	Kappa-type opioid receptor	2	Loperamide, Lamotrigine	-	-
974	SLC18A2	Synaptic vesicular amine transporter	1	Reserpine	-	-
975	GRIN1	IGluR , NMDA 1	1	Memantine	-	-
976	IGHG1	Ig gamma-1 chain C region	1	Copper	-	-
977	DPP4	Dipeptidyl peptidase 4	2	Atorvastatin	-	Sitagliptin
978	MAPK14	Mitogen-activated protein kinase 14	2	Dasatinib	Fostamatinib	-
979	GABRA1	GABA receptor subunit alpha-1	3	Olanzapine, Taurine, Lamotrigine	-	-
980	DHFR	Dihydrofolate reductase	1	Pemetrexed	-	-
981	PKIA	cAMP-dependent protein kinase inhibitor alpha	1	Fasudil	-	-
982	GRIN3A	IGluR , NMDA 3A	1	Memantine	-	-
983	AKR1B1	Aldose reductase	1	Sulindac	-	-
984	CHEK1	Serine/threonine-protein kinase Chk1	1	-	Fostamatinib	-
985	NOS2	Nitric oxide synthase, inducible	1	Dexamethasone	-	-
986	OPRD1	Delta-type opioid receptor	1	Loperamide	-	-
987	F2	Prothrombin	3	Zinc, Copper, Proflavine	-	-
988	PYGM	Glycogen phosphorylase, muscle form	1	Alvocidib	-	-
989	ACHE	Acetylcholinesterase	1	Acetylcholine	-	-
990	F10	Coagulation factor X	1	-	Heparin	-
991	OPRM1	Mu-type opioid receptor	1	Loperamide	-	-
992	CA2	Carbonic anhydrase 2	2	Diazoxide, Acetazolamide	-	-
993	CDK2	Cyclin-dependent kinase 2	1	Alvocidib	-	-
**Predicted Targets**						
1	SLC6A3	Sodium-dependent dopamine transporter	9	Verapamil, Maprotiline, Olanzapine, Lamotrigine, Nortriptyline, Chlorpromazine, Paroxetine, Fluoxetine, Citalopram	-	-
2	HTR3B	5-hydroxytryptamine receptor 3B	1	Olanzapine	-	-
3	HTR1F	5-hydroxytryptamine receptor 1F	3	Olanzapine, Cabergoline, Nortriptyline	-	-
4	GABRR1	GABA receptor subunit rho-1	3	Taurine, Olanzapine, Lamotrigine	-	-
5	GRIK2	IGluR , kainate 2	1	Taurine	-	-
6	GRIA2	Glutamate receptor 2	1	Taurine	-	-

7	TSPO	Translocator protein	3	Lamotrigine, Taurine, Olanzapine	-	-
8	GABRR2	GABA receptor subunit rho-2	3	Taurine, Lamotrigine, Olanzapine	-	-
9	GABRR3	GABA receptor subunit rho-3	3	Olanzapine, Taurine, Lamotrigine	-	-
10	GRIN2C	IGluR , NMDA 2C	2	Taurine, Memantine	-	-
11	GRIN3B	IGluR , NMDA 3B	2	Memantine, Taurine	-	-
12	GRIN2D	IGluR , NMDA 2D	2	Memantine, Taurine	-	-

Genes with elevated mRNA expression levels in the brain (> 5-fold higher than other tissues)

GRIN1	DRD5	GABRB1	CIT	MAPK15
GABRD	GABRA3	GLRA3	DCLK3	MAPK4
HTR1A	GABRG2	GRIN3A	DPYSL2	MAPK9
GABRA6	KCNA1	MAPT	DRD3	MATK
GABRA1	PAK5	PAK3	EPHA4	MTNR1A
CAMK2A	SCN2B	PCDH19	EPHA5	MTNR1B
GABRA5	SCN3B	PDE1B	EPHA6	NEK5
HTR2C	APLP1	RXRG	EPHB6	NTRK2
GRIN2B	CACNA1B	S100B	ESRRG	NTRK3
GLRA2	CAMKK2	SCN8A	FGF4	NUAK1
DRD1	CHAT	TUBB4A	FGFR2	OPRD1
MT3	FGF1	ABCC8	FLT3	OPRK1
PRKCG	GABRQ	ADORA1	GABBR1	PAK1
TTR	GRIN2A	ADRA1B	GABRB3	PDE1A
CACNA1A	HTR5A	ADRA1D	GABRG3	PDE4A
GLRA1	MAST1	ALK	GRIA3	PLCL1
CALY	NIM1K	ASMT	HIPK2	POLB
CHRNA2	OPRM1	ASPA	HSPA2	PRKACB
GABRG1	SCN1A	BDNF	HTR1B	PRKCB
HTR6	SCN1B	CA14	HTR1D	PRKCE
CHRM1	SCN3A	CACNA1G	HTR1E	PRKCZ
CHRNB2	SNCA	CACNA1I	INSRR	PTK2B
DRD2	ACHE	CACNA2D3	KCNH7	RORB
GABRB2	ADORA3	CACNB3	LIMK1	SCN4B
MAP2	CAMK1G	CACNB4	MAP3K10	SLC18A2
NTRK1	CHRM5	CALM1	MAP3K12	SLC7A11
SCN2A	CHRNA4	CAMK2B	MAP3K15	STK32A
ADORA2A	DCLK1	CAMKK1	MAP3K19	THRA
CACNA1E	DCLK2	CDK5	MAP3K9	TLR7
HTR2A	EPHA8	CHRM4	MAP4	TNIK
SBK1	EPHB1	CHRNA6	MAP4K4	TP73
ATP1A2	GABRA2	CHRNA9	MAPK1	TUBA1A
ATP1A3	GABRA4	CHRNB3	MAPK10	TYRO3

Table S3. 368 Predicted ATG interactions and experimental validation.

Predicted interactions between activators and targets						
**No.**	**Drug Name**	**No. Predicted Targets**	**Target Name**	**Gene Name**	**Confidence Score**	**Experimental Validation, Ki (nM)**
**1**	**Acetylcholine**	1	Muscarinic acetylcholine receptor M5	CHRM5	0.8279	800
**2**	**Amiodarone**	1	Beta-2 adrenergic receptor	ADRB2	0.6398	-
			Beta-3 adrenergic receptor	ADRB3	0.6155	-
**3**	**Cabergoline**	6	5-hydroxytryptamine receptor 6	HTR6	0.8499	-
			Beta-3 adrenergic receptor	ADRB3	0.8131	-
			5-hydroxytryptamine receptor 1E	HTR1E	0.7972	-
			5-hydroxytryptamine receptor 3A	HTR3A	0.755	-
			5-hydroxytryptamine receptor 1F	HTR1F	0.6822	-
			Muscarinic acetylcholine receptor M1	CHRM1	0.616	-
**4**	**Citalopram**	24	5-hydroxytryptamine receptor 2A	HTR2A	0.831	-
			Alpha-1D adrenergic receptor	ADRA1D	0.8283	-
			Alpha-1B adrenergic receptor	ADRA1B	0.8265	-
			Muscarinic acetylcholine receptor M2	CHRM2	0.8162	-
			Muscarinic acetylcholine receptor M3	CHRM3	0.8002	-
			D(2) dopamine receptor	DRD2	0.7851	-
			Muscarinic acetylcholine receptor M5	CHRM5	0.7558	-
			Alpha-2A adrenergic receptor	ADRA2A	0.7488	-
			Muscarinic acetylcholine receptor M4	CHRM4	0.7466	-
			Sodium-dependent noradrenaline transporter	SLC6A2	0.7355	-
			Sodium-dependent dopamine transporter	SLC6A3	0.7271	-
			Alpha-2C adrenergic receptor	ADRA2C	0.7199	-
			D(1A) dopamine receptor	DRD1	0.7155	-
			Alpha-2B adrenergic receptor	ADRA2B	0.7071	-
			D(4) dopamine receptor	DRD4	0.6847	-
			5-hydroxytryptamine receptor 1A	HTR1A	0.6829	-
			D(3) dopamine receptor	DRD3	0.6721	-
			D(1B) dopamine receptor	DRD5	0.6638	-
			5-hydroxytryptamine receptor 7	HTR7	0.6596	-
			5-hydroxytryptamine receptor 6	HTR6	0.6445	-
			5-hydroxytryptamine receptor 3A	HTR3A	0.6193	-
			5-hydroxytryptamine receptor 1B	HTR1B	0.616	-
			5-hydroxytryptamine receptor 2B	HTR2B	0.6148	-
			5-hydroxytryptamine receptor 1D	HTR1D	0.6139	-
**5**	**Chlorpromazine**	14	Muscarinic acetylcholine receptor M2	CHRM2	0.9115	150
			Muscarinic acetylcholine receptor M5	CHRM5	0.8418	42
			5-hydroxytryptamine receptor 1B	HTR1B	0.8376	1489
			5-hydroxytryptamine receptor 3A	HTR3A	0.8355	776
			Muscarinic acetylcholine receptor M4	CHRM4	0.8275	-
			5-hydroxytryptamine receptor 1D	HTR1D	0.8053	452
			5-hydroxytryptamine receptor 1E	HTR1E	0.7314	344
			Sodium-dependent serotonin transporter	SLC6A4	0.6925	-
			Beta-1 adrenergic receptor	ADRB1	0.6684	-
			Beta-3 adrenergic receptor	ADRB3	0.641	-
			Histamine H2 receptor	HRH2	0.6398	172
			Beta-2 adrenergic receptor	ADRB2	0.6382	-
			Sodium-dependent noradrenaline transporter	SLC6A2	0.6358	-
			Sodium-dependent dopamine transporter	SLC6A3	0.6344	-
**6**	**Clonidine**	1	5-hydroxytryptamine receptor 2B	HTR2B	0.6078	-
**7**	**Copper**	8	Progesterone receptor	PGR	0.7289	-
			Triosephosphate isomerase	TPI1	0.6895	-
			Plasma kallikrein	KLKB1	0.6581	-
			Desmoplakin	DSP	0.6578	-
			Estrogen receptor	ESR1	0.6329	-
			Phosphoserine phosphatase	PSPH	0.6248	-
			Protein S100-A9	S100A9	0.6066	-
			Glyceraldehyde-3-phosphate dehydrogenase, testis-specific	GAPDHS	0.6051	-
**8**	**Dasatinib**	2	Platelet-derived growth factor receptor alpha	PDGFRA	0.6393	0.47
			Fibroblast growth factor receptor 2	FGFR2	0.6121	1400
**9**	**Diazoxide**	1	Carbonic anhydrase 4	CA4	0.6209	-
**10**	**Doxazosin**	3	Alpha-2A adrenergic receptor	ADRA2A	0.6978	-
			Alpha-2B adrenergic receptor	ADRA2B	0.6642	-
			Alpha-2C adrenergic receptor	ADRA2C	0.6474	-
**11**	**Dronedarone**	7	Beta-2 adrenergic receptor	ADRB2	0.7452	-

			Voltage-dependent T-type calcium channel subunit alpha-1H	CACNA1H	0.7078	-
			Beta-3 adrenergic receptor	ADRB3	0.69	-
			Voltage-dependent T-type calcium channel subunit alpha-1I	CACNA1I	0.6553	-
			D(1B) dopamine receptor	DRD5	0.6428	-
			5-hydroxytryptamine receptor 1B	HTR1B	0.6116	-
			5-hydroxytryptamine receptor 1D	HTR1D	0.6006	-
**12**	**Felodipine**	3	Voltage-dependent L-type calcium channel subunit alpha-1F	CACNA1F	0.644	-
			Voltage-dependent L-type calcium channel subunit beta-1	CACNB1	0.6013	-
			Voltage-dependent P/Q-type calcium channel subunit alpha-1A	CACNA1A	0.6004	-
**13**	**Fluoxetine**	5	5-hydroxytryptamine receptor 2A	HTR2A	0.7219	147.9
			Sodium-dependent noradrenaline transporter	SLC6A2	0.7109	-
			Alpha-2A adrenergic receptor	ADRA2A	0.616	-
			Sodium-dependent dopamine transporter	SLC6A3	0.6147	-
			5-hydroxytryptamine receptor 3A	HTR3A	0.6017	-
**14**	**Fluphenazine**	10	D(1B) dopamine receptor	DRD5	0.7277	12
			5-hydroxytryptamine receptor 2B	HTR2B	0.6877	-
			D(3) dopamine receptor	DRD3	0.6526	0.11
			5-hydroxytryptamine receptor 7	HTR7	0.6422	7.94
			D(4) dopamine receptor	DRD4	0.6339	50
			5-hydroxytryptamine receptor 1A	HTR1A	0.6335	145
			Sodium-dependent noradrenaline transporter	SLC6A2	0.6223	-
			5-hydroxytryptamine receptor 3A	HTR3A	0.6077	-
			Alpha-2B adrenergic receptor	ADRA2B	0.6064	82
			Sodium-dependent serotonin transporter	SLC6A4	0.6062	-
**15**	**Fluspirilene**	3	D(1A) dopamine receptor	DRD1	0.6867	450
			D(3) dopamine receptor	DRD3	0.6085	0.4
			5-hydroxytryptamine receptor 1A	HTR1A	0.604	56
**16**	**Genistein**	1	Estrogen-related receptor gamma	ESRRG	0.6159	-
**17**	**Imatinib**	1	Receptor-type tyrosine-protein kinase FLT3	FLT3	0.6206	6300
**18**	**Isradipine**	4	Voltage-dependent L-type calcium channel subunit alpha-1F	CACNA1F	0.6565	-
			Voltage-dependent L-type calcium channel subunit beta-1	CACNB1	0.6187	-
			Voltage-dependent L-type calcium channel subunit beta-4	CACNB4	0.6092	-
			Voltage-dependent L-type calcium channel subunit beta-3	CACNB3	0.6023	-
**19**	**Ivermectin**	16	GABA receptor subunit gamma-2	GABRG2	0.7215	-
			GABA receptor subunit alpha-1	GABRA1	0.7192	-
			GABA receptor subunit alpha-3	GABRA3	0.6978	-
			GABA receptor subunit beta-2	GABRB2	0.6843	-
			GABA receptor subunit beta-1	GABRB1	0.6817	-
			GABA receptor subunit alpha-5	GABRA5	0.6755	-
			GABA receptor subunit delta	GABRD	0.668	-
			GABA receptor subunit alpha-4	GABRA4	0.6602	-
			GABA receptor subunit theta	GABRQ	0.6591	-
			GABA receptor subunit alpha-2	GABRA2	0.6584	-
			GABA receptor subunit alpha-6	GABRA6	0.6575	-
			GABA receptor subunit gamma-1	GABRG1	0.6476	-
			GABA receptor subunit pi	GABRP	0.6402	-
			Glycine receptor subunit alpha-1	GLRA1	0.6401	-
			GABA receptor subunit gamma-3	GABRG3	0.6314	-
			GABA receptor subunit epsilon	GABRE	0.6189	-
**20**	**Ketanserin**	1	5-hydroxytryptamine receptor 2C	HTR2C	0.6015	-
**21**	**Lacidipine**	3	Voltage-dependent T-type calcium channel subunit alpha-1H	CACNA1H	0.6523	-
			Voltage-dependent T-type calcium channel subunit alpha-1I	CACNA1I	0.6283	-
			Voltage-dependent T-type calcium channel subunit alpha-1G	CACNA1G	0.6049	-
**22**	**Lamotrigine**	29	Alpha-1B adrenergic receptor	ADRA1B	0.9845	-
			Alpha-1D adrenergic receptor	ADRA1D	0.9675	-
			Alpha-2B adrenergic receptor	ADRA2B	0.9361	-
			Alpha-2C adrenergic receptor	ADRA2C	0.9097	-
			5-hydroxytryptamine receptor 2C	HTR2C	0.9066	-
			5-hydroxytryptamine receptor 2B	HTR2B	0.8793	-
			Sodium-dependent noradrenaline transporter	SLC6A2	0.828	-

			5-hydroxytryptamine receptor 6	HTR6	0.8206	-
			D(4) dopamine receptor	DRD4	0.7965	-
			5-hydroxytryptamine receptor 7	HTR7	0.7951	-
			GABA receptor subunit rho-1	GABRR1	0.7867	-
			D(3) dopamine receptor	DRD3	0.7861	-
			Sodium-dependent serotonin transporter	SLC6A4	0.7755	-
			GABA receptor subunit rho-2	GABRR2	0.7751	-
			Sodium-dependent dopamine transporter	SLC6A3	0.7684	-
			GABA receptor subunit rho-3	GABRR3	0.7582	-
			Beta-3 adrenergic receptor	ADRB3	0.7536	-
			Histamine H4 receptor	HRH4	0.7178	-
			Glycine receptor subunit alpha-1	GLRA1	0.7084	-
			Histamine H2 receptor	HRH2	0.7004	-
			5-hydroxytryptamine receptor 1D	HTR1D	0.6976	-
			Sigma non-opioid intracellular receptor 1	SIGMAR1	0.6881	-
			Potassium voltage-gated channel subfamily A member 1	KCNA1	0.6844	-
			Beta-2 adrenergic receptor	ADRB2	0.6746	-
			5-hydroxytryptamine receptor 1E	HTR1E	0.6427	-
			5-hydroxytryptamine receptor 1B	HTR1B	0.6394	-
			5-hydroxytryptamine receptor 1A	HTR1A	0.6387	-
			Translocator protein	TSPO	0.6287	-
			5-hydroxytryptamine receptor 5A	HTR5A	0.6077	-
**23**	**Maprotiline**	14	5-hydroxytryptamine receptor 1A	HTR1A	0.8676	-
			D(1A) dopamine receptor	DRD1	0.8489	402
			5-hydroxytryptamine receptor 2B	HTR2B	0.8308	-
			D(3) dopamine receptor	DRD3	0.8063	504
			Sodium-dependent serotonin transporter	SLC6A4	0.8046	-
			5-hydroxytryptamine receptor 6	HTR6	0.8039	-
			D(1B) dopamine receptor	DRD5	0.7922	429
			5-hydroxytryptamine receptor 1D	HTR1D	0.7913	-
			D(4) dopamine receptor	DRD4	0.791	-
			5-hydroxytryptamine receptor 3A	HTR3A	0.7621	-
			5-hydroxytryptamine receptor 1B	HTR1B	0.7484	-
			Sodium-dependent dopamine transporter	SLC6A3	0.7274	-
			5-hydroxytryptamine receptor 1E	HTR1E	0.7229	-
			Histamine H4 receptor	HRH4	0.6462	-
**24**	**Memantine**	7	IGluR , NMDA 2D	GRIN2D	0.7144	-
			D(3) dopamine receptor	DRD3	0.6879	-
			IGluR , NMDA 3B	GRIN3B	0.6488	-
			IGluR , NMDA 2C	GRIN2C	0.64	-
			Muscarinic acetylcholine receptor M1	CHRM1	0.619	-
			5-hydroxytryptamine receptor 2A	HTR2A	0.6094	-
			D(1A) dopamine receptor	DRD1	0.6082	-
**25**	**Minoxidil**	1	Prostaglandin G/H synthase 2	PTGS2	0.7051	-
**26**	**Nicardipine**	17	Voltage-dependent L-type calcium channel subunit alpha-1S	CACNA1S	0.7513	-
			Voltage-dependent T-type calcium channel subunit alpha-1H	CACNA1H	0.7357	-
			D(2) dopamine receptor	DRD2	0.7162	-
			Alpha-2B adrenergic receptor	ADRA2B	0.6993	-
			Alpha-2C adrenergic receptor	ADRA2C	0.6899	-
			D(1A) dopamine receptor	DRD1	0.6834	-
			Potassium voltage-gated channel subfamily H member 2	KCNH2	0.6751	-
			Histamine H1 receptor	HRH1	0.6717	-
			Alpha-2A adrenergic receptor	ADRA2A	0.658	-
			Voltage-dependent L-type calcium channel subunit alpha-1F	CACNA1F	0.636	-
			D(4) dopamine receptor	DRD4	0.6341	-
			5-hydroxytryptamine receptor 2A	HTR2A	0.6286	-
			5-hydroxytryptamine receptor 6	HTR6	0.6283	-
			D(1B) dopamine receptor	DRD5	0.6242	-
			5-hydroxytryptamine receptor 7	HTR7	0.6187	-
			Beta-2 adrenergic receptor	ADRB2	0.6109	-
			5-hydroxytryptamine receptor 2C	HTR2C	0.6104	-
**27**	**Nifedipine**	6	Voltage-dependent L-type calcium channel subunit beta-1	CACNB1	0.6818	-
			Voltage-dependent L-type calcium channel subunit alpha-1F	CACNA1F	0.6683	-

			Voltage-dependent T-type calcium channel subunit alpha-1G	CACNA1G	0.6479	-
			Voltage-dependent T-type calcium channel subunit alpha-1I	CACNA1I	0.617	-
			Voltage-dependent L-type calcium channel subunit beta-3	CACNB3	0.6112	-
			Voltage-dependent L-type calcium channel subunit beta-4	CACNB4	0.6051	-
**28**	**Nilvadipine**	6	Voltage-dependent L-type calcium channel subunit beta-1	CACNB1	0.6781	-
			Voltage-dependent L-type calcium channel subunit alpha-1F	CACNA1F	0.6658	-
			Voltage-dependent L-type calcium channel subunit beta-3	CACNB3	0.6356	-
			Voltage-dependent T-type calcium channel subunit alpha-1H	CACNA1H	0.6129	-
			Voltage-dependent L-type calcium channel subunit beta-4	CACNB4	0.6108	-
			Voltage-dependent N-type calcium channel subunit alpha-1B	CACNA1B	0.6094	-
**29**	**Nitrendipine**	2	Voltage-dependent L-type calcium channel subunit alpha-1F	CACNA1F	0.6687	-
			Voltage-dependent L-type calcium channel subunit beta-1	CACNB1	0.6672	-
**30**	**Nortriptyline**	14	D(1A) dopamine receptor	DRD1	0.8884	-
			Sodium-dependent dopamine transporter	SLC6A3	0.8835	-
			5-hydroxytryptamine receptor 7	HTR7	0.8492	-
			D(3) dopamine receptor	DRD3	0.8351	-
			D(1B) dopamine receptor	DRD5	0.83	-
			D(4) dopamine receptor	DRD4	0.7977	-
			5-hydroxytryptamine receptor 1B	HTR1B	0.7894	-
			5-hydroxytryptamine receptor 2B	HTR2B	0.7665	-
			5-hydroxytryptamine receptor 3A	HTR3A	0.759	-
			5-hydroxytryptamine receptor 1D	HTR1D	0.7504	-
			Histamine H4 receptor	HRH4	0.7092	6918
			Histamine H2 receptor	HRH2	0.7008	645
			5-hydroxytryptamine receptor 1E	HTR1E	0.699	-
			5-hydroxytryptamine receptor 1F	HTR1F	0.6695	-
**31**	**Olanzapine**	12	Alpha-1D adrenergic receptor	ADRA1D	0.9374	-
			GABA receptor subunit rho-2	GABRR2	0.7549	-
			GABA receptor subunit rho-1	GABRR1	0.7432	-
			GABA receptor subunit rho-3	GABRR3	0.7071	-
			Calmodulin	CALM1	0.6882	-
			Translocator protein	TSPO	0.6871	-
			Potassium voltage-gated channel subfamily A member 1	KCNA1	0.6802	-
			Sodium-dependent serotonin transporter	SLC6A4	0.6781	-
			Sodium-dependent noradrenaline transporter	SLC6A2	0.6748	-
			Sodium-dependent dopamine transporter	SLC6A3	0.6649	-
			5-hydroxytryptamine receptor 3B	HTR3B	0.6325	-
			5-hydroxytryptamine receptor 1F	HTR1F	0.6298	310
**32**	**Paroxetine**	9	5-hydroxytryptamine receptor 2C	HTR2C	0.7465	-
			Histamine H1 receptor	HRH1	0.704	-
			D(2) dopamine receptor	DRD2	0.7031	-
			Sodium-dependent dopamine transporter	SLC6A3	0.6657	-
			5-hydroxytryptamine receptor 7	HTR7	0.6652	-
			5-hydroxytryptamine receptor 2B	HTR2B	0.6502	-
			Alpha-1A adrenergic receptor	ADRA1A	0.6402	-
			5-hydroxytryptamine receptor 1A	HTR1A	0.6191	-
			Alpha-2A adrenergic receptor	ADRA2A	0.6092	-
**33**	**Pimozide**	7	D(1A) dopamine receptor	DRD1	0.7343	4100
			D(1B) dopamine receptor	DRD5	0.6578	-
			D(4) dopamine receptor	DRD4	0.6457	1.8
			Alpha-2C adrenergic receptor	ADRA2C	0.6434	376.5
			5-hydroxytryptamine receptor 2C	HTR2C	0.6197	874
			Alpha-2B adrenergic receptor	ADRA2B	0.6089	821
			5-hydroxytryptamine receptor 1A	HTR1A	0.6044	88
**34**	**Ponatinib**	1	Platelet-derived growth factor receptor beta	PDGFRB	0.7043	-
**35**	**Prazosin**	4	Alpha-2C adrenergic receptor	ADRA2C	0.8713	10.7
			Beta-1 adrenergic receptor	ADRB1	0.7357	-
			Beta-2 adrenergic receptor	ADRB2	0.6915	-
			5-hydroxytryptamine receptor 2A	HTR2A	0.6402	400.76

**36**	**Regorafenib**	2	Receptor-type tyrosine-protein kinase FLT3	FLT3	0.6484	-
			Fibroblast growth factor receptor 3	FGFR3	0.6372	-
**37**	**Rilmenidipine**	3	Alpha-2B adrenergic receptor	ADRA2B	0.7527	-
			Alpha-2C adrenergic receptor	ADRA2C	0.7383	-
			Alpha-1A adrenergic receptor	ADRA1A	0.6467	-
**38**	**Sertindole**	23	Alpha-2A adrenergic receptor	ADRA2A	0.8811	640
			Alpha-2C adrenergic receptor	ADRA2C	0.8499	450
			D(3) dopamine receptor	DRD3	0.8265	2.5
			D(1A) dopamine receptor	DRD1	0.8248	12
			Alpha-2B adrenergic receptor	ADRA2B	0.8143	450
			5-hydroxytryptamine receptor 2B	HTR2B	0.7972	-
			D(1B) dopamine receptor	DRD5	0.7929	-
			5-hydroxytryptamine receptor 7	HTR7	0.7782	28
			D(4) dopamine receptor	DRD4	0.7722	9
			5-hydroxytryptamine receptor 1A	HTR1A	0.7715	280
			Histamine H1 receptor	HRH1	0.7125	130
			5-hydroxytryptamine receptor 1B	HTR1B	0.7029	60
			5-hydroxytryptamine receptor 1D	HTR1D	0.6851	20
			Sodium-dependent serotonin transporter	SLC6A4	0.6791	-
			5-hydroxytryptamine receptor 3A	HTR3A	0.6655	3180
			Muscarinic acetylcholine receptor M3	CHRM3	0.6543	2692
			Muscarinic acetylcholine receptor M1	CHRM1	0.6471	631
			Muscarinic acetylcholine receptor M5	CHRM5	0.6463	-
			Sodium-dependent noradrenaline transporter	SLC6A2	0.6412	-
			5-hydroxytryptamine receptor 1E	HTR1E	0.6407	430
			Muscarinic acetylcholine receptor M2	CHRM2	0.6353	-
			Muscarinic acetylcholine receptor M4	CHRM4	0.6234	-
			Beta-1 adrenergic receptor	ADRB1	0.6164	5000
**39**	**Sorafenib**	2	Platelet-derived growth factor receptor alpha	PDGFRA	0.6289	62
			Fibroblast growth factor receptor 3	FGFR3	0.6166	4200
**40**	**Spironolactone**	1	Estrogen receptor	ESR1	0.6151	-
**41**	**Taurine**	14	GABA receptor subunit rho-1	GABRR1	0.7582	-
			GABA receptor subunit rho-2	GABRR2	0.6823	-
			GABA receptor subunit rho-3	GABRR3	0.6789	-
			Neuronal acetylcholine receptor subunit alpha-7	CHRNA7	0.674	-
			IGluR , NMDA 3A	GRIN3A	0.6705	-
			Glutamate receptor 2	GRIA2	0.6659	-
			Translocator protein	TSPO	0.6574	-
			IGluR , NMDA 2A	GRIN2A	0.6562	-
			IGluR , kainate 2	GRIK2	0.655	-
			IGluR , NMDA 3B	GRIN3B	0.6546	-
			Neuronal acetylcholine receptor subunit alpha-4	CHRNA4	0.6454	-
			IGluR , NMDA 2C	GRIN2C	0.6297	-
			IGluR , NMDA 2D	GRIN2D	0.6281	-
			IGluR , NMDA 1	GRIN1	0.6172	-
**42**	**Thalidomide**	1	Prostaglandin G/H synthase 1	PTGS1	0.6197	-
**43**	**Triflupromazine**	20	Muscarinic acetylcholine receptor M3	CHRM3	0.8613	-
			Muscarinic acetylcholine receptor M4	CHRM4	0.8321	-
			Muscarinic acetylcholine receptor M5	CHRM5	0.8211	-
			D(1B) dopamine receptor	DRD5	0.7602	-
			5-hydroxytryptamine receptor 2A	HTR2A	0.7556	-
			D(4) dopamine receptor	DRD4	0.7379	-
			5-hydroxytryptamine receptor 2C	HTR2C	0.736	-
			D(3) dopamine receptor	DRD3	0.7345	-
			Histamine H1 receptor	HRH1	0.7118	-
			5-hydroxytryptamine receptor 1A	HTR1A	0.7051	-
			Alpha-1A adrenergic receptor	ADRA1A	0.6867	-
			Alpha-1D adrenergic receptor	ADRA1D	0.6791	-
			Alpha-1B adrenergic receptor	ADRA1B	0.6768	-
			5-hydroxytryptamine receptor 7	HTR7	0.6683	-
			Alpha-2B adrenergic receptor	ADRA2B	0.6637	-
			Alpha-2A adrenergic receptor	ADRA2A	0.6522	-
			5-hydroxytryptamine receptor 1D	HTR1D	0.6486	-
			5-hydroxytryptamine receptor 3A	HTR3A	0.6451	-
			Alpha-2C adrenergic receptor	ADRA2C	0.6402	-
			5-hydroxytryptamine receptor 1B	HTR1B	0.6057	-
**44**	**Verapamil**	14	Voltage-dependent T-type calcium channel subunit alpha-1H	CACNA1H	0.825	-
			Sodium-dependent dopamine transporter	SLC6A3	0.7956	-

No.	Drug Name	No. Predicted Targets	Target Name	Gene Name	Confidence Score	Ki (nM)
			Alpha-2A adrenergic receptor	ADRA2A	0.7544	-
			Sodium-dependent noradrenaline transporter	SLC6A2	0.7425	-
			Alpha-2C adrenergic receptor	ADRA2C	0.7335	-
			Alpha-2B adrenergic receptor	ADRA2B	0.6913	-
			Voltage-dependent calcium channel subunit alpha-2/delta-1	CACNA2D1	0.6581	-
			5-hydroxytryptamine receptor 2C	HTR2C	0.6489	-
			5-hydroxytryptamine receptor 2A	HTR2A	0.6419	140
			Histamine H1 receptor	HRH1	0.6348	-
			Muscarinic acetylcholine receptor M2	CHRM2	0.6258	-
			D(1B) dopamine receptor	DRD5	0.6246	-
			D(2) dopamine receptor	DRD2	0.6136	-
			Muscarinic acetylcholine receptor M3	CHRM3	0.6052	-
45	**Zinc**	3	Plasminogen	PLG	0.7173	-
			Progesterone receptor	PGR	0.6077	-
			Apolipoprotein D	APOD	0.6007	-

Predicted interactions between inhibitors and targets

No.	Drug Name	No. Predicted Targets	Target Name	Gene Name	Confidence Score	Ki (nM)
1	**Propranolol**	7	5-hydroxytryptamine receptor 1D	HTR1D	0.7201	4,070
			5-hydroxytryptamine receptor 2C	HTR2C	0.6593	574
			Alpha-2B adrenergic receptor	ADRA2B	0.6491	-
			Alpha-2A adrenergic receptor	ADRA2A	0.6468	-
			Alpha-2C adrenergic receptor	ADRA2C	0.6301	-
			5-hydroxytryptamine receptor 2B	HTR2B	0.6262	-
			Alpha-1B adrenergic receptor	ADRA1B	0.6034	-
2	**Esmolol**	1	Beta-2 adrenergic receptor	ADRB2	0.7053	-
3	**Fostamatinib**	4	Breakpoint cluster region protein	BCR	0.6543	-
			Mitogen-activated protein kinase 11	MAPK11	0.6298	-
			Fibroblast growth factor receptor 4	FGFR4	0.6267	350
			Histamine H1 receptor	HRH1	0.6243	-
4	**Atropine**	1	Neuronal acetylcholine receptor subunit alpha-7	CHRNA7	0.6356	-

Predicted interactions between dual-modulators and targets

No.	Drug Name	No. Predicted Targets	Target Name	Gene Name	Confidence Score	Ki (nM)
1	**Dexmedetomidine**	2	Alpha-2B adrenergic receptor	ADRA2B	0.7197	2.04
			Alpha-2C adrenergic receptor	ADRA2C	0.7018	2.51
2	**Nimodipine**	1	Voltage-dependent T-type calcium channel subunit alpha-1H	CACNA1H	0.6462	-
3	**Thioridazine**	14	Alpha-1D adrenergic receptor	ADRA1D	0.9429	-
			5-hydroxytryptamine receptor 2C	HTR2C	0.7665	46
			D(1B) dopamine receptor	DRD5	0.7547	216
			D(3) dopamine receptor	DRD3	0.7203	1.5
			5-hydroxytryptamine receptor 1A	HTR1A	0.7135	108
			Alpha-2C adrenergic receptor	ADRA2C	0.7115	52.48
			D(4) dopamine receptor	DRD4	0.7015	1.5
			Alpha-2B adrenergic receptor	ADRA2B	0.6908	341.3
			5-hydroxytryptamine receptor 7	HTR7	0.68	99
			Histamine H1 receptor	HRH1	0.6722	16
			Alpha-2A adrenergic receptor	ADRA2A	0.6601	134
			5-hydroxytryptamine receptor 2B	HTR2B	0.6421	-
			Muscarinic acetylcholine receptor M1	CHRM1	0.6273	2.7
			5-hydroxytryptamine receptor 6	HTR6	0.6162	6.3
4	**Trifluoperazine**	10	Alpha-1B adrenergic receptor	ADRA1B	0.8257	-
			Alpha-1D adrenergic receptor	ADRA1D	0.7834	-
			5-hydroxytryptamine receptor 2A	HTR2A	0.7411	5.4
			D(1A) dopamine receptor	DRD1	0.7057	740
			Alpha-2C adrenergic receptor	ADRA2C	0.6919	-
			Alpha-2A adrenergic receptor	ADRA2A	0.6858	-
			Alpha-2B adrenergic receptor	ADRA2B	0.6696	-
			D(1B) dopamine receptor	DRD5	0.6469	-
			D(4) dopamine receptor	DRD4	0.6094	326
			D(3) dopamine receptor	DRD3	0.6003	4.2

Table S4. GO annotation enrichments based on ATG targets.

Cellular Component			
**Rank**	**GO Term**	**No. Targets**	**p-value**
1	nucleus	361	3.10E-22
2	blood microparticle	66	1.92E-11
3	cytosol	425	4.47E-09
4	cytoplasm	396	4.47E-09
5	nucleoplasm	213	1.19E-08
6	extrinsic component of cytoplasmic side of plasma membrane	35	2.20E-08
7	cytoskeleton	41	3.89E-06
8	synapse	48	4.55E-06
9	voltage-gated calcium channel complex	19	1.75E-05
10	voltage-gated sodium channel complex	14	2.80E-05
11	postsynaptic membrane	56	2.80E-05
12	GABA-A receptor complex	16	1.88E-04
13	intracellular ribonucleoprotein complex	12	1.88E-04
14	cytosolic small ribosomal subunit	12	1.89E-04
15	cell junction	68	2.62E-04
16	neuron projection	62	4.27E-04
17	chloride channel complex	21	8.40E-04
18	neuronal postsynaptic density	14	3.51E-03
19	mast cell granule	8	8.19E-03
20	histone deacetylase complex	11	1.24E-02
21	focal adhesion	61	1.39E-02
22	extracellular matrix	30	1.56E-02
23	perinuclear region of cytoplasm	72	1.57E-02
24	spindle microtubule	9	1.57E-02
25	dendrite	57	1.62E-02
26	cornified envelope	7	1.62E-02
27	spherical high-density lipoprotein particle	7	1.68E-02
28	neuronal cell body	56	1.73E-02
29	centrosome	30	1.85E-02
30	microtubule cytoskeleton	18	1.91E-02
31	sarcolemma	18	1.91E-02
32	membrane	182	2.50E-02
33	receptor complex	34	2.59E-02
34	intermediate filament	12	2.73E-02
35	chylomicron	8	2.74E-02
36	nuclear speck	16	3.18E-02
37	inclusion body	6	3.25E-02
38	microtubule	25	3.25E-02
39	very-low-density lipoprotein particle	9	3.53E-02
40	centriole	9	3.53E-02
41	protein complex	43	4.41E-02
42	Z disc	17	4.90E-02
**Molecular Function**			
**Rank**	**GO Term**	**No. Targets**	**p-value**
1	protein serine/threonine kinase activity	183	1.36E-58
2	ATP binding	367	1.98E-54
3	protein tyrosine kinase activity	65	1.83E-22
4	protein kinase activity	89	5.85E-20
5	non-membrane spanning protein tyrosine kinase activity	41	7.54E-17
6	transmembrane receptor protein tyrosine kinase activity	25	1.18E-09
7	RNA binding	54	2.13E-07
8	identical protein binding	96	4.20E-06
9	MAP kinase kinase kinase activity	15	2.54E-05
10	kinase activity	24	3.02E-05
11	protein serine/threonine/tyrosine kinase activity	18	7.81E-05
12	drug binding	42	1.72E-04
13	NAD-dependent histone deacetylase activity (H3-K14 specific)	11	9.53E-04
14	protein kinase C activity	11	9.53E-04
15	inhibitory extracellular ligand-gated ion channel activity	11	1.02E-03
16	voltage-gated sodium channel activity	13	1.06E-03
17	chloride channel activity	19	1.17E-03
18	GABA-A receptor activity	16	1.30E-03
19	cadherin binding	17	1.67E-03
20	serine-type endopeptidase inhibitor activity	22	1.67E-03
21	voltage-gated calcium channel activity	17	1.70E-03
22	cyclin-dependent protein serine/threonine kinase activity	12	1.71E-03
23	chromatin binding	36	1.71E-03
24	steroid hormone receptor activity	24	1.72E-03

25	magnesium ion binding	46	1.74E-03
26	high voltage-gated calcium channel activity	10	1.74E-03
27	enzyme binding	59	2.36E-03
28	calmodulin-dependent protein kinase activity	16	2.78E-03
29	protein kinase binding	51	3.12E-03
30	Rac GTPase binding	11	3.85E-03
31	chaperone binding	14	4.23E-03
32	protein phosphatase binding	17	7.95E-03
33	fibroblast growth factor binding	8	8.52E-03
34	tau-protein kinase activity	8	8.52E-03
35	protein homodimerization activity	91	8.66E-03
36	MAP kinase kinase kinase kinase activity	8	8.75E-03
37	receptor signaling protein tyrosine kinase activity	8	9.00E-03
38	histone deacetylase activity	10	9.04E-03
39	calmodulin binding	24	2.67E-02
40	transcription factor binding	31	2.67E-02
41	RNA polymerase II transcription factor activity, ligand-activated sequence-specific DNA binding	17	2.97E-02
42	DNA binding	45	3.10E-02
43	mRNA binding	11	3.71E-02
44	MAP kinase activity	8	4.40E-02
45	steroid binding	13	4.70E-02
46	microtubule binding	14	4.91E-02
**Biological Process**			
**Rank**	**GO Term**	**No. Targets**	**p-value**
1	protein phosphorylation	182	9.45E-51
2	protein autophosphorylation	117	4.21E-42
3	intracellular signal transduction	99	3.10E-19
4	peptidyl-tyrosine phosphorylation	49	3.92E-16
5	peptidyl-serine phosphorylation	63	3.91E-13
6	peptidyl-tyrosine autophosphorylation	37	3.91E-13
7	transmembrane receptor protein tyrosine kinase signaling pathway	44	1.61E-12
8	peptidyl-threonine phosphorylation	42	1.61E-12
9	regulation of mitotic cell cycle	38	9.69E-12
10	innate immune response	165	1.96E-08
11	signal transduction	159	7.03E-08
12	signal transduction by protein phosphorylation	20	3.80E-07
13	cell differentiation	42	5.62E-07
14	membrane depolarization during action potential	22	6.16E-07
15	activation of protein kinase activity	24	3.96E-06
16	stress-activated protein kinase signaling cascade	20	3.98E-06
17	regulation of apoptotic process	56	4.45E-06
18	cell migration	41	5.10E-06
19	cell cycle	30	1.23E-05
20	axon guidance	93	4.43E-05
21	transcription, DNA-templated	57	1.04E-04
22	visual learning	20	1.08E-04
23	regulation of cell proliferation	44	1.58E-04
24	neurological system process	30	1.82E-04
25	rRNA processing	19	2.35E-04
26	activation of MAPK activity	36	2.58E-04
27	vascular endothelial growth factor receptor signaling pathway	70	2.86E-04
28	positive regulation of phosphatidylinositol 3-kinase signaling	23	2.88E-04
29	positive regulation of protein phosphorylation	36	4.59E-04
30	negative regulation of apoptotic process	87	6.15E-04
31	regulation of membrane potential	41	7.44E-04
32	platelet degranulation	38	7.78E-04
33	apoptotic process	77	1.10E-03
34	activation of MAPKK activity	53	1.45E-03
35	microtubule cytoskeleton organization	15	1.45E-03
36	positive regulation of transcription by RNA polymerase II	11	1.48E-03
37	neurotrophin TRK receptor signaling pathway	76	1.52E-03
38	transcription initiation from RNA polymerase II promoter	50	1.63E-03
39	rhythmic process	19	1.85E-03
40	phosphorylation	16	2.10E-03
41	positive regulation of cell proliferation	75	2.10E-03
42	regulation of transcription, DNA-templated	45	2.11E-03
43	positive regulation of neuron projection development	21	2.54E-03
44	insulin receptor signaling pathway	61	2.74E-03
45	Fc-epsilon receptor signaling pathway	77	2.95E-03
46	regulation of cell motility	10	3.20E-03
47	regulation of ERK1 and ERK2 cascade	10	3.20E-03
48	negative regulation of neuron apoptotic process	35	3.22E-03
49	B cell activation	12	3.37E-03
50	epidermal growth factor receptor signaling pathway	67	3.84E-03

51	neutrophil degranulation	15	4.01E-03
52	wound healing	20	4.42E-03
53	Ras protein signal transduction	54	4.42E-03
54	MAPK cascade	56	5.16E-03
55	positive regulation of protein binding	13	5.76E-03
56	G2/M transition of mitotic cell cycle	25	5.80E-03
57	platelet activation	58	5.80E-03
58	negative regulation of transcription, DNA-templated	37	6.72E-03
59	fibroblast growth factor receptor signaling pathway	63	6.72E-03
60	behavioral response to cocaine	11	6.97E-03
61	establishment of cell polarity	11	6.97E-03
62	positive regulation of telomere capping	9	7.07E-03
63	negative regulation of transcription from RNA polymerase II promoter	50	7.72E-03
64	synaptic transmission, cholinergic	19	7.76E-03
65	positive regulation of ERK1 and ERK2 cascade	43	9.36E-03
66	membrane depolarization	15	9.57E-03
67	activation of JUN kinase activity	15	9.57E-03
68	chloride transmembrane transport	23	9.59E-03
69	phosphatidylinositol-mediated signaling	31	1.32E-02
70	cell proliferation	51	1.32E-02
71	positive regulation of transcription from RNA polymerase II promoter	82	1.38E-02
72	mitotic cell cycle	42	1.38E-02
73	cornification	10	1.54E-02
74	positive regulation of phospholipase C activity	8	1.62E-02
75	adaptive immune response	24	1.62E-02
76	intracellular receptor signaling pathway	15	2.24E-02
77	positive regulation of phosphatidylinositol 3-kinase activity	15	2.24E-02
78	nervous system development	29	2.27E-02
79	toll-like receptor signaling pathway	28	2.31E-02
80	keratinization	11	2.37E-02
81	gamma-aminobutyric acid signaling pathway	16	2.44E-02
82	cardiac muscle contraction	17	2.61E-02
83	positive regulation of peptidyl-serine phosphorylation	21	2.80E-02
84	protein stabilization	19	2.80E-02
85	positive regulation of cell differentiation	9	3.40E-02
86	Rho protein signal transduction	13	3.79E-02
87	regulation of protein binding	7	3.82E-02
88	cellular response to histamine	7	3.82E-02
89	regulation of cell shape	22	4.69E-02
90	positive regulation of apoptotic process	42	4.79E-02
91	cellular response to retinoic acid	16	4.79E-02
92	response to amphetamine	17	4.88E-02
93	SRP-dependent cotranslational protein targeting to membrane	18	4.88E-02
94	response to drug	85	4.96E-02

Table S5. Pathways enriched in targets of autophagy modulators, corresponding targets, and autophagy modulators. Rows highlighted in *blue* are the seven enriched signal transduction pathways in all three categories of modulators, which are also shown in **Figure 5a**.

**294 Pathways Enriched in All Autophagy Modulators**						
**Rank**	**Pathway**	**Class**	**Subclass**	**No. Targets**	**No. Drugs**	**p-value**
1	MAPK signaling pathway	Environmental Information Processing	Signal transduction	108	56	6.91E-18
2	Axon guidance	Organismal Systems	Development	47	20	2.35E-07
3	Ras signaling pathway	Environmental Information Processing	Signal transduction	62	39	5.69E-07
4	Neurotrophin signaling pathway	Organismal Systems	Nervous system	47	26	5.69E-07
5	Regulation of actin cytoskeleton	Cellular Processes	Cell motility	59	42	2.57E-06
6	ErbB signaling pathway	Environmental Information Processing	Signal transduction	35	27	4.08E-06
7	Rap1 signaling pathway	Environmental Information Processing	Signal transduction	56	51	6.57E-06
8	Epstein-Barr virus infection	Human Diseases	Infectious diseases: Viral	52	31	7.13E-06
9	Oxytocin signaling pathway	Organismal Systems	Endocrine system	57	52	8.16E-06
10	Type II diabetes mellitus	Human Diseases	Endocrine and metabolic diseases	25	36	4.94E-05
11	Insulin resistance	Human Diseases	Endocrine and metabolic diseases	39	29	4.94E-05
12	Calcium signaling pathway	Environmental Information Processing	Signal transduction	66	73	4.94E-05
13	Thyroid hormone signaling pathway	Organismal Systems	Endocrine system	40	43	7.47E-05
14	Nicotine addiction	Human Diseases	Substance dependence	26	13	1.19E-04
15	Proteoglycans in cancer	Human Diseases	Cancers: Overview	56	44	2.18E-04
16	Adrenergic signaling in cardiomyocytes	Organismal Systems	Circulatory system	43	58	2.59E-04
17	Viral carcinogenesis	Human Diseases	Cancers: Overview	46	25	3.10E-04
18	GnRH signaling pathway	Organismal Systems	Endocrine system	34	30	3.95E-04
19	Focal adhesion	Cellular Processes	Cellular community - eukaryotes	51	34	9.32E-04
20	Progesterone-mediated oocyte maturation	Organismal Systems	Endocrine system	29	24	1.33E-03
21	mTOR signaling pathway	Environmental Information Processing	Signal transduction	30	25	1.33E-03
22	Serotonergic synapse	Organismal Systems	Nervous system	41	58	1.41E-03
23	Autophagy - animal	Cellular Processes	Transport and catabolism	32	22	1.69E-03
24	Cholinergic synapse	Organismal Systems	Nervous system	38	42	1.69E-03
25	Human immunodeficiency virus 1 infection	0	0	49	29	1.84E-03
26	Hypertrophic cardiomyopathy (HCM)	Human Diseases	Cardiovascular diseases	28	29	1.98E-03
27	Adipocytokine signaling pathway	Organismal Systems	Endocrine system	27	26	2.00E-03
28	IL-17 signaling pathway	Organismal Systems	Immune system	31	23	2.31E-03
29	EGFR tyrosine kinase inhibitor resistance	Human Diseases	Drug resistance: Antineoplastic	31	34	2.31E-03
30	Gastric cancer	Human Diseases	Cancers: Specific types	32	33	2.37E-03
31	Prostate cancer	Human Diseases	Cancers: Specific types	37	45	2.37E-03
32	cAMP signaling pathway	Environmental Information Processing	Signal transduction	62	71	2.44E-03
33	Pathways in cancer	Human Diseases	Cancers: Overview	107	85	2.57E-03
34	Vascular smooth muscle contraction	Organismal Systems	Circulatory system	38	46	2.79E-03
35	Breast cancer	Human Diseases	Cancers: Specific types	31	41	3.30E-03
36	Influenza A	Human Diseases	Infectious diseases: Viral	43	25	4.28E-03
37	PI3K-Akt signaling pathway	Environmental Information Processing	Signal transduction	74	55	4.28E-03
38	Estrogen signaling pathway	Organismal Systems	Endocrine system	36	39	4.32E-03
39	Apelin signaling pathway	Environmental Information Processing	Signal transduction	31	29	4.32E-03
40	Signaling pathways regulating pluripotency of stem cells	Cellular Processes	Cellular community - eukaryotes	27	18	4.36E-03
41	Insulin signaling pathway	Organismal Systems	Endocrine system	36	23	4.41E-03
42	Endocrine resistance	Human Diseases	Drug resistance: Antineoplastic	32	37	4.49E-03
43	Central carbon metabolism in cancer	Human Diseases	Cancers: Overview	30	34	4.60E-03
44	Prion diseases	Human Diseases	Neurodegenerative diseases	17	8	5.39E-03
45	Neuroactive ligand-receptor interaction	Environmental Information Processing	Signaling molecules and interaction	89	63	5.42E-03
46	Circadian entrainment	Organismal Systems	Environmental adaptation	27	28	5.42E-03
47	Alcoholism	Human Diseases	Substance dependence	32	34	5.54E-03
48	MicroRNAs in cancer	Human Diseases	Cancers: Overview	39	48	5.58E-03
49	Non-small cell lung cancer	Human Diseases	Cancers: Specific types	25	22	5.84E-03
50	Glioma	Human Diseases	Cancers: Specific types	25	27	5.84E-03
51	HIF-1 signaling pathway	Environmental Information Processing	Signal transduction	38	34	5.96E-03

52	Hepatitis C	Human Diseases	Infectious diseases: Viral	34	32	5.96E-03
53	Measles	Human Diseases	Infectious diseases: Viral	37	23	6.23E-03
54	Longevity regulating pathway - multiple species	Organismal Systems	Aging	22	23	6.80E-03
55	GABAergic synapse	Organismal Systems	Nervous system	31	26	7.60E-03
56	Fc epsilon RI signaling pathway	Organismal Systems	Immune system	26	20	7.83E-03
57	Complement and coagulation cascades	Organismal Systems	Immune system	34	7	7.91E-03
58	Morphine addiction	Human Diseases	Substance dependence	33	24	8.62E-03
59	Aldosterone synthesis and secretion	Organismal Systems	Endocrine system	29	25	8.62E-03
60	Gastric acid secretion	Organismal Systems	Digestive system	24	24	8.66E-03
61	Human papillomavirus infection	Human Diseases	Infectious diseases: Viral	54	47	9.21E-03
62	Renin secretion	Organismal Systems	Endocrine system	23	38	9.21E-03
63	Gap junction	Cellular Processes	Cellular community - eukaryotes	31	51	9.75E-03
64	T cell receptor signaling pathway	Organismal Systems	Immune system	34	21	1.01E-02
65	FoxO signaling pathway	Environmental Information Processing	Signal transduction	36	20	1.12E-02
66	Arrhythmogenic right ventricular cardiomyopathy (ARVC)	Human Diseases	Cardiovascular diseases	19	21	1.14E-02
67	Melanoma	Human Diseases	Cancers: Specific types	24	25	1.22E-02
68	Inflammatory mediator regulation of TRP channels	Organismal Systems	Sensory system	33	33	1.48E-02
69	Dopaminergic synapse	Organismal Systems	Nervous system	35	41	1.63E-02
70	Adherens junction	Cellular Processes	Cellular community - eukaryotes	20	20	1.65E-02
71	Aldosterone-regulated sodium reabsorption	Organismal Systems	Excretory system	17	14	2.02E-02
72	Ribosome	Genetic Information Processing	Translation	17	2	2.02E-02
73	Taste transduction	Organismal Systems	Sensory system	22	30	2.06E-02
74	Platelet activation	Organismal Systems	Immune system	35	27	2.06E-02
75	Chronic myeloid leukemia	Human Diseases	Cancers: Specific types	25	24	2.18E-02
76	Pertussis	Human Diseases	Infectious diseases: Bacterial	25	16	2.18E-02
77	cGMP-PKG signaling pathway	Environmental Information Processing	Signal transduction	43	61	2.47E-02
78	Acute myeloid leukemia	Human Diseases	Cancers: Specific types	23	33	2.62E-02
79	Herpes simplex infection	Human Diseases	Infectious diseases: Viral	32	17	2.69E-02
80	Chemokine signaling pathway	Organismal Systems	Immune system	36	25	3.03E-02
81	Hepatitis B	Human Diseases	Infectious diseases: Viral	38	29	3.14E-02
82	Toll-like receptor signaling pathway	Organismal Systems	Immune system	30	18	3.23E-02
83	RIG-I-like receptor signaling pathway	Organismal Systems	Immune system	15	9	3.39E-02
84	Tight junction	Cellular Processes	Cellular community - eukaryotes	29	29	3.44E-02
85	C-type lectin receptor signaling pathway	Organismal Systems	Immune system	29	25	3.44E-02
86	Dilated cardiomyopathy (DCM)	Human Diseases	Cardiovascular diseases	23	35	3.48E-02
87	Kaposi sarcoma-associated herpesvirus infection	Human Diseases	Infectious diseases: Viral	43	37	3.55E-02
88	Inositol phosphate metabolism	Metabolism	Carbohydrate metabolism	13	9	3.66E-02
89	Antigen processing and presentation	Organismal Systems	Immune system	13	12	3.66E-02
90	Circadian rhythm	Organismal Systems	Environmental adaptation	8	5	4.02E-02
91	Wnt signaling pathway	Environmental Information Processing	Signal transduction	21	18	4.02E-02
92	VEGF signaling pathway	Environmental Information Processing	Signal transduction	21	24	4.03E-02
93	Fc gamma R-mediated phagocytosis	Organismal Systems	Immune system	23	17	4.50E-02
94	Salivary secretion	Organismal Systems	Digestive system	19	33	4.83E-02
95	Apoptosis	Cellular Processes	Cell growth and death	36	30	5.48E-02
96	Transcriptional misregulation in cancer	Human Diseases	Cancers: Overview	26	34	5.79E-02
97	Regulation of lipolysis in adipocytes	Organismal Systems	Endocrine system	17	31	5.79E-02
98	Shigellosis	Human Diseases	Infectious diseases: Bacterial	20	17	5.95E-02
99	Mitophagy - animal	Cellular Processes	Transport and catabolism	12	13	5.99E-02
100	Prolactin signaling pathway	Organismal Systems	Endocrine system	22	24	6.40E-02
101	Longevity regulating pathway	Organismal Systems	Aging	22	24	6.40E-02
102	Spliceosome	Genetic Information Processing	Transcription	10	4	6.72E-02
103	Necroptosis	Cellular Processes	Cell growth and death	29	21	6.72E-02
104	Sphingolipid signaling pathway	Environmental Information Processing	Signal transduction	31	25	6.79E-02
105	Linoleic acid metabolism	Metabolism	Lipid metabolism	9	5	7.01E-02
106	Epithelial cell signaling in Helicobacter pylori infection	Human Diseases	Infectious diseases: Bacterial	17	16	7.69E-02
107	NF-kappa B signaling pathway	Environmental Information Processing	Signal transduction	22	23	8.18E-02
108	Th17 cell differentiation	Organismal Systems	Immune system	31	31	8.30E-02
109	Bladder cancer	Human Diseases	Cancers: Specific types	16	18	8.37E-02
110	Insulin secretion	Organismal Systems	Endocrine system	24	33	8.39E-02
111	Human cytomegalovirus infection	0	0	46	42	8.58E-02

112	Endocrine and other factor-regulated calcium reabsorption	Organismal Systems	Excretory system	10	17	1.04E-01
113	Toxoplasmosis	Human Diseases	Infectious diseases: Parasitic	30	25	1.11E-01
114	Olfactory transduction	Organismal Systems	Sensory system	9	4	1.11E-01
115	Hippo signaling pathway - multiple species	Environmental Information Processing	Signal transduction	3	1	1.11E-01
116	Long-term potentiation	Organismal Systems	Nervous system	21	24	1.12E-01
117	Small cell lung cancer	Human Diseases	Cancers: Specific types	23	23	1.15E-01
118	Cell cycle	Cellular Processes	Cell growth and death	18	17	1.18E-01
119	Phosphatidylinositol signaling system	Environmental Information Processing	Signal transduction	15	7	1.20E-01
120	Systemic lupus erythematosus	Human Diseases	Immune diseases	15	7	1.20E-01
121	Renal cell carcinoma	Human Diseases	Cancers: Specific types	20	15	1.21E-01
122	Chagas disease (American trypanosomiasis)	Human Diseases	Infectious diseases: Parasitic	32	23	1.26E-01
123	p53 signaling pathway	Cellular Processes	Cell growth and death	14	10	1.31E-01
124	B cell receptor signaling pathway	Organismal Systems	Immune system	19	17	1.31E-01
125	Ribosome biogenesis in eukaryotes	Genetic Information Processing	Translation	6	5	1.31E-01
126	Protein processing in endoplasmic reticulum	Genetic Information Processing	Folding, sorting and degradation	16	11	1.38E-01
127	Melanogenesis	Organismal Systems	Endocrine system	16	15	1.38E-01
128	African trypanosomiasis	Human Diseases	Infectious diseases: Parasitic	10	9	1.41E-01
129	Glucagon signaling pathway	Organismal Systems	Endocrine system	26	17	1.47E-01
130	Choline metabolism in cancer	Human Diseases	Cancers: Overview	22	28	1.47E-01
131	Leukocyte transendothelial migration	Organismal Systems	Immune system	22	16	1.48E-01
132	AMPK signaling pathway	Environmental Information Processing	Signal transduction	23	44	1.59E-01
133	Pancreatic cancer	Human Diseases	Cancers: Specific types	23	24	1.59E-01
134	Amphetamine addiction	Human Diseases	Substance dependence	21	32	1.60E-01
135	Oocyte meiosis	Cellular Processes	Cell growth and death	21	19	1.60E-01
136	Cushing syndrome	Human Diseases	Endocrine and metabolic diseases	27	36	1.61E-01
137	AGE-RAGE signaling pathway in diabetic complications	Human Diseases	Endocrine and metabolic diseases	30	25	1.67E-01
138	Thyroid cancer	Human Diseases	Cancers: Specific types	11	21	1.69E-01
139	Staphylococcus aureus infection	Human Diseases	Infectious diseases: Bacterial	15	5	1.89E-01
140	Tuberculosis	Human Diseases	Infectious diseases: Bacterial	35	26	1.97E-01
141	Osteoclast differentiation	Organismal Systems	Development	33	27	2.02E-01
142	Colorectal cancer	Human Diseases	Cancers: Specific types	22	28	2.09E-01
143	Arachidonic acid metabolism	Metabolism	Lipid metabolism	16	21	2.10E-01
144	Hippo signaling pathway	Environmental Information Processing	Signal transduction	18	14	2.10E-01
145	Relaxin signaling pathway	Organismal Systems	Endocrine system	27	25	2.14E-01
146	Pentose and glucuronate interconversions	Metabolism	Carbohydrate metabolism	5	3	2.18E-01
147	Protein export	Genetic Information Processing	Folding, sorting and degradation	2	3	2.48E-01
148	Salmonella infection	Human Diseases	Infectious diseases: Bacterial	20	14	2.50E-01
149	Hedgehog signaling pathway	Environmental Information Processing	Signal transduction	7	7	2.60E-01
150	Vibrio cholerae infection	Human Diseases	Infectious diseases: Bacterial	7	9	2.60E-01
151	Th1 and Th2 cell differentiation	Organismal Systems	Immune system	21	13	2.66E-01
152	Legionellosis	Human Diseases	Infectious diseases: Bacterial	13	12	2.80E-01
153	Natural killer cell mediated cytotoxicity	Organismal Systems	Immune system	22	16	2.80E-01
154	Fluid shear stress and atherosclerosis	Human Diseases	Cardiovascular diseases	41	30	2.81E-01
155	Endometrial cancer	Human Diseases	Cancers: Specific types	15	18	2.82E-01
156	TNF signaling pathway	Environmental Information Processing	Signal transduction	25	22	2.87E-01
157	PPAR signaling pathway	Organismal Systems	Endocrine system	14	19	3.07E-01
158	ABC transporters	Environmental Information Processing	Membrane transport	7	4	3.49E-01
159	Bacterial invasion of epithelial cells	Human Diseases	Infectious diseases: Bacterial	11	12	3.49E-01
160	Bile secretion	Organismal Systems	Digestive system	16	20	3.58E-01
161	Cellular senescence	Cellular Processes	Cell growth and death	30	31	3.59E-01
162	RNA transport	Genetic Information Processing	Translation	4	4	3.69E-01
163	Amoebiasis	Human Diseases	Infectious diseases: Parasitic	23	16	3.87E-01
164	Steroid hormone biosynthesis	Metabolism	Lipid metabolism	15	14	3.88E-01
165	Long-term depression	Organismal Systems	Nervous system	15	17	3.88E-01
166	Platinum drug resistance	Human Diseases	Drug resistance: Antineoplastic	19	25	4.16E-01
167	NOD-like receptor signaling pathway	Organismal Systems	Immune system	26	20	4.30E-01
168	Ovarian steroidogenesis	Organismal Systems	Endocrine system	12	21	4.34E-01
169	TGF-beta signaling pathway	Environmental Information Processing	Signal transduction	12	8	4.34E-01
170	Proximal tubule bicarbonate reclamation	Organismal Systems	Excretory system	7	5	4.36E-01
171	Apoptosis - multiple species	Cellular Processes	Cell growth and death	7	8	4.36E-01
172	alpha-Linolenic acid metabolism	Metabolism	Lipid metabolism	5	4	4.37E-01

173	Hepatocellular carcinoma	Human Diseases	Cancers: Specific types	29	32	4.61E-01
174	Cocaine addiction	Human Diseases	Substance dependence	14	23	4.85E-01
175	Cholesterol metabolism	Organismal Systems	Digestive system	9	4	5.06E-01
176	Leishmaniasis	Human Diseases	Infectious diseases: Parasitic	19	22	5.17E-01
177	Cytosolic DNA-sensing pathway	Organismal Systems	Immune system	7	4	5.29E-01
178	Carbohydrate digestion and absorption	Organismal Systems	Digestive system	10	21	5.30E-01
179	Amyotrophic lateral sclerosis (ALS)	Human Diseases	Neurodegenerative diseases	14	13	5.47E-01
180	Synaptic vesicle cycle	Organismal Systems	Nervous system	5	5	5.51E-01
181	Basal transcription factors	Genetic Information Processing	Transcription	1	1	5.72E-01
182	Autophagy - other	Cellular Processes	Transport and catabolism	3	7	5.77E-01
183	Antifolate resistance	Human Diseases	Drug resistance: Antineoplastic	10	10	6.08E-01
184	Nitrogen metabolism	Metabolism	Energy metabolism	7	2	6.21E-01
185	Basal cell carcinoma	Human Diseases	Cancers: Specific types	4	6	6.27E-01
186	Terpenoid backbone biosynthesis	Metabolism	Metabolism of terpenoids and polyketides	4	7	6.27E-01
187	Phospholipase D signaling pathway	Environmental Information Processing	Signal transduction	25	31	6.34E-01
188	Proteasome	Genetic Information Processing	Folding, sorting and degradation	8	4	6.37E-01
189	Viral myocarditis	Human Diseases	Cardiovascular diseases	9	11	6.58E-01
190	Cortisol synthesis and secretion	Organismal Systems	Endocrine system	14	20	6.65E-01
191	Malaria	Human Diseases	Infectious diseases: Parasitic	10	11	6.72E-01
192	Base excision repair	Genetic Information Processing	Replication and repair	7	6	7.03E-01
193	Pathogenic Escherichia coli infection	Human Diseases	Infectious diseases: Bacterial	13	18	7.14E-01
194	Collecting duct acid secretion	Organismal Systems	Excretory system	3	6	7.17E-01
195	Retrograde endocannabinoid signaling	Organismal Systems	Nervous system	38	34	7.28E-01
196	Renin-angiotensin system	Organismal Systems	Endocrine system	5	6	7.58E-01
197	Cardiac muscle contraction	Organismal Systems	Circulatory system	18	22	7.58E-01
198	Thyroid hormone synthesis	Organismal Systems	Endocrine system	14	13	7.81E-01
199	Ferroptosis	Cellular Processes	Cell growth and death	8	8	7.92E-01
200	Ether lipid metabolism	Metabolism	Lipid metabolism	4	3	8.46E-01
201	Pentose phosphate pathway	Metabolism	Carbohydrate metabolism	4	3	8.46E-01
202	Ubiquitin mediated proteolysis	Genetic Information Processing	Folding, sorting and degradation	3	3	8.49E-01
203	Parathyroid hormone synthesis, secretion and action	Organismal Systems	Endocrine system	18	22	8.54E-01
204	Primary immunodeficiency	Human Diseases	Immune diseases	5	4	9.40E-01
205	One carbon pool by folate	Metabolism	Metabolism of cofactors and vitamins	5	3	9.40E-01
206	Fructose and mannose metabolism	Metabolism	Carbohydrate metabolism	4	3	9.42E-01
207	Ascorbate and aldarate metabolism	Metabolism	Carbohydrate metabolism	3	3	9.45E-01
208	Vasopressin-regulated water reabsorption	Organismal Systems	Excretory system	3	3	9.45E-01
209	Human T-cell leukemia virus 1 infection	Human Diseases	Infectious diseases: Viral	36	29	9.82E-01
210	Oxidative phosphorylation	Metabolism	Energy metabolism	8	8	1.00E+00
211	Metabolic pathways	Metabolism	Global and overview maps	105	59	1.00E+00
212	Phototransduction	Organismal Systems	Sensory system	1	1	1.00E+00
213	Butanoate metabolism	Metabolism	Carbohydrate metabolism	3	2	1.00E+00
214	Rheumatoid arthritis	Human Diseases	Immune diseases	9	12	1.00E+00
215	Protein digestion and absorption	Organismal Systems	Digestive system	5	6	1.00E+00
216	Pyrimidine metabolism	Metabolism	Nucleotide metabolism	6	7	1.00E+00
217	Alanine, aspartate and glutamate metabolism	Metabolism	Amino acid metabolism	5	5	1.00E+00
218	ECM-receptor interaction	Environmental Information Processing	Signaling molecules and interaction	5	4	1.00E+00
219	Cysteine and methionine metabolism	Metabolism	Amino acid metabolism	7	3	1.00E+00
220	Histidine metabolism	Metabolism	Amino acid metabolism	3	3	1.00E+00
221	Tyrosine metabolism	Metabolism	Amino acid metabolism	2	2	1.00E+00
222	Lysine degradation	Metabolism	Amino acid metabolism	3	3	1.00E+00
223	Fatty acid degradation	Metabolism	Lipid metabolism	6	7	1.00E+00
224	Thiamine metabolism	Metabolism	Metabolism of cofactors and vitamins	1	1	1.00E+00
225	Biosynthesis of amino acids	Metabolism	Global and overview maps	13	3	1.00E+00
226	Sphingolipid metabolism	Metabolism	Lipid metabolism	1	1	1.00E+00
227	Glycerophospholipid metabolism	Metabolism	Lipid metabolism	6	5	1.00E+00
228	Glycerolipid metabolism	Metabolism	Lipid metabolism	4	3	1.00E+00
229	Chemical carcinogenesis	Human Diseases	Cancers: Overview	12	19	1.00E+00
230	Asthma	Human Diseases	Immune diseases	3	5	1.00E+00
231	Thermogenesis	Organismal Systems	Environmental adaptation	33	36	1.00E+00
232	Drug metabolism - cytochrome P450	Metabolism	Xenobiotics biodegradation and metabolism	8	6	1.00E+00
233	Glutamatergic synapse	Organismal Systems	Nervous system	16	22	1.00E+00
234	Fat digestion and absorption	Organismal Systems	Digestive system	5	6	1.00E+00
235	Alzheimer disease	Human Diseases	Neurodegenerative diseases	25	32	1.00E+00
236	Lysosome	Cellular Processes	Transport and catabolism	3	2	1.00E+00
237	Parkinson disease	Human Diseases	Neurodegenerative diseases	17	27	1.00E+00
238	Arginine and proline metabolism	Metabolism	Amino acid metabolism	4	4	1.00E+00

239	Mineral absorption	Organismal Systems	Digestive system	7	6	1.00E+00
240	Cell adhesion molecules (CAMs)	Environmental Information Processing	Signaling molecules and interaction	3	2	1.00E+00
241	Maturity onset diabetes of the young	Human Diseases	Endocrine and metabolic diseases	2	2	1.00E+00
242	RNA degradation	Genetic Information Processing	Folding, sorting and degradation	2	3	1.00E+00
243	DNA replication	Genetic Information Processing	Replication and repair	1	1	1.00E+00
244	Glutathione metabolism	Metabolism	Metabolism of other amino acids	8	10	1.00E+00
245	Glycine, serine and threonine metabolism	Metabolism	Amino acid metabolism	4	3	1.00E+00
246	beta-Alanine metabolism	Metabolism	Metabolism of other amino acids	3	3	1.00E+00
247	Phenylalanine, tyrosine and tryptophan biosynthesis	Metabolism	Amino acid metabolism	1	1	1.00E+00
248	Tryptophan metabolism	Metabolism	Amino acid metabolism	8	9	1.00E+00
249	Phenylalanine metabolism	Metabolism	Amino acid metabolism	1	1	1.00E+00
250	Fatty acid biosynthesis	Metabolism	Lipid metabolism	1	2	1.00E+00
251	Carbon metabolism	Metabolism	Global and overview maps	18	6	1.00E+00
252	Peroxisome	Cellular Processes	Transport and catabolism	6	8	1.00E+00
253	Hematopoietic cell lineage	Organismal Systems	Immune system	8	16	1.00E+00
254	Huntington disease	Human Diseases	Neurodegenerative diseases	16	24	1.00E+00
255	2-Oxocarboxylic acid metabolism	Metabolism	Global and overview maps	3	1	1.00E+00
256	Glyoxylate and dicarboxylate metabolism	Metabolism	Carbohydrate metabolism	3	2	1.00E+00
257	Vitamin digestion and absorption	Organismal Systems	Digestive system	1	2	1.00E+00
258	Pancreatic secretion	Organismal Systems	Digestive system	13	20	1.00E+00
259	JAK-STAT signaling pathway	Environmental Information Processing	Signal transduction	19	29	1.00E+00
260	Galactose metabolism	Metabolism	Carbohydrate metabolism	2	1	1.00E+00
261	Endocytosis	Cellular Processes	Transport and catabolism	13	20	1.00E+00
262	Synthesis and degradation of ketone bodies	Metabolism	Lipid metabolism	1	1	1.00E+00
263	Steroid biosynthesis	Metabolism	Lipid metabolism	3	3	1.00E+00
264	Propanoate metabolism	Metabolism	Carbohydrate metabolism	5	5	1.00E+00
265	Caffeine metabolism	Metabolism	Biosynthesis of other secondary metabolites	1	1	1.00E+00
266	Purine metabolism	Metabolism	Nucleotide metabolism	21	19	1.00E+00
267	Arginine biosynthesis	Metabolism	Amino acid metabolism	3	2	1.00E+00
268	Nucleotide excision repair	Genetic Information Processing	Replication and repair	1	1	1.00E+00
269	Notch signaling pathway	Environmental Information Processing	Signal transduction	2	6	1.00E+00
270	Homologous recombination	Genetic Information Processing	Replication and repair	1	1	1.00E+00
271	Drug metabolism - other enzymes	Metabolism	Xenobiotics biodegradation and metabolism	11	12	1.00E+00
272	Selenocompound metabolism	Metabolism	Metabolism of other amino acids	1	1	1.00E+00
273	Aminoacyl-tRNA biosynthesis	Genetic Information Processing	Translation	1	1	1.00E+00
274	Fatty acid metabolism	Metabolism	Global and overview maps	5	6	1.00E+00
275	Type I diabetes mellitus	Human Diseases	Endocrine and metabolic diseases	4	5	1.00E+00
276	Metabolism of xenobiotics by cytochrome P450	Metabolism	Xenobiotics biodegradation and metabolism	10	13	1.00E+00
277	Non-alcoholic fatty liver disease (NAFLD)	Human Diseases	Endocrine and metabolic diseases	29	25	1.00E+00
278	Pyruvate metabolism	Metabolism	Carbohydrate metabolism	7	4	1.00E+00
279	mRNA surveillance pathway	Genetic Information Processing	Translation	1	1	1.00E+00
280	Amino sugar and nucleotide sugar metabolism	Metabolism	Carbohydrate metabolism	3	2	1.00E+00
281	Biosynthesis of unsaturated fatty acids	Metabolism	Lipid metabolism	1	1	1.00E+00
282	Starch and sucrose metabolism	Metabolism	Carbohydrate metabolism	4	3	1.00E+00
283	Glycolysis / Gluconeogenesis	Metabolism	Carbohydrate metabolism	13	3	1.00E+00
284	Allograft rejection	Human Diseases	Immune diseases	2	3	1.00E+00
285	Inflammatory bowel disease (IBD)	Human Diseases	Immune diseases	4	5	1.00E+00
286	Graft-versus-host disease	Human Diseases	Immune diseases	2	3	1.00E+00
287	Phagosome	Cellular Processes	Transport and catabolism	19	16	1.00E+00
288	Retinol metabolism	Metabolism	Metabolism of cofactors and vitamins	5	3	1.00E+00
289	Folate biosynthesis	Metabolism	Metabolism of cofactors and vitamins	6	8	1.00E+00
290	Nicotinate and nicotinamide metabolism	Metabolism	Metabolism of cofactors and vitamins	3	2	1.00E+00
291	Porphyrin and chlorophyll metabolism	Metabolism	Metabolism of cofactors and vitamins	3	4	1.00E+00
292	Citrate cycle (TCA cycle)	Metabolism	Carbohydrate metabolism	3	3	1.00E+00
293	Valine, leucine and isoleucine degradation	Metabolism	Amino acid metabolism	6	5	1.00E+00
294	Cytokine-cytokine receptor interaction	Environmental Information Processing	Signaling molecules and interaction	13	10	1.00E+00

Supplementary Table S5. Continued.

Pathways Enriched in Targets of Autophagy Activators (p-value < 0.05)

Signal transduction

No.	Pathway	Class	Subclass	Targets	No. Targets	p-value
1	Calcium signaling pathway	Environmental Information Processing	Signal transduction	NOS2; ADCY1; AGTR1; EDNRA; TNNC2; ATP2A1; EGFR; CAMK2D; PDGFRA; CACNA1S; PTGER3; CACNA1F; EDNRB; OXTR; ATP2A2; CAMK4; CACNA1A; ADRB2; PDE1B; CAMK2G; BDKRB2; NOS3; PRKCA; TBXA2R; CACNA1H; CHRM1; HTR7; CD38; CYSLTR1; DRD1; RYR2; HTR6; HTR5A; ITPR2; HTR2C; PDE1A; HRH2; ADORA2A; BDKRB1; ERBB4; PRKACB; GRIN1; ADRB1; ITPR1; GRIN2C; ITPR3; GNAS; SLC25A4; ADRA1D; CXCR4; HTR2B; ITPKA; LHCGR; ADRA1A; PDGFRB; PRKCG; HTR2A; TNNC1; CHRM2; AVPR1B; NOS1; AVPR1A; CHRM3; CHRM5; ADRB3; GRIN2A; GRM5; CACNA1I; PRKCB; PTGFR; VDAC2; TACR1; ERBB2; CACNA1G; DRD5; PRKACA; CACNA1E; ADCY2; RYR1; PTGER1; F2R; CACNA1D; MYLK2; TACR2; ADORA2B; PPIF; CACNA1B; CCKAR; PPP3R1; GRIN2D; ADRA1B; PTK2B; HRH1; HTR4; CACNA1C; CAMK2A; SLC8A1; PDE1C	98	3.30E-08
2	cAMP signaling pathway	Environmental Information Processing	Signal transduction	PDE4B; GRIA2; SSTR2; ADCY1; EDNRA; ROCK2; CAMK2D; GRIN2B; DRD2; CACNA1S; GABBR1; CACNA1F; OXTR; GRIA1; CAMK4; HCAR2; ADRB2; CAMK2G; PPARA; PPP1CC; GRIA3; HCAR3; BDNF; BAD; JUN; RAF1; ACOX1; ATP1A2; SLC9A1; ADCY5; PDE3B; MAPK3; DRD1; SSTR1; RYR2; HTR6; ATP1A3; HTR1B; SSTR5; MAP2K2; RAC1; ATP2A2; MAPK9; ADORA2A; CHRM1; GLP1R; PDE4D; AKT2; PRKACB; GNAI1; BRAF; GRIN3A; ADRB1; GRIN2C; PDE3A; GNAS; CREB1; MAPK1; MAP2K1; GRIN1; CFTR; PTGER2; HTR1D; PIK3CB; CHRM2; NFKBIA; HTR1E; PIK3CD; GRIN2A; PTGER3; MAPK10; ROCK1; AKT1; NPR1; DRD5; MAPK8; PRKACA; TSHR; PDE4A; ADCY2; PIK3CA; ATP1A1; F2R; CACNA1D; GRIA4; PIK3R1; HTR1A; FSHR; NFKB1; HTR1F; GRIN2D; ADORA1; GRIN3B; PDE4C; GABBR2; HTR4; CACNA1C; CAMK2A	98	1.50E-06
3	Apelin signaling pathway	Environmental Information Processing	Signal transduction	SLC9A1; BECN1; ADCY5; GNG2; NOS2; PRKACA; AGTR1; PDE3B; PRKAG3; PRKAA2; ITPR2; MAP2K1; PRKAG1; MAPK3; TGFBR1; RYR2; ADCY2; PRKAG2; RYR1; PRKAB1; MAP2K2; PRKAB2; ADCY1; MYLK2; PRKCE; SERPINE1; CAMK4; AKT2; NOS1; HDAC5; PRKACB; NOS3; GNAI1; PIK3C3; MTOR; PIK3CG; HDAC4; ITPR1; PRKAA1; ITPR3; PLAT; RAF1; SLC8A1; AKT1; RPS6; MAPK1	46	0.000114
4	Rap1 signaling pathway	Environmental Information Processing	Signal transduction	PRKCA; AKT2; CNR1; ADCY5; ITGAL; ADCY1; CSF1R; PDGFD; FLT4; MAP2K1; IGF1R; GNAS; GNAI1; FLT1; PFN1; MAPK3; ADORA2A; FGFR1; VEGFA; MAPK14; ADCY2; EGFR; PIK3CA; EGF; GRIN2B; PDGFRA; DRD2; PDGFRB; MAP2K2; ITGB3; RAC1; F2R; PRKCG; PIK3CB; PFN2; EPHA2; FGF2; HGF; ITGB2; PIK3R1; ADORA2B; MAPK11; KDR; PRKCB; FGFR3; INS; NGF; PRKCI; PIK3CD; INSR; GRIN1; BRAF; FGFR4; GRIN2A; KIT; FGFR2; ACTB; FGF4; ACTG1; PRKCZ; RAF1; SRC; ITGA2B; FGF1; MAPK12; MET; MAPK1; TEK; MAPK13; AKT1	70	0.000595
5	cGMP-PKG signaling pathway	Environmental Information Processing	Signal transduction	PDE5A; ADRA2B; ADORA3; AKT1; ADRA1B; ADCY5; ADCY1; AGTR1; NOS3; PDE3B; EDNRA; KCNMB3; KCNJ8; BAD; GNAI1; MAPK3; ROCK2; ATP2A2; KCNMA1; NPR2; ATP1A3; VDAC2; PIK3CG; ATP1A1; ADRA1A; MAP2K2; CACNA1S; CACNA1D; CACNA1F; MYLK2; EDNRB; ADRA2A; PDE2A; PRKCE; IRS1; OPRD1; ATP2A1; INS; AKT2; ADRB2; ADCY2; KCNMB4; NPR1; KCNMB2; ADRA2C; INSR; ADRB3; ADORA1; ITPR2; ADRB1; ITPR1; PPP3R1; PDE3A; GUCY1A2; CACNA1C; ITPR3; PPIF; BDKRB2; CREB1; RAF1; SLC8A1; MAP2K1; ROCK1; ADRA1D; PPP1CC; ATP1A2; SLC25A4; MAPK1	68	0.000979
6	PI3K-Akt signaling pathway	Environmental Information Processing	Signal transduction	ITGA4; PPP2R5C; IKBKB; CDK2; ITGAV; FGFR1; CHUK; EGFR; PDGFRA; TLR2; KIT; YWHAB; HSP90AB1; EGF; JAK1; NOS3; ITGA5; EIF4E; BDNF; FGF4; JAK2; IFNAR2; RAF1; ITGA2B; FN1; VWF; PRKCA; TP53; GNG2; FGFR3; PRKAA2; FLT4; IGF1R; BAD; FLT1; MAPK3; PPP2R2A; NGF; MAP2K2; ITGB3; RAC1; GSK3B; PDGFD; CHRM1; KDR; ERBB4; AKT2; MTOR; MDM2; IL3; CREB1; BCL2; FGF1; MET; TEK; MAPK1; VEGFA; EPOR; CSF1R; MAP2K1; CDK6; PDPK1; PIK3CA; IFNAR1; PDGFRB; PIK3CB; CHRM2; NTRK2; IRS1; RXRA; INS; YWHAH; PIK3CD; PPP2CB; HSP90AA1; TLR4; IL2RA; FGFR4; PCK1; SYK; CDK4; ERBB2; RPS6; AKT1; IL2RB; IL6; JAK3; HGF; PIK3CG; CD19; PTK2; IL2; F2R; EPHA2; PIK3R1; IL2RG; NTRK1; PPP2CA; NFKB1; INSR; HSP90B1; CSF3R; FGFR2; GHR; FLT3; PRKAA1; VTN; FGF2	108	0.004234
7	AMPK signaling pathway	Environmental Information Processing	Signal transduction	PPP2R5C; PPP2CA; PFKFB1; PRKAB1; PRKAA2; IGF1R; PRKAG1; CFTR; EEF2; PRKAG2; PIK3CA; FBP1; PDPK1; PPP2R2A; ADRA1A; PRKAG3; PRKAB2; PIK3CB; PIK3R1; HMGCR; IRS1; INS; AKT2; PIK3CD; PPP2CB; INSR; CCNA2; SIRT1; MTOR; ULK1; PPARG; SREBF1; PRKAA1; CREB1; PCK1; CPT1A; ACACB; HNF4A; AKT1	39	0.009535

8	MAPK signaling pathway	Environmental Information Processing	Signal transduction	TGFBR1; IKBKB; FGFR1; CHUK; EGFR; MAP2K5; PDGFRA; RAC1; CACNA1S; CACNA1F; MAPK11; BDNF; CACNA1A; KIT; CACNB4; EGF; FGF4; CACNA2D3; JUN; CACNA2D2; RAF1; MAPK12; PRKCA; TP53; CACNA1H; PLA2G4A; FGFR3; MAPKAPK2; FLT4; IGF1R; FLT1; MAPK3; NGF; MAP2K2; TGFB1; CACNG1; CACNG2; MAPK9; PDGFD; MAP3K12; NFKB1; KDR; ERBB4; AKT2; PRKACB; BRAF; MAP3K1; CACNA2D1; MAP3K2; FGF1; MET; TEK; MAPK1; HSPB1; VEGFA; HSPA8; CSF1R; MAP2K1; IRAK4; RPS6KA1; ZAK; PDGFRB; PRKCG; IL1R1; NTRK2; NFKB2; INS; CACNB3; CACNB1; FGFR4; TNF; CACNA1I; PRKCB; HSPA2; MAPK10; CASP3; ERBB2; MAPK13; CACNA1G; AKT1; MAPK8; PRKACA; CACNA1E; MAPK8IP1; HGF; TGFBR2; MAPK14; CACNB2; FLNA; CACNA1D; EPHA2; MAPT; NTRK1; RPS6KA3; CACNA1B; PPP3R1; INSR; FGFR2; IL1B; FLT3; CACNA1C; TAOK2; FGF2; PPP5C	104	0.0139
9	Ras signaling pathway	Environmental Information Processing	Signal transduction	PRKCA; AKT2; VEGFA; GNG2; IKBKB; HTR7; MAPK9; PLA2G4A; CSF1R; FLT4; MAP2K1; IGF1R; FLT1; PLA2G2A; MAPK3; FGFR1; NTRK1; CHUK; EGFR; PIK3CA; EGF; RAB5A; GRIN2B; PDGFRA; PLA2G1B; PDGFRB; MAP2K2; RAC1; ABL1; PRKCG; PIK3CB; PRKACA; EPHA2; PDGFD; HGF; INS; PIK3R1; NTRK2; ABL2; KDR; BDNF; FGFR3; NFKB1; NGF; PAK5; PIK3CD; PRKACB; INSR; MAPK8; GRIN1; FGFR4; GRIN2A; KIT; PTPN11; FGFR2; PRKCB; FLT3; PLA2G2E; FGF4; MAPK10; BAD; ZAP70; RAF1; FGF2; FGF1; MET; MAPK1; TEK; AKT1	69	0.018261
10	VEGF signaling pathway	Environmental Information Processing	Signal transduction	PRKCA; HSPB1; VEGFA; PLA2G4A; MAPKAPK2; MAP2K1; MAPK3; MAPK14; PIK3CA; PTK2; MAP2K2; RAC1; PRKCG; PIK3CB; PIK3R1; MAPK11; KDR; AKT2; PIK3CD; PPP3R1; NOS3; PRKCB; PTGS2; BAD; RAF1; SRC; MAPK12; AKT1; MAPK13; MAPK1	30	0.029696
11	Sphingolipid signaling pathway	Environmental Information Processing	Signal transduction	PRKCA; TP53; MAPK8; PPP2R5C; PPP2CA; ADORA3; CTSD; MAP2K1; ABCC1; MAPK3; ROCK2; MAPK14; PIK3CA; PRKCZ; PPP2R2A; FYN; MAP2K2; RAC1; PRKCG; PIK3CB; PIK3R1; BCL2; MAPK11; OPRD1; MAPK9; NFKB1; AKT2; PIK3CD; PPP2CB; BDKRB2; NOS3; GNAI1; TNF; ADORA1; PRKCB; FCER1A; S1PR5; MAPK10; PDPK1; PRKCE; RAF1; SMPD1; MAPK12; ROCK1; AKT1; MAPK13; MAPK1	47	0.030014
**Human diseases**						
**Cancers: overview**						
1	Proteoglycans in cancer	Human Diseases	Cancers: Overview	IL12B; PRKCA; TP53; SLC9A1; TLR4; VEGFA; PRKACA; PLAU; ITPR2; MAP2K1; IGF1R; CTSL; MAPK3; ROCK2; FGFR1; KDR; MAPK14; EGFR; FLNA; CAMK2D; PDPK1; PTK2; RAC1; ITGA5; MAP2K2; ITGB3; TGFB1; PRKCG; PIK3CB; TLR2; FGF2; HGF; PIK3R1; CAMK2A; ITGAV; MAPK11; MMP9; SMO; PTPN11; PRKCB; ERBB4; IQGAP1; AKT2; ESR1; CAMK2G; PRKACB; PPP1CC; MAPK13; DDX5; BRAF; PIK3CD; TNF; MTOR; PIK3CA; MDM2; ACTB; ITPR1; HIF1A; LUM; MMP2; ITPR3; CASP3; ACTG1; VTN; PLAUR; RAF1; SRC; ROCK1; MAPK12; MET; AKT1; ERBB2; FN1; RPS6; MAPK1	75	0.00118
2	Pathways in cancer	Human Diseases	Cancers: Overview	TGFBR1; NOS2; ADCY1; AGTR1; CDK2; EDNRA; HMOX1; ITGAV; ROCK2; FGFR1; CHUK; EGFR; CAMK2D; PDGFRA; RAC1; MTOR; IKBKB; IL13; ABL1; BIRC5; EDNRB; HSP90AB1; SKP1; SMO; JAK1; CXCR4; AR; PRKACB; CAMK2G; BDKRB2; KIT; IFNGR2; LEF1; EGF; CASP8; FGF4; JAK2; IFNAR2; JUN; GSTA1; RAF1; GSTA3; ITGA2B; NCOA1; FN1; PRKCA; TP53; POLK; ADCY5; BCR; FGFR3; CDK6; IGF1R; BAD; MAPK3; RXRG; RET; MAP2K2; TGFB1; GSK3B; RAD51; BDKRB1; MAPK9; AKT2; ESR1; STAT5B; RARB; GNAI1; BRAF; MDM2; PPARG; IL3; RXRB; GNAS; RARA; GSTM4; BCL2; FGF1; MET; MAPK1; IL12B; VEGFA; EPOR; CSF1R; KLK3; CKS1B; MAP2K1; FLT4; PTGER2; GNG2; IFNAR1; PDGFRB; PRKCG; PIK3CB; HDAC2; GSTA2; MMP9; NQO1; ALK; RXRA; NFKB2; PIK3CD; SLC2A1; HSP90AA1; IL2RA; FGFR4; PTGER3; ESR2; PRKCB; PTGS2; GSTP1; MAPK10; CASP3; PIM1; CDK4; ROCK1; ERBB2; DAPK1; AKT1; TERT; IL2RB; IL6; MAPK8; PRKACA; JAK3; CYCS; HGF; IL5; HDAC1; ADCY2; PIK3CA; PTGER1; PTK2; IL2; F2R; TGFBR2; PIK3R1; IL2RG; NTRK1; NFKB1; CXCL8; CASP7; GSTT2; HSP90B1; CSF3R; FGFR2; HIF1A; DAPK3; FLT3; MMP2; CAMK2A; IFNG; NFKBIA; MMP1; FGF2; PPARD; JUP; TXNRD1; CSF2RA	159	0.00158
3	Transcriptional misregulation in cancer	Human Diseases	Cancers: Overview	TP53; IL6; POLK; CSF1R; IL2RB; PLAT; GRIA3; PLAU; IGF1R; FCGR1A; FLT1; RXRG; HDAC1; CD40; CDK9; PTK2; CEBPB; MMP3; HDAC2; MMP9; NTRK1; RXRA; ATM; NFKB1; CXCL8; DDX5; MPO; MDM2; PPARG; IGFBP3; FLT3; RXRB; NR4A3; TGFBR2; ELANE; JUP; MET; IL3; RARA	39	0.023843
**Cancers: specific types**						
1	Prostate cancer	Human Diseases	Cancers: Specific types	TP53; IKBKB; NFKBIA; CDK2; MAP2K1; IGF1R; PLAU; MAPK3; FGFR1; MMP3; CHUK; EGFR; PIK3CA; PDPK1; PDGFRA; PDGFRB; MAP2K2; GSK3B; PIK3CB; PDGFD; INS; PIK3R1; MDM2; MMP9; HSP90AB1; NFKB1; AR; AKT2; PIK3CD;	47	0.030014

				HSP90AA1; BRAF; KLK3; HSP90B1; MTOR; FGFR2; EGF; LEF1; SRD5A2; GSTP1; PLAT; BAD; CREB1; RAF1; BCL2; AKT1; ERBB2; MAPK1		
2	Non-small cell lung cancer	Human Diseases	Cancers: Specific types	PRKCA; TP53; POLK; MAP2K1; CDK6; MAPK3; RXRG; EGFR; PIK3CA; EGF; PDPK1; FHIT; MAP2K2; PRKCG; PIK3CB; JAK3; PIK3R1; ALK; RXRA; AKT2; PIK3CD; STAT5B; RARB; BRAF; PRKCB; RXRB; BAD; RAF1; CDK4; AKT1; ERBB2; MAPK1	32	0.030439
**Cardiovascular diseases**						
1	Hypertrophic cardiomyopathy (HCM)	Human Diseases	Cardiovascular diseases	ITGA4; IL6; PRKAB1; PRKAA2; PRKAG1; CACNB2; RYR2; ITGAV; PRKAG2; ACE; TGFB1; PRKAG3; ITGA5; ITGB3; CACNA1S; CACNG1; CACNA1D; CACNA1F; CACNG2; TNNC1; ATP2A2; CACNB3; CACNB1; TPM1; PRKAB2; TNF; CACNB4; ACTB; CACNA1C; ACTG1; CACNA2D3; CACNA2D2; CACNA2D1; SLC8A1; ITGA2B; PRKAA1	36	0.007119
2	Arrhythmogenic right ventricular cardiomyopathy (ARVC)	Human Diseases	Cardiovascular diseases	ITGA4; DSP; ITGAV; RYR2; CACNB2; ITGB3; CACNA1S; CACNG1; CACNG2; CACNA1F; CACNA1D; ATP2A2; CACNB3; CACNB1; ITGA5; CACNB4; ACTB; LEF1; CACNA1C; ACTG1; CACNA2D3; CACNA2D2; CACNA2D1; SLC8A1; ITGA2B; JUP	26	0.009535
3	Dilated cardiomyopathy (DCM)	Human Diseases	Cardiovascular diseases	ITGA4; ADCY5; PRKACA; GNAS; ITGAV; RYR2; ADCY2; CACNB2; TGFB1; ITGA5; ITGB3; CACNA1S; CACNG1; CACNA1D; CACNA1F; ADCY1; CACNG2; TNNC1; ATP2A2; CACNB3; CACNB1; TPM1; PRKACB; TNF; CACNB4; ADRB1; ACTB; CACNA1C; ACTG1; CACNA2D3; CACNA2D2; CACNA2D1; SLC8A1; ITGA2B	34	0.011776
**Endocrine and metabolic diseases**						
1	Insulin resistance	Human Diseases	Endocrine and metabolic diseases	AKT2; IL6; MAPK8; IKBKB; PRKAG1; NFKBIA; NR1H2; PRKAG3; PRKAA2; PRKCQ; NR1H3; PYGB; RPS6KA1; PRKAG2; PIK3CA; PRKCZ; PYGM; PRKAB1; PRKCD; TNF; PRKAB2; PTPN1; PIK3CB; INS; PIK3R1; PRKCE; IRS1; PYGL; RPS6KA3; MAPK9; NFKB1; SLC2A2; PIK3CD; SLC2A1; PPARA; PPP1CC; INSR; PTPN11; MTOR; PRKCB; SREBF1; PRKAA1; MAPK10; PDPK1; CREB1; GSK3B; PCK1; CPT1A; NOS3; ACACB; AKT1	51	4.80E-05
2	Type II diabetes mellitus	Human Diseases	Endocrine and metabolic diseases	MAPK8; IKBKB; GCK; CACNA1E; MAPK3; ABCC8; PIK3CA; KCNJ11; PRKCZ; TNF; CACNA1D; PIK3CB; INS; PIK3R1; PRKCE; IRS1; MAPK9; CACNA1B; CACNA1A; SLC2A2; PIK3CD; PRKCD; INSR; MTOR; CACNA1C; MAPK10; PKM; CACNA1G; MAPK1	29	0.001462
3	Cushing syndrome	Human Diseases	Endocrine and metabolic diseases	NR5A1; POMC; CAMK2A; CACNA1H; ADCY5; PRKACA; AGTR1; CDK2; AHR; MAP2K1; CDK6; MAPK3; ADCY2; EGFR; ITPR2; CAMK2D; HSD3B1; CYP21A2; MAP2K2; CACNA1S; PDE8A; CACNA1D; CACNA1F; ADCY1; KCNK2; PRKACB; CAMK2G; CYP11B1; PDE8B; GNAI1; BRAF; CACNA1I; ITPR1; LEF1; PDE11A; CACNA1C; ITPR3; GNAS; CREB1; GSK3B; CDK4; CYP17A1; CACNA1G; MAPK1	44	0.027378
**Infectious diseases**						
1	Pertussis	Human Diseases	Infectious diseases: Bacterial	IL12B; LY96; IL6; MAPK8; NOS2; IRAK4; MAPK3; MAPK14; C1S; C4BPA; C1R; ITGA5; C4BPB; C5; ITGB2; CASP1; MAPK11; CFL1; C1QC; C1QB; MAPK9; NFKB1; CXCL8; CASP7; TLR4; GNAI1; TNF; IL1B; C3; MAPK10; SERPING1; CASP3; JUN; MAPK12; MAPK13; C1QA; MAPK1	37	0.005206
2	Chagas disease (American trypanosomiasis)	Human Diseases	Infectious diseases: Parasitic	IL12B; IL6; BDKRB2; NOS2; ADCY1; NFKBIA; IRAK4; SERPINE1; MAPK3; TGFBR2; MAPK14; CCL2; CHUK; PIK3CA; ACE; PPP2R2A; TGFBR1; TLR2; CD3E; IKBKB; TGFB1; PIK3CB; CALR; TLR9; PIK3R1; MAPK11; C1QC; C1QB; IL2; MAPK9; NFKB1; AKT2; PIK3CD; PPP2CB; TLR4; PPP2CA; MAPK8; GNAI1; CXCL8; TNF; IFNGR2; CASP8; IL1B; C3; MAPK10; IFNG; GNAS; JUN; MAPK12; MAPK1; MAPK13; C1QA; AKT1	53	0.009309
3	Epithelial cell signaling in Helicobacter pylori infection	Human Diseases	Infectious diseases: Bacterial	MAPK8; IKBKB; ATP6V1A; NFKBIA; CXCR1; MAPK14; CHUK; EGFR; ADAM17; ADAM10; LYN; RAC1; ATP6V1B2; MAPK11; MAPK9; NFKB1; CXCL8; PTPN11; MAPK10; CASP3; CSK; JUN; SRC; MAPK12; MET; MAPK13	26	0.018252
4	Legionellosis	Human Diseases	Infectious diseases: Bacterial	IL12B; IL6; HSPA8; NFKBIA; CYCS; TLR2; HSPD1; ITGB2; CASP1; NFKB2; NFKB1; ARF1; CXCL8; CASP7; TLR4; TNF; EEF1A1; CASP8; IL1B; HSPA2; C3; CASP3; CLK1	23	0.022572
5	Vibrio cholerae infection	Human Diseases	Infectious diseases: Bacterial	KCNQ1; ACTB; ARF1; SLC12A2; PRKCA; PRKACA; ATP6V1A; ATP6V1B2; ACTG1; GNAS; PRKACB; CFTR	12	0.030114
6	Measles	Human Diseases	Infectious diseases: Viral	IL12B; TP53; IL6; HSPA8; IL13; JAK2; NFKBIA; IL2RB; CDK2; PRKCQ; JAK3; CDK6; CSNK2A1; PIK3CA; AKT2; IFNAR1; FYN; TP73; CD3E; IL2; TLR2; IFNGR2; PIK3CB; TLR9; PIK3R1; IL2RG; FCGR2B; JAK1; NFKB1; CHUK; PIK3CD; STAT5B; TLR4; CSNK2B; IL2RA; IRAK4; IL1B; RACK1; TYK2; TLR7; HSPA2; CSNK2A2; IFNG; IFNAR2; TACR1; GSK3B; CDK4; TNFRSF10B; AKT1	49	0.040024

**Neural diseases**						
1	Morphine addiction	Human Diseases	Substance dependence	PDE4B; PRKCA; GABRD; KCNJ5; OPRM1; PDE7B; GNG2; PRKACA; PDE3B; PDE4A; GABRQ; PDE11A; GABRE; GABRA3; GABRR1; PDE2A; GABRR3; DRD1; GABRB2; ADCY2; GABRG1; KCNJ9; ADCY5; GABRP; GABBR1; PRKCG; GABRA2; PDE1A; GABRG3; PDE7A; PDE10A; GABRA1; PDE4D; CACNA1B; CACNA1A; ADRBK2; PDE1B; GABRA5; ADCY1; PRKACB; PDE8B; GNAI1; GABRB1; ADORA1; PRKCB; PDE4C; KCNJ3; GABRA4; GABBR2; GABRA6; PDE3A; GABRB3; GNAS; GABRR2; PDE8A; GABRG2; KCNJ6; PDE1C	58	3.08E-09
2	Nicotine addiction	Human Diseases	Substance dependence	GABRQ; GABRD; CHRNA6; GRIA2; GABRE; GABRA3; CHRNB2; GABRR1; GABRR3; GABRB2; GABRG1; GRIN2B; GRIN3A; GABRP; GABRA2; GRIA4; CHRNA4; GABRG3; GABRA1; GRIA1; CACNA1B; CACNA1A; GABRA5; GRIA3; GRIN1; GABRB1; GRIN2A; GRIN3B; GABRA4; GABRA6; GABRR2; GABRB3; GRIN2C; GRIN2D; GABRG2	35	1.50E-06
3	Amphetamine addiction	Human Diseases	Substance dependence	PRKCA; MAOA; GRIA2; PRKACA; DRD1; SLC18A2; HDAC1; SLC18A1; ADCY5; CAMK2D; GRIN2B; GRIN2A; CAMK4; TH; CACNA1D; PRKCG; GRIA4; SLC6A3; GRIA1; GRIN3B; SIRT1; PPP3R1; GRIN2D; CAMK2G; PRKACB; PPP1CC; GRIA3; GRIN1; GRIN3A; PRKCB; MAOB; GRIN2C; CACNA1C; CAMK2A; GNAS; JUN; CREB1	37	0.002783
4	Alcoholism	Human Diseases	Substance dependence	PPP1CC; ADCY5; GNG2; PRKACA; HDAC9; HDAC8; MAP2K1; TH; GNAI1; MAPK3; DRD1; SLC18A2; HDAC1; SLC18A1; CREB1; GRIN2B; GRIN2A; CAMK4; DRD2; HDAC11; HDAC2; ADORA2A; NTRK2; SLC6A3; HDAC6; GRIN3B; HDAC5; GRIN2D; HDAC3; GRIN1; BRAF; MAOA; GRIN3A; SLC29A1; BDNF; HDAC4; MAOB; GRIN2C; HDAC10; GNAS; HDAC7; PKIA; RAF1; ADORA2B; MAPK1	45	0.00342
5	Endocrine resistance	Human Diseases	Drug resistance: Antineoplastic	TP53; MAPK8; ADCY5; PRKACA; MAP2K1; IGF1R; GNAS; MAPK3; ADCY2; MAPK14; GPER1; EGFR; PIK3CA; PTK2; MAP2K2; PIK3CB; ADCY1; PIK3R1; BCL2; MAPK11; MMP9; MAPK9; AKT2; PIK3CD; CYP2D6; PRKACB; BRAF; ESR1; MTOR; ESR2; MDM2; ABCB11; MMP2; MAPK10; BAD; JUN; RAF1; SRC; CDK4; MAPK12; AKT1; ERBB2; MAPK13; MAPK1	44	0.004522
6	Prion diseases	Human Diseases	Neurodegenerative diseases	HSPA5; MAP2K2; MAPK3; C1QC; PRNP; IL1B; PRKACA; FYN; SOD1; C1QB; C8B; PRKACB; C9; MAP2K1; IL6; STIP1; C5; C8A; C8G; C1QA; MAPK1	21	0.020425
7	EGFR tyrosine kinase inhibitor resistance	Human Diseases	Drug resistance: Antineoplastic	PRKCA; IL6; VEGFA; FGFR3; MAP2K1; IGF1R; MAPK3; EGFR; PIK3CA; PDGFRA; PDGFRB; MAP2K2; GSK3B; PRKCG; PIK3CB; FGF2; HGF; PIK3R1; KDR; EGF; JAK1; AKT2; PIK3CD; PDGFD; BRAF; MTOR; EIF4E; FGFR2; PRKCB; JAK2; BAD; RAF1; SRC; BCL2; MET; AKT1; ERBB2; RPS6; MAPK1	39	0.023843
8	Cocaine addiction	Human Diseases	Substance dependence	GRIA2; PRKACA; TH; GNAI1; DRD1; SLC18A2; SLC18A1; ADCY5; GRIN2B; GRIN2A; DRD2; CDK5; SLC6A3; CDK5R1; GRIN3B; NFKB1; GRIN2D; PRKACB; MAOA; GRIN1; GRIN3A; BDNF; MAOB; GRIN2C; GNAS; JUN; CREB1	27	0.02426
**Organismal systems**						
**Circulatory system**						
1	Adrenergic signaling in cardiomyocytes	Organismal Systems	Circulatory system	PRKCA; SCN5A; PPP1CC; ADCY5; MAPK11; PRKACA; AGTR1; SLC9A1; CAMK2D; MAPK3; ADCY2; ATP2A2; RYR2; ATP1A3; CACNB2; PIK3CG; MAPK13; ATP1A1; PPP2R2A; ADRA1A; KCNQ1; CACNA1S; CACNG1; CACNA1D; CACNA1F; ADCY1; CACNG2; TNNC1; SLC8A1; MAPK14; SCN1B; CACNB3; CACNB1; ADRB2; AKT2; PPP2CB; TPM1; CAMK2G; SCN7A; ADRA1B; PPP2CA; AKT1; GNAI1; CACNB4; ADRB1; PPP2R5C; CACNA1C; CAMK2A; GNAS; CACNA2D3; SCN4B; CREB1; CACNA2D2; CACNA2D1; BCL2; MAPK12; PRKACB; ADRA1D; ATP1A2; AGTR2; MAPK1	61	7.24E-06
2	Vascular smooth muscle contraction	Organismal Systems	Circulatory system	NPR1; PRKCA; PPP1CC; ADCY5; PRKACA; AGTR1; PLA2G4A; PRKCQ; KCNMB3; MAP2K1; PLA2G2A; MAPK3; ROCK2; KCNMA1; NPR2; ADCY2; PLA2G1B; ADRA1A; PRKCD; MAP2K2; CACNA1S; CACNA1D; PRKCG; CACNA1F; ADCY1; MYLK2; ADORA2A; AVPR1B; AVPR1A; CALCRL; KCNMB4; PRKACB; PTGIR; ADORA2B; ADRA1B; BRAF; MYH11; ITPR2; PRKCB; ITPR1; EDNRA; PLA2G2E; CACNA1C; ITPR3; GNAS; PRKCE; RAF1; KCNMB2; ROCK1; ADRA1D; GUCY1A2; MAPK1	52	0.004522
**Endocrine system**						
1	Estrogen signaling pathway	Organismal Systems	Endocrine system	KRT10; KRT14; KCNJ5; TFF1; HSPA8; PRKACA; PGR; CTSD; ITPR2; MAP2K1; MAPK3; ADCY2; OPRM1; GPER1; EGFR; PIK3CA; KCNJ9; ADCY5; MAP2K2; GABBR1; PIK3CB; ADCY1; PIK3R1; MMP9; HSP90AB1; GABBR2; AKT2; ESR1; KRT9; PRKACB; ITPR1; HSP90AA1; PRKCD; NOS3; GNAI1; PIK3CD; HSP90B1; ESR2; NCOA1; KCNJ3; NCOA2; HSPA2; KRT16; MMP2; ITPR3; GNAS; JUN; CREB1; RAF1; SRC; BCL2; KCNJ6; AKT1; RARA; MAPK1	55	2.95E-05

2	Renin secretion	Organismal Systems	Endocrine system	NPR1; ADCY5; AGT; AGTR1; PDE3B; EDNRA; GNAI1; KCNMA1; PTGER2; REN; ITPR2; ACE; AQP1; CACNA1S; CACNA1D; CACNA1F; PRKACA; CTSB; PDE1A; ADRB2; PDE1B; PRKACB; ADRB3; ADORA1; ADRB1; ITPR1; PPP3R1; PDE3A; CACNA1C; ITPR3; GNAS; CREB1; GUCY1A2; PDE1C	34	0.00066
3	Oxytocin signaling pathway	Organismal Systems	Endocrine system	NPR1; PRKCA; CACNG2; CAMK2A; KCNJ5; ADCY5; PRKACA; CD38; PRKAB1; PRKAA2; ITPR2; MAP2K1; GNAS; PRKAG1; MAPK3; ROCK2; EEF2; RYR2; NPR2; ADCY2; EGFR; PRKAG2; RYR1; CAMK2D; KCNJ9; CAMK4; PRKAG3; MAP2K2; CACNA1S; CACNG1; CACNA1D; PRKCG; CACNA1F; ADCY1; MYLK2; SRC; GNAI1; OXTR; PLA2G4A; KCNJ12; PRKCB; KCNJ14; CACNB3; CACNB1; PPP3R1; CAMK2G; PRKACB; PPP1CC; NOS3; PRKAB2; CACNB2; MAP2K5; CACNB4; PIK3CG; ACTB; ITPR1; KCNJ3; PTGS2; CACNA1C; ITPR3; ACTG1; CACNA2D3; JUN; CACNA2D2; RAF1; CACNA2D1; KCNJ6; ROCK1; GUCY1A2; PRKAA1; MAPK1	71	0.001189
4	Progesterone-mediated oocyte maturation	Organismal Systems	Endocrine system	MAPK8; ADCY5; PRKACA; PGR; PDE3B; CDK2; MAP2K1; IGF1R; MAPK3; RPS6KA1; MAPK14; ADCY2; CCNA2; PIK3CA; PIK3CB; ADCY1; CDK1; PIK3R1; MAPK11; HSP90AB1; RPS6KA3; MAPK9; INS; AKT2; PIK3CD; HSP90AA1; PRKACB; GNAI1; BRAF; PLK1; MAPK10; AURKA; RAF1; MAPK12; MAPK1; MAPK13; AKT1	37	0.005206
5	Insulin secretion	Organismal Systems	Endocrine system	PRKCA; CAMK2A; ADCY5; PRKACA; FFAR1; KCNMB3; GCK; ABCC8; ADCY2; RYR2; ATP1A3; KCNN3; CAMK2D; KCNJ11; ATP1A1; CACNA1S; CACNA1D; PRKCG; CACNA1F; ADCY1; KCNMA1; GLP1R; INS; CCKAR; CHRM3; KCNN4; SLC2A1; KCNMB4; CAMK2G; PRKACB; PRKCB; CACNA1C; ITPR3; GNAS; ATP1A2; CREB1; SLC2A2; KCNMB2; KCNN2	39	0.005348
6	Regulation of lipolysis in adipocytes	Organismal Systems	Endocrine system	NPR1; ADCY5; ADCY1; TSHR; GNAI1; ADCY2; PIK3CA; PTGER3; IRS1; PRKACA; PIK3R1; PIK3CB; PDE3B; INS; AKT2; ADRB2; PIK3CD; PRKACB; INSR; ADRB3; ADORA1; ADRB1; PTGS2; GNAS; PTGS1; AKT1	26	0.009561
7	Adipocytokine signaling pathway	Organismal Systems	Endocrine system	POMC; MAPK10; MAPK8; IKBKB; NFKBIA; PRKAG3; PRKAA2; PRKCQ; ACSL3; PRKAG1; RXRG; CHUK; PRKAG2; PRKAB1; TNF; PRKAB2; IRS1; RXRA; MAPK9; NFKB1; ACSL1; AKT2; PTPN11; SLC2A1; PPARA; MTOR; PCK1; PRKAA1; RXRB; JAK2; ACSL4; CPT1A; ACACB; AKT1	34	0.011776
8	Thyroid hormone signaling pathway	Organismal Systems	Endocrine system	PRKCA; THRB; SLC9A1; PRKACA; TP53; PRKCB; MAP2K1; BAD; MAPK3; RXRG; HDAC1; ATP1A3; ITGAV; PIK3CA; ATP1A1; MAP2K2; ITGB3; GSK3B; PRKCG; PIK3CB; DIO3; HDAC2; ATP2A2; RXRA; AKT2; ESR1; SLC2A1; PRKACB; HDAC3; PIK3CD; MTOR; HIF1A; MDM2; ACTB; NCOA1; NCOA2; KAT2B; THRA; RXRB; ACTG1; PDPK1; RAF1; SRC; PIK3R1; AKT1; ATP1A2; MAPK1	47	0.014207
9	Insulin signaling pathway	Organismal Systems	Endocrine system	AKT2; GSK3B; MAPK8; IKBKB; PRKACA; PRKAG1; PDE3B; PRKAG3; PRKAA2; MAP2K1; PYGB; MAPK3; PRKAG2; PIK3CA; FBP1; PRKCZ; PYGM; PRKAB1; MAP2K2; PRKAB2; PTPN1; PIK3CB; PIK3R1; IRS1; PYGL; MAPK9; INS; PRKCI; PIK3CD; PRKACB; PPP1CC; INSR; BRAF; MTOR; EIF4E; PRKAR1A; SREBF1; PRKAA1; MAPK10; BAD; PDPK1; RAF1; PCK1; AKT1; GCK; ACACB; RPS6; MAPK1	48	0.017913
10	Relaxin signaling pathway	Organismal Systems	Endocrine system	PRKCA; VEGFA; ADCY5; GNG2; NOS2; PRKACA; NFKBIA; MAP2K1; MAPK3; TGFBR2; MAPK14; ADCY2; EGFR; PIK3CA; CREB1; PRKCZ; TGFBR1; MAP2K2; TGFB1; PIK3CB; ADCY1; EDNRB; PIK3R1; MAPK11; MMP9; MAPK9; NFKB1; AKT2; PIK3CD; NOS1; PRKACB; NOS3; MAPK8; GNAI1; MMP2; MAPK10; GNAS; JUN; MMP1; RAF1; SRC; MMP13; MAPK12; AKT1; MAPK13; MAPK1	46	0.018252
11	GnRH signaling pathway	Organismal Systems	Endocrine system	PRKCA; MAPK10; MAPK8; ADCY5; PRKACA; PLA2G4A; MAP2K1; MAPK3; MAPK14; ADCY2; EGFR; ITPR2; CAMK2D; MAP2K2; CACNA1S; CACNA1D; CACNA1F; ADCY1; CAMK2A; MAPK11; MAPK9; CAMK2G; PRKACB; PTK2B; MAP3K1; PRKCB; GNRHR; ITPR1; MMP2; CACNA1C; ITPR3; GNAS; JUN; RAF1; SRC; MAP3K2; MAPK12; MAPK1; MAPK13; PRKCD	40	0.029206
12	Melanogenesis	Organismal Systems	Endocrine system	PRKCA; POMC; ADCY5; PRKACA; MAP2K1; MAPK3; ADCY2; CAMK2D; MAP2K2; GSK3B; PRKCG; ADCY1; EDNRB; TYR; CAMK2G; PRKACB; GNAI1; KIT; PRKCB; LEF1; CAMK2A; GNAS; CREB1; RAF1; MAPK1	25	0.039513
13	Glucagon signaling pathway	Organismal Systems	Endocrine system	PGAM2; CAMK2A; PRKACA; PRKAG1; PDE3B; PFKFB1; PRKAG3; PRKAA2; GCK; PYGB; ADCY2; PRKAG2; ITPR2; LDHB; FBP1; PYGM; PRKAB1; PGAM1; PRKAB2; PYGL; SLC2A2; AKT2; SLC2A1; PPP3R1; LDHA; PRKACB; SIRT1; SIK2; ITPR1; CAMK2D; PCK1; PRKAA1; ITPR3; GNAS; CREB1; CPT1A; CAMK2G; PKM; AKT1; ACACB; PPARA	41	0.049058
**Immune system**						
1	Complement and coagulation cascades	Organismal Systems	Immune system	SERPIND1; KNG1; A2M; C4BPB; F5; KLKB1; C1R; CPB2; PLAU; SERPINE1; F12; CLU; VTN; MASP2; F10; FGB; PROCR; C1S; THBD; C4BPA; CFB; FGA; F2; F2R; SERPINC1; F11; C5; ITGB2; BDKRB1; MBL2; C1QC; C1QB; F9; PROC; PROS1; C8B; F7; BDKRB2; PLG; C8G; F8; C8A; SERPINA1; F13B; PLAUR; C3; SERPING1; PLAT; CFI; SERPINF2; C1QA; C9; CFH; FGG; VWF	55	4.12E-06
2	Platelet activation	Organismal Systems	Immune system	AKT2; TBXA2R; ADCY5; P2RY12; PRKACA; PLA2G4A; ITPR2; GNAS; MAPK3; ROCK2; MAPK14; ADCY2; PIK3CG; PRKCZ; FYN; FGA; ITGB3; LYN; F2R; PIK3CB; ADCY1; MYLK2; GP1BA; PIK3R1; TBXAS1; MYL12A; MAPK11; FCGR2A; BTK; FGB; PRKCI; PIK3CD; PTGIR; PRKACB; PPP1CC; NOS3; GNAI1; PIK3CA; ACTB; ITPR1; FGG; ITPR3; ACTG1; PTGS1; SYK; SRC; ITGA2B; MAPK12; ROCK1; AKT1; GUCY1A2; MAPK13; VWF; MAPK1	54	0.0015
3	IL-17 signaling pathway	Organismal Systems	Immune system	IL6; IL17A; MAPK8; IL13; IKBKB; NFKBIA; S100A7; MAPK3; IL5; MMP3; MAPK14; CCL2; CHUK; SRSF1; CEBPB; CXCL10; MMP13; MAPK11; MMP9; HSP90AB1;	40	0.011974

				S100A8; MAPK9; NFKB1; CXCL8; HSP90AA1; HSP90B1; TNF; IL1B; CASP8; PTGS2; MAPK10; IFNG; CASP3; JUN; MMP1; GSK3B; MAPK12; MAPK1; MAPK13; S100A9		
4	Th17 cell differentiation	Organismal Systems	Immune system	IL2RB; IL6; IL17A; TGFBR1; IL2RG; IKBKB; IFNG; NFKBIA; PRKCQ; JAK3; MAPK3; RXRG; TGFBR2; MAPK14; CHUK; MAPK10; CD3E; IL2; TGFB1; AHR; IL1R1; MAPK11; HSP90AB1; RXRA; JAK1; MAPK9; NFKB1; PPP3R1; HSP90AA1; STAT5B; MAPK8; IL2RA; CD4; MTOR; IFNGR2; HIF1A; TYK2; IL1B; LCK; RXRB; JAK2; MAPK13; JUN; ZAP70; MAPK12; RARA; MAPK1	47	0.04154
**Nervous system**						
1	Serotonergic synapse	Organismal Systems	Nervous system	PRKCA; ITPR3; KCNJ5; MAOA; ADCY5; GNG2; HTR7; PLA2G4A; HTR3B; MAOB; MAP2K1; HTR3D; HTR2B; MAPK3; SLC18A2; CYP2C8; GABRB2; HTR6; SLC18A1; HTR5A; ALOX15; KCNJ9; HTR1B; HTR1D; ALOX5; HTR3E; CACNA1S; SLC6A4; CACNA1D; PRKCG; CACNA1F; PRKACA; HTR2A; CYP2C9; KCND2; HTR3C; HTR1A; HTR2C; CACNA1B; CACNA1A; HTR1E; HTR1F; CYP2D6; ITPR1; PRKACB; CYP2C19; GNAI1; BRAF; GABRB1; ITPR2; PRKCB; HTR3A; KCNJ3; PTGS2; HTR4; CACNA1C; GABRB3; CASP3; GNAS; PTGS1; RAF1; APP; KCNJ6; KCNN2; MAPK1	65	2.89E-08
2	Dopaminergic synapse	Organismal Systems	Nervous system	PRKCA; DRD5; MAPK10; KCNJ5; MAOA; GRIA2; GNG2; PRKACA; CALY; MAOB; ADCY5; TH; SCN1A; DRD1; SLC18A2; MAPK8; MAPK14; DRD3; ITPR2; CAMK2D; KCNJ9; GRIN2B; PPP2R2A; DRD2; CACNA1D; PRKCG; GRIA4; CAMK2A; MAPK11; SLC6A3; GRIA1; MAPK9; CACNA1B; CACNA1A; SLC18A1; AKT2; PPP2CB; CAMK2G; PRKACB; PPP1CC; PPP2CA; GRIA3; GNAI1; DRD4; GRIN2A; PRKCB; ITPR1; KCNJ3; CACNA1C; ITPR3; GNAS; COMT; CREB1; GSK3B; KCNJ6; MAPK12; PPP2R5C; MAPK13; AKT1	59	2.56E-06
3	Cholinergic synapse	Organismal Systems	Nervous system	KCNQ2; PRKCA; AKT2; CAMK2A; CHRNA6; CHRM4; GNG2; PRKACA; ITPR2; ADCY5; MAP2K1; CHRNB2; MAPK3; ADCY2; PIK3CG; CAMK2D; FYN; CACNA1S; CACNA1D; PRKCG; CACNA1F; ADCY1; KCNQ5; CHRNA4; PIK3R1; CHRM2; CHRM1; PIK3CB; CAMK4; KCNJ14; CACNA1B; CACNA1A; KCNJ12; CHRM3; PIK3CD; CHAT; CAMK2G; CHRNB4; CHRM5; GNAI1; PIK3CA; KCNQ1; PRKCB; ITPR1; KCNJ3; CHRNA3; CACNA1C; ITPR3; JAK2; ACHE; CREB1; KCNQ3; BCL2; KCNJ6; PRKACB; AKT1; MAPK1	57	4.98E-06
4	GABAergic synapse	Organismal Systems	Nervous system	PRKCA; GABRD; ADCY5; GNG2; PRKACA; GABRQ; GABRE; GABRA3; GABRR1; GABRR3; GABRB2; ADCY2; GABRG1; GABRP; CACNA1S; GABBR1; CACNA1D; PRKCG; CACNA1F; GABRA2; ABAT; GABRG3; GABRA1; CACNA1B; CACNA1A; GABRA5; ADCY1; PRKACB; GNAI1; GABRB1; PRKCB; GPHN; SLC6A1; GABRA4; GABBR2; GABRA6; GABRR2; CACNA1C; GABRB3; SRC; GABRG2; KCNJ6	42	0.018585
5	Neurotrophin signaling pathway	Organismal Systems	Nervous system	TP53; GSK3B; MAPK8; IKBKB; NFKBIA; MAPKAPK2; MAP2K1; SH2B3; IRAK4; MAPK3; RPS6KA1; MAPK14; PIK3CA; CAMK2D; PDPK1; NGF; TP73; MAP2K2; RAC1; ABL1; PIK3CB; PIK3R1; CAMK2A; NTRK2; MAPK11; IRS1; NTRK1; RPS6KA3; CAMK4; MAPK9; NFKB1; AKT2; PIK3CD; CAMK2G; PRKCD; BRAF; PTPN11; MAP3K1; MAP2K5; BDNF; MAPK10; BAD; JUN; RAF1; BCL2; MAPK12; AKT1; MAPK13; MAPK1	49	0.029174
6	Long-term potentiation	Organismal Systems	Nervous system	PRKCA; CAMK2A; GRIA2; PRKACA; MAP2K1; MAPK3; RPS6KA1; ITPR2; CAMK2D; GRIN2B; MAP2K2; PRKCG; ADCY1; GRIA1; RPS6KA3; CAMK4; PPP3R1; GRIN2D; CAMK2G; PRKACB; PPP1CC; GRIN1; BRAF; GRIN2A; GRM5; PRKCB; ITPR1; GRIN2C; CACNA1C; ITPR3; RAF1; MAPK1	32	0.048865
**Digestive system**						
1	Pancreatic secretion	Organismal Systems	Digestive system	PRKCA; SLC9A1; ADCY5; ADCY1; CD38; PRSS1; CPB2; AMY2A; CPB1; CPA1; SCTR; CFTR; RYR2; BST1; ATP1A3; ITPR2; SLC12A2; ATP1A1; RAC1; PRKCG; PRSS2; ATP2A1; AMY2B; CA2; KCNMA1; ATP2A2; PNLIPRP2; CCKAR; CHRM3; ADCY2; PNLIP; CTRB1; PLA2G2A; KCNQ1; PRKCB; ITPR1; PLA2G2E; ITPR3; GNAS; PRSS3; ATP1A2; PLA2G1B	42	7.66E-05
2	Salivary secretion	Organismal Systems	Digestive system	PRKCA; SLC9A1; ADCY5; PRKACA; CD38; KCNMA1; BST1; ATP1A3; LYZ; ITPR2; SLC12A2; ATP1A1; ADRA1A; PRKCG; ADCY1; ADRB2; ADCY2; CHRM3; KCNN4; NOS1; PRKACB; ADRA1B; ADRB3; ADRB1; PRKCB; ITPR1; ITPR3; GNAS; GUCY1A2; ATP1A2; ADRA1D	31	0.000714
3	Gastric acid secretion	Organismal Systems	Digestive system	PRKCA; SLC9A1; ADCY5; ATP4B; SSTR2; PRKACA; KCNJ1; CFTR; ATP1A3; ITPR2; CAMK2D; ATP1A1; PRKCG; ADCY1; MYLK2; HRH2; CA2; KCNJ10; CAMK2A; PRKCB; ADCY2; CHRM3; CAMK2G; PRKACB; GNAI1; KCNQ1; ACTB; ITPR1; ITPR3; GNAS; KCNK2; ATP4A; ATP1A2; KCNJ15	34	0.00342
**Others**						
1	Circadian entrainment	Organismal Systems	Environmental adaptation	MTNR1B; PRKCA; CAMK2A; KCNJ5; CACNA1H; GRIA2; GNG2; PRKACA; GNAI1; MAPK3; RYR2; ADCY2; RYR1; CAMK2D; KCNJ9; GRIN2B; ADCY5; CACNA1D; PRKCG; ADCY1; GRIA4; GRIA1; MTNR1A; NOS1; GRIN2D; CAMK2G; PRKACB; GRIA3; GRIN1; GRIN2A; CACNA1I; PRKCB; ITPR1; KCNJ3; GRIN2C; CACNA1C; ITPR3; GNAS; CREB1; KCNJ6; GUCY1A2; CACNA1G; MAPK1	43	1.69E-06
2	Inflammatory mediator regulation	Organismal Systems	Sensory system	PRKCA; MAPK10; TRPV3; ADCY5; MAPK11; PRKACA; PLA2G4A; PRKCQ; TRPV2; HTR2B; MAPK8; MAPK14; PTGER2; ADCY2; PIK3CB; PIK3CA; CAMK2D; NGF; PRKCG; HTR2C; ADCY1; HTR2A; PIK3R1; PRKCE; BDKRB1; NTRK1; P2RY2; MAPK9; TRPA1; IL1R1; PIK3CD; ITPR2; CAMK2G; PRKCD; PPP1CC; MAPK12;	50	0.001462

	of TRP channels			CAMK2A; TRPV4; HRH1; PRKCB; ITPR1; IL1B; TRPV1; ITPR3; GNAS; SRC; TRPM8; BDKRB2; MAPK13; PRKACB		
3	Longevity regulating pathway - multiple species	Organismal Systems	Aging	HSPA8; PRKACA; PRKAB1; PRKAA2; IGF1R; PRKAG1; HDAC1; ADCY2; PRKAG2; PIK3CA; PRKAG3; ADCY5; PRKAB2; PIK3CB; ADCY1; HDAC2; IRS1; INS; AKT2; PIK3CD; PRKACB; INSR; SIRT1; MTOR; HSPA2; PRKAA1; SOD1; PIK3R1; AKT1	29	0.014207
4	Endocrine and other factor-regulated calcium reabsorption	Organismal Systems	Excretory system	PRKCB; ATP1A3; PTH1R; VDR; PRKCA; ATP1A1; PRKACA; GNAS; ESR1; PRKCG; BDKRB2; SLC8A1; KLK1; ATP1A2; PRKACB	15	0.029174
5	Taste transduction	Organismal Systems	Sensory system	SCN9A; PRKACA; HTR3B; GABRA3; HTR3D; HTR3C; HTR1B; HTR3E; HTR1D; SCN3A; PDE1A; GABRA2; HTR1A; GABRA1; CACNA1A; HTR1E; CHRM3; HTR1F; PDE1B; GABRA5; PRKACB; SCN2A; GABBR1; HTR3A; GABRA4; GABBR2; GABRA6; CACNA1C; ITPR3; PDE1C	30	0.029696
**Cellular processes**						
1	Gap junction	Cellular Processes	Cellular community - eukaryotes	PRKCA; TUBA1C; ADCY5; PRKACA; TUBB4A; MAP2K1; TUBB1; HTR2B; MAPK3; DRD1; TUBB3; ADCY2; EGFR; ITPR2; TUBB6; PDGFRA; DRD2; PDGFRB; MAP2K2; TUBB4B; PRKCG; HTR2C; ADCY1; HTR2A; PDGFD; CDK1; TUBB; TUBB2B; EGF; TUBA1B; TUBA3E; PRKACB; GNAI1; GRM5; MAP2K5; ADRB1; PRKCB; ITPR1; TUBA1A; ITPR3; GNAS; RAF1; SRC; MAP3K2; TUBA8; TUBB2A; MAPK1; GUCY1A2; TUBA4A	49	4.10E-05
2	Tight junction	Cellular Processes	Cellular community - eukaryotes	TUBA1C; MAPK8; PRKACA; MYH14; CD1D; PRKAB1; PRKAA2; MYL12A; PRKAG1; CFTR; PRKAG2; MAP3K1; PRKCZ; PPP2R2A; ACTN1; TUBA8; PRKAG3; ROCK2; RAC1; PRKAB2; CACNA1D; RAB8B; PRKCE; MYH9; MAPK9; TUBA1B; PRKCI; CD1A; PPP2CB; TUBA3E; PRKACB; PPP2CA; MYH11; ACTB; TUBA1A; PRKAA1; MAPK10; ACTG1; JUN; SRC; CDK4; ROCK1; ERBB2; TUBA4A	44	0.007643
3	Apoptosis	Cellular Processes	Cell growth and death	TP53; AKT1; TUBA1C; MAPK8; IKBKB; CTSB; NFKBIA; CTSD; MAP2K1; BAD; PARP3; CYCS; CTSS; MAPK3; CHUK; PIK3CA; CTSK; PDPK1; TNFRSF10B; MAPK10; NGF; MAP2K2; BIRC5; PIK3CB; PARP2; PIK3R1; CTSV; NTRK1; ATM; MAPK9; NFKB1; TUBA1B; CTSF; AKT2; PIK3CD; CASP7; TUBA3E; CTSH; CTSL; PARP1; TNF; ITPR2; ACTB; ITPR1; CASP8; TUBA1A; ITPR3; CASP3; ACTG1; JUN; RAF1; BCL2; TUBA8; MAPK1; IL3; TUBA4A	56	0.010417
4	Autophagy - animal	Cellular Processes	Transport and catabolism	BECN1; PRKACA; CTSD; MAPK1; PRKAA2; PRKCQ; MAP2K1; IGF1R; CTSL; MAPK3; MAPK8; PIK3CA; PDPK1; MAP2K2; PIK3CB; CTSB; HMGB1; PIK3R1; IRS1; MAPK9; INS; AKT2; PIK3CD; PPP2CB; PRKCD; PPP2CA; PIK3C3; MTOR; ULK1; ITPR1; HIF1A; DAPK3; PRKAA1; MAPK10; BAD; RAF1; BCL2; AKT1; DAPK1; PRKACB	40	0.018772
5	Oocyte meiosis	Cellular Processes	Cell growth and death	CAMK2A; ADCY5; PRKACA; PGR; CDK2; SLK; MAP2K1; IGF1R; MAPK3; RPS6KA1; ADCY2; ITPR2; CAMK2D; ADCY1; YWHAB; CDK1; SKP1; RPS6KA3; INS; AR; YWHAH; PPP2CB; PPP3R1; CAMK2G; PRKACB; PPP1CC; PPP2CA; PLK1; ITPR1; ITPR3; AURKA; MAPK12; PPP2R5C; MAPK1	34	0.030356
**Metabolism**						
1	Purine metabolism	Metabolism	Nucleotide metabolism	PDE5A; PDE4B; RRM2B; ADCY5; ENPP1; ADCY1; PDE3B; PDE4A; ADK; ATIC; ADSSL1; PDE6B; HPRT1; PNP; NT5C2; GMPR; ADA; ADCY2; PDE6A; PAPSS1; IMPDH2; PDE6G; FHIT; NT5E; NPR2; POLE3; PDE1A; PPAT; RRM1; PDE7A; POLE2; POLE; PDE4D; PDE2A; PDE10A; POLA1; GART; RRM2; PDE1B; NPR1; PDE11A; DCK; PDE8B; XDH; PDE6H; PDE4C; PGM1; ADSS; PDE7B; PDE3A; PDE9A; NME1; APRT; POLE4; PDE6C; PDE8A; NT5M; PKM; GUCY1A2; IMPDH1; PDE1C	61	8.64E-05
2	Steroid hormone biosynthesis	Metabolism	Lipid metabolism	SRD5A1; CYP1A2; SULT2B1; AKR1C3; STS; HSD3B1; CYP19A1; HSD17B2; CYP2E1; CYP21A2; CYP3A4; AKR1C1; SULT1E1; CYP1A1; CYP11B2; CYP17A1; CYP11B1; HSD11B1; CYP3A5; HSD17B8; UGT1A9; SRD5A2; CYP1B1; HSD11B2; AKR1C2; COMT; HSD17B1	27	0.039513
**Genetic information processing**						
1	Proteasome	Genetic Information Processing	Folding, sorting and degradation	PSMB6; PSMB9; PSMA4; PSMB4; PSMB7; PSMA2; IFNG; PSMB8; PSMB5; PSMA1; PSMA6; PSMA3; PSMB10; PSME1; PSMA7; PSMB1; PSMB2; PSMB3	18	0.010417
**Pathways Enriched in Targets of Autophagy Inhibitors (p-value < 0.05)**						

**Signal transduction**						
**No.**	**Pathway**	**Class**	**Subclass**	**Targets**	**No. Targets**	**p-value**
1	MAPK signaling pathway	Environmental Information Processing	Signal transduction	MAP3K13; TGFBR1; IKBKB; MAP4K1; MAP2K6; PAK1; MAP4K3; FGFR1; CHUK; EGFR; MAP3K1; PDGFRA; CACNA1S; CACNA1F; IRAK1; MAPK11; MAP2K3; CACNA1A; TAOK1; KIT; CACNB4; FGF4; CACNA2D2; RAF1; CACNA1H; PLA2G4A; MKNK1; MAPKAPK2; PAK2; FGF19; IGF1R; FLT1; MAPK3; IGF1; MAP2K2; CACNG1; ERBB4; MAP3K12; KDR; MAPK9; AKT2; PRKACB; MAP3K6; BRAF; MAP2K5; CACNA2D1; MAP3K2; FGF1; MET; TEK; MAPK1; VEGFA; HSPA8; TAOK3; CSF1R; IRAK4; RPS6KA1; ZAK; PDGFRB; MAP3K3; PRKCG; NTRK2; FGFR3; MAP4K4; CACNB3; CACNB1; FGFR4; TNF; FLT4; PRKCB; MAPK10; CASP3; STK3; ERBB2; MAPK13; CACNA1G; AKT1; PRKACA; MAPKAPK5; MAP3K4; CACNA1E; HGF; TGFBR2; MAPK14; CACNB2; MKNK2; NFKB2; EPHA2; MAPT; NTRK1; RPS6KA3; CACNA1B; INSR; MAPK7; FGFR2; MAP3K11; IL1B; FLT3; CACNA1C; RPS6KA6; TAOK2; FGF2; MAP4K2	103	1.30E-17
2	Ras signaling pathway	Environmental Information Processing	Signal transduction	VEGFA; IKBKB; PRKACA; PLA2G4A; CSF1R; PIK3R2; PIK3R3; PAK2; FGF19; IGF1R; PAK1; FLT1; PLA2G2A; HGF; HTR7; FGFR1; NTRK1; IGF1; PAK5; TBK1; PAK3; GRIN2B; PDGFRA; PLA2G1B; PDGFRB; MAP2K2; MAPK3; ABL1; PRKCG; PIK3CB; MAPK9; EPHA2; PIK3R1; NTRK2; PAK4; ABL2; KDR; FGFR3; AKT2; PIK3CD; PLA2G6; EGFR; PRKACB; INSR; GRIN1; FGFR4; KIT; FLT4; PIK3CA; FGFR2; PRKCB; CHUK; FLT3; PLA2G2E; FGF4; MAPK10; ZAP70; RAF1; FGF2; FGF1; MET; MAPK1; TEK; AKT1	64	1.32E-09
3	ErbB signaling pathway	Environmental Information Processing	Signal transduction	CAMK2A; PIK3R2; PIK3R3; PAK2; PAK1; MAPK3; CAMK2B; PAK5; EGFR; PAK3; CAMK2D; PTK2; MAP2K2; ABL1; GSK3B; PRKCG; PIK3CB; MAPK9; PIK3R1; PAK4; ABL2; ERBB4; AKT2; PIK3CD; CAMK2G; STAT5B; BRAF; MTOR; PIK3CA; PRKCB; MAPK10; RAF1; SRC; MAPK1; ERBB2; AKT1	36	7.16E-08
4	Calcium signaling pathway	Environmental Information Processing	Signal transduction	DRD5; PDE1A; ADRA1B; BDKRB1; NOS2; PRKACA; AGTR1; EDNRA; CACNA1E; GRIN1; HTR2B; MYLK4; CAMK2B; CACNA1H; HTR6; PTAFR; HTR5A; CAMK2D; DRD1; PDGFRA; CACNA1S; PDGFRB; ADRA1A; F2R; PRKCG; HTR2C; HTR7; MYLK2; PHKG1; TNNC1; MYLK3; ADRB2; CACNA1B; CHRM2; CHRM1; PPIF; HRH2; HTR2A; CACNA1F; ERBB4; AVPR1A; ADORA2B; CHRM3; MYLK; EGFR; CAMK2G; PRKACB; CHRM5; NOS3; ADRB3; PTK2B; PDE1B; HRH1; ADRB1; PRKCB; EDNRB; NOS1; HTR4; CACNA1C; CAMK2A; PLCD1; OXTR; TACR1; CACNA1A; ADORA2A; ERBB2; CACNA1G; ADRA1D	68	2.27E-07
5	mTOR signaling pathway	Environmental Information Processing	Signal transduction	ULK2; IKBKB; ATP6V1A; PIK3R2; PIK3R3; PRKAA2; IGF1R; MAPK3; RPS6KA1; IGF1; CHUK; PIK3CA; PDPK1; MTOR; MAP2K2; RAF1; PRKCG; PIK3CB; PIK3R1; RPS6KA3; PRKCB; AKT2; PIK3CD; INSR; BRAF; TNF; EIF4E; ULK1; PRKAA1; RPS6KA6; GSK3B; SGK1; MAPK1; AKT1	34	8.11E-07
6	Rap1 signaling pathway	Environmental Information Processing	Signal transduction	AKT2; VEGFA; PRKD1; MAPK3; CSF1R; MAPK1; FGF19; MAP2K6; FLT1; HGF; FGFR1; MAPK14; EGFR; PIK3CA; GRIN2B; PDGFRA; DRD2; PDGFRB; MAP2K2; F2R; PRKCG; PIK3CB; EPHA2; FGF2; IGF1; PIK3R1; ADORA2A; MAPK11; KDR; PRKCB; FGFR3; PRKCI; PIK3CD; INSR; GRIN1; BRAF; FGFR4; KIT; FLT4; PIK3R2; FGFR2; MAP2K3; FGF4; RAF1; SRC; FGF1; PIK3R3; IGF1R; MET; ADORA2B; TEK; MAPK13; AKT1	53	3.20E-06
7	PI3K-Akt signaling pathway	Environmental Information Processing	Signal transduction	ITGA4; IL6; TLR4; VEGFA; BCL2; IKBKB; MAPK3; NOS3; CSF1R; IL2RB; CDK6; PIK3R3; PRKAA2; FGF19; JAK3; IGF1R; FLT1; HGF; FGFR1; NTRK1; IGF1; CHUK; EGFR; PIK3CG; PDPK1; AKT2; PTK2; PDGFRA; IFNAR1; PDGFRB; MAP2K2; IL2; GSK3B; SYK; EPHA2; EIF4E; PIK3R1; CHRM2; NTRK2; CHRM1; RXRA; HSP90AB1; PRLR; KDR; PIK3CB; COL1A1; JAK1; ERBB4; PIK3R2; PKN2; PIK3CD; FGFR3; HSP90AA1; MTOR; INSR; FGFR4; HSP90B1; KIT; FLT4; PIK3CA; FGFR2; CDK2; FLT3; TEK; FGF4; JAK2; F2R; FN1; RAF1; FGF2; CDK4; FGF1; PKN1; SGK1; MET; MAPK1; ERBB2; PRKAA1; AKT1	79	7.66E-06
8	FoxO signaling pathway	Environmental Information Processing	Signal transduction	IL6; TGFBR1; IKBKB; PLK4; PRKAG3; PRKAA2; IGF1R; PRKAG1; MAPK3; BRAF; TGFBR2; MAPK14; CHUK; EGFR; PRKAG2; PIK3CA; PDPK1; PRKAB1; MAP2K2; PRKAB2; PIK3CB; IGF1; PIK3R1; MAPK11; STAT3; MAPK9; AKT2; PIK3CD; INSR; PLK3; PIK3R3; PIK3R2; PLK1; CDK2; PRKAA1; MAPK10; RAF1; PLK2; SGK1; MAPK1; MAPK13; AKT1	42	1.00E-05
9	cGMP-PKG signaling pathway	Environmental Information Processing	Signal transduction	PDE5A; ADRA2B; ADORA3; ADRA1B; PDE2A; AGTR1; NOS3; PDE3B; MAPK1; EDNRA; KCNMB3; MAPK3; MYLK4; ROCK2; CNGA1; PRKG2; PIK3CG; ATP1A1; ADRA1A; MAP2K2; CACNA1S; CACNA1F; MYLK2; EDNRB; ADRA2A; PRKCE; KCNMA1; PPIF; OPRD1; PDE2A; NPPB; AKT2; ADRB2; KCNMB4; ADRA2C; INSR; ADRB3; ADORA1; ADRB1; MYLK3; MYLK; PDE3A; CACNA1C; KCNMB1; RAF1; ROCK1; ADRA1D; GUCY1A2; AKT1	49	6.66E-05
10	Apelin signaling pathway	Environmental Information	Signal transduction	SLC9A1; BECN1; NOS2; PRKACA; AGTR1; PDE3B; PRKAG3; PRKAA2; PRKAG1; MAPK3; MYLK4; TGFBR1; PRKAG2; PIK3CG; PRKAB1; MAP2K2; PRKAB2; MYLK2; MYLK3; PRKCE; AKT2; MYLK; PRKACB; NOS3; PIK3C3; MTOR; HDAC4; NOS1; PRKAA1; PLAT; RAF1; MAPK1; AKT1	33	0.00011

		Processing				
11	cAMP signaling pathway	Environmental Information Processing	Signal transduction	PDE4B; DRD5; MAPK10; GRIA2; PRKACA; PDE3B; PDE4A; MAPK1; EDNRA; SLC9A1; PAK1; MAPK3; DRD1; CAMK2B; CNGA1; HTR6; ATP1A1; PIK3CA; CAMK2D; HTR1B; AKT2; HCN2; F2R; DRD2; ROCK2; MAP2K2; CACNA1S; HTR1D; CACNA1F; PDE4D; PIK3R1; ADORA2A; CHRM1; HTR1A; CFTR; PIK3CB; MAPK9; HTR1E; ADRB2; PIK3CD; CAMK2G; PRKACB; AKT1; GRIN1; BRAF; HTR1F; GRIN3A; ADORA1; PIK3R2; ADRB1; GRIN3B; PDE3A; HTR4; CACNA1C; CAMK2A; OXTR; GRIN2B; RAF1; PIK3R3; ROCK1; CHRM2; PPARA	62	0.000136
12	VEGF signaling pathway	Environmental Information Processing	Signal transduction	VEGFA; PLA2G4A; MAPKAPK2; PIK3R3; MAPK3; MAPK14; PIK3R2; PTK2; MAP2K2; PRKCG; PIK3CB; PIK3R1; MAPK11; KDR; AKT2; PIK3CD; NOS3; PIK3CA; PRKCB; PTGS2; RAF1; SRC; AKT1; MAPK13; MAPK1	25	0.000258
13	AMPK signaling pathway	Environmental Information Processing	Signal transduction	PFKFB1; PRKAB1; PRKAA2; IGF1R; PRKAG1; CAMKK2; CFTR; IGF1; PRKAG2; PIK3CA; FBP1; PDPK1; ADRA1A; PRKAG3; PRKAB2; PIK3CB; PIK3R1; HMGCR; AKT2; PIK3CD; INSR; CCNA2; PIK3R3; MTOR; PIK3R2; ULK1; PPARG; SREBF1; PRKAA1; AKT1	30	0.000275
14	NF-kappa B signaling pathway	Environmental Information Processing	Signal transduction	IKBKB; CSNK2A2; PLAU; XIAP; IRAK4; RIPK1; CSNK2A1; LYN; SYK; VCAM1; IRAK1; BTK; NFKB2; CHUK; ICAM1; TLR4; CSNK2B; PARP1; TNF; TNFRSF11A; IL1B; PRKCB; PTGS2; LCK; PRKCQ; ZAP70; BCL2	27	0.00037
15	HIF-1 signaling pathway	Environmental Information Processing	Signal transduction	IL6; VEGFA; NOS2; NOS3; MKNK1; PIK3R2; PIK3R3; IGF1R; FLT1; MAPK3; CAMK2B; IGF1; EGFR; PIK3CA; CAMK2D; MKNK2; MAP2K2; TF; PRKCG; PIK3CB; PIK3R1; STAT3; AKT2; PIK3CD; SLC2A1; CAMK2G; TLR4; INSR; MTOR; EIF4E; PRKCB; HIF1A; ERBB2; CAMK2A; BCL2; MAPK1; TEK; AKT1	38	0.000815
16	TNF signaling pathway	Environmental Information Processing	Signal transduction	IL6; IKBKB; PIK3R2; PIK3R3; MAP2K6; MAPK3; CSF2; MMP3; MAPK14; CHUK; PIK3CA; PIK3CB; VCAM1; RIPK1; PIK3R1; MAPK11; SELE; MAPK9; AKT2; PIK3CD; CASP7; ICAM1; TNF; PTGS2; MAP2K3; IL1B; MAPK10; CASP3; AKT1; MAPK13; MAPK1	31	0.004275
17	Sphingolipid signaling pathway	Environmental Information Processing	Signal transduction	ADORA3; CTSD; PIK3R3; MAPK3; ROCK2; MAPK14; PIK3CB; PIK3CA; PDPK1; FYN; MAP2K2; PRKCG; ABCC1; PIK3R1; PRKCE; MAPK11; OPRD1; MAPK9; AKT2; PIK3CD; NOS3; TNF; ADORA1; PIK3R2; PRKCB; MAPK10; RAF1; BCL2; ROCK1; MAPK1; MAPK13; AKT1	32	0.01183
**Human diseases**						
**Cancers: Overview**						
1	Pathways in cancer	Human Diseases	Cancers: Overview	TGFBR1; IKBKB; AGTR1; PIK3R3; CDK2; EDNRA; XIAP; ROCK2; FGFR1; CHUK; EGFR; PIK3R2; CAMK2D; PDGFRA; MTOR; NOS2; ABL1; EDNRB; STAT3; HSP90AB1; JAK1; AR; CAMK2G; KIT; NQO1; FGF4; JAK2; GSTA1; GSK3B; NCOA1; RARA; BCR; FGFR3; CDK6; FGF19; IGF1R; MAPK3; RXRG; IGF1; RET; MAP2K2; RAF1; BDKRB1; MAPK9; AKT2; ESR1; PRKACB; BRAF; DAPK2; PPARG; RXRB; FN1; GSTM4; BCL2; FGF1; MET; MAPK1; IL12B; VEGFA; CSF1R; FLT4; GSTM1; IFNAR1; PDGFRB; PRKCG; PIK3CB; HDAC2; GSTA2; ALK; RXRA; NFKB2; PIK3CD; SLC2A1; HSP90AA1; FGFR4; ESR2; PRKCB; PTGS2; GSTP1; MAPK10; CASP3; PIM1; CDK4; ROCK1; ERBB2; DAPK1; AKT1; IL2RB; IL6; PRKACA; JAK3; HGF; TGFBR2; PIK3CA; PTK2; CAMK2B; IL2; F2R; PIK3R1; NTRK1; CASP7; HSP90B1; FGFR2; HIF1A; DAPK3; FLT3; CAMK2A; MMP1; STAT5B; FGF2; PPARD; TXNRD1; CSF2RA	113	1.25E-06
2	Proteoglycans in cancer	Human Diseases	Cancers: Overview	IL12B; SLC9A1; VEGFA; PRKACA; MAPK1; PLAU; IGF1R; PAK1; HGF; CAMK2B; FGFR1; MAPK14; EGFR; PIK3CA; CAMK2D; PDPK1; PTK2; TNF; MAP2K2; PRKCG; PIK3CB; SRC; IGF1; PIK3R1; MAPK11; STAT3; KDR; ERBB4; AKT2; ESR1; MAPK3; CAMK2G; PRKACB; TLR4; BRAF; PIK3CD; ROCK2; MTOR; PIK3R2; PRKCB; HIF1A; CAMK2A; CASP3; FN1; RAF1; FGF2; ROCK1; PIK3R3; MET; AKT1; ERBB2; MAPK13; CTSL	53	0.000113
3	Central carbon metabolism in cancer	Human Diseases	Cancers: Overview	PGAM2; FGFR3; NTRK3; PIK3R3; GCK; MAPK3; FGFR1; EGFR; PIK3CA; RET; PDGFRA; PDGFRB; MAP2K2; PIK3CB; KIT; NTRK1; AKT2; PIK3CD; SLC2A1; MTOR; PIK3R2; FGFR2; HIF1A; FLT3; RAF1; PIK3R1; MET; MAPK1; ERBB2; AKT1	30	0.000701

4	MicroRNAs in cancer	Human Diseases	Cancers: Overview	VEGFA; IKBKB; FGFR3; STAT3; PLAU; CDK6; ABCB1; EGFR; PIK3CA; PDGFRA; PDGFRB; MAP2K2; ABL1; PRKCG; ABCC1; MMP16; PRKCE; PAK4; BMPR2; MAPK7; MTOR; PIK3R2; PRKCB; HDAC4; PTGS2; CYP1B1; CASP3; RAF1; PIM1; BCL2; ROCK1; MET; ERBB2; MAPK1	34	0.024695
5	Choline metabolism in cancer	Human Diseases	Cancers: Overview	PLA2G4A; PIK3R3; MAPK3; EGFR; PIK3R2; PDPK1; PDGFRA; PDGFRB; MAP2K2; PRKCG; PIK3CB; PIK3R1; MAPK9; AKT2; PIK3CD; MTOR; PIK3CA; PRKCB; HIF1A; MAPK10; RAF1; AKT1; MAPK1	23	0.039373
**Cancers: Specific types**						
1	Breast cancer	Human Diseases	Cancers: Specific types	PGR; PIK3R2; CDK6; PIK3R3; FGF19; IGF1R; FLT4; MAPK3; FGFR1; IGF1; EGFR; PIK3CA; KIT; MAP2K2; RAF1; PIK3CB; ESR2; PIK3R1; NFKB2; AKT2; PIK3CD; BRAF; ESR1; MTOR; CSNK1A1; NCOA1; FGF4; GSK3B; FGF2; CDK4; FGF1; MAPK1; ERBB2; AKT1	34	1.95E-05
2	Gastric cancer	Human Diseases	Cancers: Specific types	TGFBR1; PIK3R2; PIK3R3; CDK2; FGF19; MAPK3; HGF; ABCB1; RXRG; TGFBR2; EGFR; PIK3CA; MAP2K2; RAF1; PIK3CB; PIK3R1; RXRA; AKT2; PIK3CD; BRAF; MTOR; CSNK1A1; FGFR2; FGF4; RXRB; GSK3B; FGF2; BCL2; FGF1; MET; MAPK1; ERBB2; AKT1	33	0.000106
3	Glioma	Human Diseases	Cancers: Specific types	PIK3R3; IGF1R; CDK6; MAPK3; CAMK2B; IGF1; EGFR; PIK3CA; CAMK2D; PDGFRA; PDGFRB; MAP2K2; PRKCG; PIK3CB; PIK3R1; AKT2; PIK3CD; CAMK2G; BRAF; MTOR; PIK3R2; PRKCB; CAMK2A; RAF1; CDK4; AKT1; MAPK1	27	0.000146
4	Melanoma	Human Diseases	Cancers: Specific types	PIK3R3; FGF19; IGF1R; CDK6; MAPK3; FGFR1; IGF1; EGFR; PIK3CA; PDGFRA; PDGFRB; MAP2K2; PIK3CB; HGF; PIK3R1; AKT2; PIK3CD; BRAF; PIK3R2; FGF4; RAF1; FGF2; CDK4; FGF1; MET; AKT1; MAPK1	27	0.000146
5	Non-small cell lung cancer	Human Diseases	Cancers: Specific types	PIK3R3; JAK3; CDK6; MAPK3; RXRG; EGFR; PIK3CA; PDPK1; MAP2K2; PRKCG; PIK3CB; PIK3R1; STAT3; ALK; RXRA; AKT2; PIK3CD; STAT5B; BRAF; PIK3R2; PRKCB; RXRB; RAF1; CDK4; AKT1; ERBB2; MAPK1	27	0.00015
6	Prostate cancer	Human Diseases	Cancers: Specific types	IKBKB; INSRR; PIK3R3; CDK2; PLAU; IGF1R; MAPK3; FGFR1; MMP3; IGF1; CHUK; EGFR; PIK3CA; PDPK1; PDGFRA; PDGFRB; MAP2K2; RAF1; PIK3CB; PIK3R1; HSP90AB1; AR; AKT2; PIK3CD; HSP90AA1; BRAF; HSP90B1; MTOR; PIK3R2; FGFR2; GSTP1; PLAT; GSK3B; BCL2; MAPK1; ERBB2; AKT1	37	0.000214
7	Acute myeloid leukemia	Human Diseases	Cancers: Specific types	IKBKB; CSF1R; PIK3R3; MAPK3; BRAF; CHUK; PIK3CA; KIT; MAP2K2; PIK3CB; PIK3R1; STAT3; AKT2; PIK3CD; STAT5B; MPO; MTOR; PIK3R2; FLT3; CSF2; RAF1; PIM1; PPARD; AKT1; RARA; MAPK1	26	0.000402
8	Renal cell carcinoma	Human Diseases	Cancers: Specific types	VEGFA; PIK3R2; PIK3R3; PAK2; PAK1; MAPK3; PAK5; HGF; PAK3; MAP2K2; PIK3CB; PIK3R1; PAK4; AKT2; PIK3CD; SLC2A1; BRAF; PIK3CA; HIF1A; RAF1; MET; MAPK1; AKT1	23	0.005725
9	Pancreatic cancer	Human Diseases	Cancers: Specific types	VEGFA; IKBKB; PIK3R3; CDK6; MAPK3; TGFBR1; CHUK; EGFR; PIK3CA; PIK3CB; TGFBR2; PIK3R1; STAT3; JAK1; MAPK9; AKT2; PIK3CD; BRAF; MTOR; PIK3R2; MAPK10; RAF1; CDK4; AKT1; ERBB2; MAPK1	26	0.01107
10	Chronic myeloid leukemia	Human Diseases	Cancers: Specific types	TGFBR1; IKBKB; PIK3R3; CDK6; MAPK3; TGFBR2; CHUK; PIK3CA; MAP2K2; ABL1; PIK3CB; HDAC2; BCR; AKT2; PIK3CD; STAT5B; BRAF; PIK3R2; PIK3R1; RAF1; CDK4; MAPK1; AKT1	23	0.03091
**Neural diseases**						
1	EGFR tyrosine kinase inhibitor resistance	Human Diseases	Drug resistance: Antineoplastic	IL6; VEGFA; MAPK3; FGFR3; PIK3R2; PIK3R3; IGF1R; HGF; IGF1; EGFR; PIK3CA; PDGFRA; PDGFRB; MAP2K2; RAF1; PRKCG; PIK3CB; FGF2; PIK3R1; STAT3; KDR; JAK1; AKT2; PIK3CD; AXL; BRAF; MTOR; EIF4E; FGFR2; PRKCB; JAK2; GSK3B; SRC; BCL2; MET; MAPK1; ERBB2; AKT1	38	7.18E-08
2	Morphine addiction	Human Diseases	Substance dependence	PDE4B; GABRQ; GABRD; PDE1A; OPRM1; PDE2A; PRKACA; PDE3B; PDE4A; PDE11A; GABRE; GABRA3; GABRR3; DRD1; GABRB2; GABRG1; GABRP; PRKCG; GABRA2; CACNA1B; GABRG3; PDE7A; CACNA1A; GABRA1; PDE4D; PDE2A; PDE10A; PDE7B; GABRA5; PRKACB; PDE1B; GABRR1; GABRB1; ADORA1; PRKCB; GABRA4; GABRA6; PDE3A; GABRB3; PDE8A; GABRG2	41	4.76E-07
3	Nicotine addiction	Human Diseases	Substance dependence	GABRQ; GABRD; CHRNA6; GRIA2; GABRE; GABRA3; CHRNB2; GABRR1; GABRR3; GABRB2; GABRG1; GRIN2B; GABRP; GABRA2; CHRNA4; GABRG3; GABRA1; CACNA1B; CACNA1A; GABRA5; GRIN1; GABRB1; GRIN3A; GRIN3B; GABRA4; GABRA6; GABRB3; GABRG2	28	5.81E-07
4	Endocrine resistance	Human Diseases	Drug resistance: Antineoplastic	PRKACA; PIK3R2; PIK3R3; IGF1R; MAPK3; MAPK14; GPER1; EGFR; PIK3CA; PTK2; MAP2K2; PIK3CB; IGF1; PIK3R1; BCL2; MAPK11; MAPK9; AKT2; ESR1; PRKACB; BRAF; PIK3CD; MTOR; ESR2; MAPK10; RAF1; SRC; CDK4; MAPK1; ERBB2; MAPK13; AKT1	32	0.000548
5	Alcoholism	Human Diseases	Substance dependence	PRKACA; HDAC8; DDC; CAMKK2; MAPK3; DRD1; SLC18A2; GRIN2B; DRD2; HDAC2; ADORA2B; NTRK2; SLC6A3; CAMKK1; MAOA; GRIN1; BRAF; GRIN3A; SLC29A1; GRIN3B; HDAC4; MAOB; HDAC7; PKIA; RAF1; ADORA2A; MAPK1	27	0.038304
**Endocrine and metabolic diseases**						
1	Insulin resistance	Human Diseases	Endocrine and metabolic diseases	IL6; IKBKB; NOS3; NR1H2; PRKAG3; PRKAA2; PRKCQ; NR1H3; PRKAG1; RPS6KA1; PRKAG2; PIK3CA; PDPK1; PRKAB1; MTOR; PRKAB2; PTPN1; PIK3CB; PIK3R1; PRKCE; STAT3; PYGL; RPS6KA3; MAPK9; AKT2; PIK3CD; SLC2A1; PRKCD; INSR; PIK3R3; TNF; PIK3R2; PRKCB; SREBF1; PRKAA1; MAPK10; RPS6KA6; GSK3B; AKT1; PPARA	40	8.05E-07

2	Type II diabetes mellitus	Human Diseases	Endocrine and metabolic diseases	IKBKB; PIK3R3; GCK; CACNA1E; MAPK3; PIK3CA; KCNJ11; MTOR; PIK3CB; PIK3R1; PRKCE; MAPK9; CACNA1B; CACNA1A; PIK3CD; PRKCD; INSR; TNF; PIK3R2; CACNA1C; MAPK10; CACNA1G; MAPK1	23	0.000 106
3	AGE-RAGE signaling pathway in diabetic complications	Human Diseases	Endocrine and metabolic diseases	IL6; VEGFA; PLCD1; AGTR1; PIK3R3; MAPK3; TGFBR1; MAPK14; PIK3CA; BCL2; PIK3CB; VCAM1; TGFBR2; PIK3R1; PRKCE; MAPK11; STAT3; SELE; COL1A1; MAPK9; AKT2; PIK3CD; PRKCD; NOS3; ICAM1; TNF; PIK3R2; PRKCB; IL1B; MAPK10; JAK2; CASP3; FN1; STAT5B; PIM1; CDK4; MAPK1; MAPK13; AKT1	39	0.000 132
**Infectious diseases**						
1	Measles	Human Diseases	Infectious diseases: Viral	IL12B; IL2RB; IL6; HSPA8; PIK3R2; CDK6; PIK3R3; CDK2; CSNK2A2; JAK3; IRAK4; CSNK2A1; TBK1; PIK3CA; IFNAR1; FYN; JAK1; PIK3CB; TLR9; PIK3R1; CDK4; STAT3; IKBKE; AKT2; PIK3CD; EIF2AK2; EIF2AK4; TLR4; CSNK2B; IL1B; CHUK; TYK2; TLR7; PRKCQ; IL2; JAK2; TACR1; STAT5B; GSK3B; IRAK1; EIF2AK1; AKT1	42	1.00E -05
2	Epstein-Barr virus infection	Human Diseases	Infectious diseases: Viral	HSPA8; IKBKB; PRKACA; PIK3R3; CDK2; CSNK2A2; JAK3; MAP2K6; RIPK1; MAPK14; CSNK2A1; TBK1; CCNA2; PIK3CA; LYN; SYK; NFKB2; CDK1; PIK3R1; BCL2; MAPK11; STAT3; PIK3CB; JAK1; MAPK9; AKT2; PIK3CD; HDAC2; EIF2AK2; PRKACB; CSNK2B; FCER2; ICAM1; PIK3R2; MAP2K3; HDAC4; CHUK; TYK2; MAPK10; FGR; GSK3B; IRAK1; EIF2AK1; AKT1; MAPK13; EIF2AK4	46	9.90E -05
3	Hepatitis C	Human Diseases	Infectious diseases: Viral	IKBKB; MAPK1; NR1H3; MAPK3; RIPK1; MAPK14; CHUK; TBK1; PIK3CA; PDPK1; IFNAR1; JAK1; RAF1; PIK3CB; PIK3R1; MAPK11; STAT3; RXRA; IKBKE; MAPK9; AKT2; PIK3CD; EIF2AK2; EGFR; EIF2AK4; BRAF; PIK3R3; TNF; PIK3R2; TYK2; MAPK10; GSK3B; EIF2AK1; AKT1; MAPK13; PPARA	36	0.000 166
4	Influenza A	Human Diseases	Infectious diseases: Viral	IL12B; IL6; HSPA8; IKBKB; PIK3R2; PIK3R3; PRSS1; MAP2K6; IRAK4; MAPK3; MAPK14; TBK1; PIK3CA; IFNAR1; MAP2K2; JAK1; RAF1; PRSS2; IL1B; PIK3R1; MAPK11; PIK3CB; PRKCB; IKBKE; MAPK9; AKT2; PIK3CD; EIF2AK2; EIF2AK4; TLR4; ICAM1; TNF; FDPS; MAP2K3; TYK2; TLR7; MAPK10; JAK2; GSK3B; EIF2AK1; MAPK1; PRSS3; MAPK13; AKT1	44	0.000 199
5	Kaposi sarcoma-associated herpesvirus infection	Human Diseases	Infectious diseases: Viral	IL6; BECN1; IKBKB; PIK3R2; MAPKAPK2; PIK3R3; MAP2K6; PTGS2; CDK6; MAPK3; CSF2; VEGFA; MAPK14; CHUK; TBK1; PIK3CA; IFNAR1; MAP2K2; JAK1; LYN; RAF1; SYK; SRC; PIK3R1; MAPK11; STAT3; PIK3CB; IKBKE; MAPK9; AKT2; PIK3CD; EIF2AK2; ICAM1; PIK3C3; MTOR; PIK3CG; TYK2; HIF1A; MAPK10; JAK2; CASP3; HCK; GSK3B; FGF2; CDK4; MAPK1; MAPK13; AKT1	48	0.000 22
6	Hepatitis B	Human Diseases	Infectious diseases: Viral	IL6; TGFBR1; IKBKB; PIK3R2; PIK3R3; CDK2; CDK6; MAPK3; CHUK; TBK1; CCNA2; PIK3CA; IFNAR1; MAP2K2; JAK1; PRKCG; PIK3CB; PIK3R1; BCL2; STAT3; IKBKE; MAPK9; AKT2; PIK3CD; STAT5B; TLR4; PTK2B; TNF; MAP3K1; PRKCB; MAPK10; CASP3; RAF1; SRC; CDK4; MAPK1; AKT1	37	0.013 04
7	Toxoplasmosis	Human Diseases	Infectious diseases: Parasitic	IL12B; HSPA8; NOS2; MAP2K6; XIAP; IRAK4; MAPK3; MAPK14; CHUK; PIK3CG; PDPK1; ALOX5; IKBKB; IRAK1; MAPK11; STAT3; PPIF; JAK1; MAPK9; AKT2; TLR4; TNF; MAP2K3; TYK2; MAPK10; JAK2; CASP3; BCL2; AKT1; MAPK13; MAPK1	31	0.023 177
8	African trypanosomiasis	Human Diseases	Infectious diseases: Parasitic	IL12B; PRKCB; HBB; IL6; IL1B; TLR9; PRKCG; VCAM1; ICAM1; TNF; SELE	11	0.035 695
**Cardiovascular diseases**						
1	Fluid shear stress and atherosclerosis	Human Diseases	Cardiovascular diseases	ACVR1; VEGFA; IKBKB; CDH5; PIK3R2; PIK3R3; PRKAA2; MAP2K6; GSTM1; SELE; MAPK14; CHUK; PIK3CA; VCAM1; PTK2; GSTA2; PIK3CB; GSTM4; BMPR1A; PIK3R1; MAPK11; HSP90AB1; KDR; NQO1; MAPK9; AKT2; PIK3CD; BMPR2; CTSL; NOS3; MAPK7; ICAM1; HSP90B1; TNF; ASS1; MAP2K5; IL1B; BMPR1B; GSTP1; PRKAA1; MAPK10; PLAT; GSTA1; SRC; BCL2; HSP90AA1; MAPK13; AKT1	48	0.004 032
2	Hypertrophic cardiomyopathy (HCM)	Human Diseases	Cardiovascular diseases	ITGA4; IL6; PRKAB1; PRKAA2; PRKAG1; CACNB2; IGF1; PRKAG2; ACE; PRKAG3; CACNA1S; CACNG1; CACNA1F; TNNC1; CACNB3; CACNB1; PRKAB2; TNF; CACNB4; PRKAA1; CACNA2D2; CACNA2D1; CACNA1C	23	0.023 177
**Organismal Systems**						
**Endocrine system**						
1	Progesterone-mediated oocyte maturation	Organismal Systems	Endocrine system	PRKACA; STK10; PDE3B; PIK3R3; CDK2; HSP90AB1; IGF1R; MAPK3; RPS6KA1; MAPK14; CCNA2; PIK3CA; PKMYT1; PIK3CB; CDK1; IGF1; PIK3R1; MAPK11; PGR; RPS6KA3; MAPK9; AKT2; PIK3CD; HSP90AA1; PRKACB; BRAF; PIK3R2; PLK1; MAPK10; AURKA; RPS6KA6; RAF1; MAPK1; MAPK13; AKT1	35	7.16E -08

2	Insulin signaling pathway	Organismal Systems	Endocrine system	AKT2; PRKAG3; IKBKB; PRKACA; MKNK1; PIK3R2; PRKAB1; PRKAA2; GCK; PRKAG1; MAPK3; PRKAG2; PIK3CA; FBP1; PDPK1; MKNK2; MAP2K2; PDE3B; RAF1; PTPN1; PIK3CB; PIK3R1; PYGL; MAPK9; PRKCI; PHKG1; PIK3CD; PRKACB; INSR; PRKAB2; BRAF; PIK3R3; MTOR; EIF4E; PRKAR1A; SREBF1; PRKAA1; MAPK10; GSK3B; MAPK1; AKT1	41	3.90E-06
3	Oxytocin signaling pathway	Organismal Systems	Endocrine system	PRKACA; PLA2G4A; CACNG1; PRKAG3; PRKAA2; CAMK1; CAMK1G; CAMK1D; PRKAG1; CAMKK2; MAPK3; MYLK4; CAMK2B; EGFR; PRKAG2; MAP2K5; CAMK2D; PRKAB1; ROCK2; MAP2K2; CACNA1S; PRKAB2; PRKCG; CACNA1F; MYLK2; CACNA2D1; MYLK3; OXTR; CACNB3; CACNB1; MYLK; CAMK2G; PRKACB; NOS3; MAPK7; CACNB2; CACNB4; PIK3CG; PRKCB; PTGS2; PRKAA1; CAMK2A; KCNJ4; CACNA2D2; RAF1; SRC; ROCK1; GUCY1A2; CACNA1C; MAPK1	50	0.000141
4	Prolactin signaling pathway	Organismal Systems	Endocrine system	PIK3R3; GCK; MAPK3; MAPK14; PIK3CA; MAP2K2; RAF1; PIK3CB; ESR2; PIK3R1; MAPK11; STAT3; PRLR; MAPK9; AKT2; ESR1; STAT5B; PIK3CD; TNFRSF11A; PIK3R2; MAPK10; JAK2; GSK3B; SRC; AKT1; MAPK13; MAPK1	27	0.000227
5	GnRH signaling pathway	Organismal Systems	Endocrine system	CAMK2A; PRKACA; PLA2G4A; MAP3K4; MAP2K6; MAPK3; CAMK2B; MAPK14; EGFR; MAP3K1; CAMK2D; MAP2K2; MAP3K3; CACNA1S; CACNA1F; MAPK11; PRKCB; MAPK9; CAMK2G; PRKCD; MAPK7; PTK2B; MAP2K3; GNRHR; CACNA1C; MAPK10; RAF1; SRC; MAP3K2; MAPK1; MAPK13; PRKACB	32	0.000228
6	Thyroid hormone signaling pathway	Organismal Systems	Endocrine system	THRB; SLC9A1; PRKACA; PIK3R2; PIK3R3; MAPK3; RXRG; PIK3CA; PDPK1; MAP2K2; RAF1; PRKCG; PIK3CB; PIK3R1; RXRA; AKT2; ESR1; SLC2A1; PRKACB; PIK3CD; MTOR; NCOA2; PRKCB; NCOA1; HIF1A; THRA; RXRB; PLCD1; ATP1A1; GSK3B; SRC; HDAC2; MAPK1; AKT1	34	0.001377
7	Adipocytokine signaling pathway	Organismal Systems	Endocrine system	MAPK10; IKBKB; PRKAB1; PRKAA2; PRKCQ; PRKAG1; CAMKK2; RXRG; CHUK; PRKAG2; PRKAG3; MTOR; PRKAB2; STAT3; RXRA; MAPK9; AKT2; SLC2A1; PPARA; TNF; PRKAA1; RXRB; JAK2; AKT1	24	0.005189
8	Estrogen signaling pathway	Organismal Systems	Endocrine system	TFF1; HSPA8; PRKACA; PGR; PIK3R2; CTSD; PIK3R3; MAPK3; OPRM1; GPER1; EGFR; PIK3CA; MAP2K2; PIK3CB; PIK3R1; HSP90AB1; AKT2; ESR1; PRKACB; HSP90AA1; PRKCD; NOS3; PIK3CD; HSP90B1; ESR2; NCOA1; NCOA2; RAF1; SRC; BCL2; AKT1; RARA; MAPK1	33	0.005888
9	Regulation of lipolysis in adipocytes	Organismal Systems	Endocrine system	ADRB1; PIK3R3; PRKG2; PIK3CA; PRKACA; PIK3R2; PDE3B; AKT2; ADRB2; PIK3CD; PRKACB; PIK3CB; INSR; ADORA1; ADRB3; AKT1; PIK3R1; PTGS2	18	0.010361
10	Relaxin signaling pathway	Organismal Systems	Endocrine system	VEGFA; NOS2; PRKACA; TGFBR2; PIK3R3; MAPK3; TGFBR1; MAPK14; EGFR; PIK3CA; MAP2K2; PIK3CB; EDNRB; PIK3R1; MAPK11; COL1A1; MAPK9; AKT2; PIK3CD; NOS1; PRKACB; NOS3; PIK3R2; MAPK10; MMP1; RAF1; SRC; MMP13; MAPK1; MAPK13; AKT1	31	0.010361
11	Renin secretion	Organismal Systems	Endocrine system	ADRB1; KCNMA1; PRKG2; REN; ACE; PDE1B; PDE3A; PRKACA; AGTR1; PDE1A; PDE3B; ADRB2; EDNRA; CACNA1S; PRKACB; CACNA1F; CTSB; ADRB3; GUCY1A2; CACNA1C; ADORA1	21	0.01558
12	Insulin secretion	Organismal Systems	Endocrine system	PRKACA; KCNMB3; GCK; CAMK2B; KCNMA1; KCNN3; CAMK2D; KCNJ11; ATP1A1; CACNA1S; PRKCG; CACNA1F; CHRM3; KCNN4; SLC2A1; KCNMB4; CAMK2G; PRKACB; KCNN1; PRKCB; KCNN2; CAMK2A; KCNMB1; CACNA1C	24	0.035128
13	Ovarian steroidogenesis	Organismal Systems	Endocrine system	IGF1; PTGS2; HSD3B1; CYP1B1; PRKACA; PLA2G4A; CYP19A1; HSD17B2; ALOX5; AKR1C3; IGF1R; PRKACB; CYP1A1; INSR; HSD17B1	15	0.048831
**Immune system**						
1	Fc epsilon RI signaling pathway	Organismal Systems	Immune system	PLA2G4A; PIK3R3; MAP2K6; MAPK3; CSF2; MAPK14; ALOX5; PIK3CB; PIK3CA; PDPK1; FYN; MAP2K2; LYN; SYK; PIK3R1; MAPK11; BTK; MAPK9; AKT2; PIK3CD; TNF; PIK3R2; MAP2K3; MAPK10; RAF1; AKT1; MAPK13; MAPK1	28	0.000216
2	Toll-like receptor signaling pathway	Organismal Systems	Immune system	IL12B; IL6; IKBKB; PIK3R2; PIK3R3; MAP2K6; IRAK4; MAPK3; RIPK1; MAPK14; CHUK; TBK1; PIK3CA; CTSK; IFNAR1; MAP2K2; PIK3CB; TLR9; PIK3R1; MAPK11; IKBKE; MAPK9; AKT2; PIK3CD; TLR4; TNF; IL1B; MAP2K3; TLR7; MAPK10; IRAK1; MAPK1; MAPK13; AKT1	34	0.000275
3	C-type lectin receptor signaling pathway	Organismal Systems	Immune system	IL12B; IL6; IKBKB; PIK3R2; MAPKAPK2; PIK3R3; PAK1; MAPK3; MAPK14; CHUK; PIK3CA; IL2; SYK; NFKB2; PIK3R1; MAPK11; PIK3CB; IKBKE; MAPK9; AKT2; PIK3CD; PRKCD; PLK3; TNF; IL1B; PTGS2; MAPK10; RAF1; SRC; AKT1; MAPK13; MAPK1	32	0.000801
4	T cell receptor signaling pathway	Organismal Systems	Immune system	IKBKB; PIK3R2; PIK3R3; PAK2; PRKCQ; PAK1; MAPK3; CSF2; MAPK14; PAK5; PAK3; PDPK1; FYN; MAP2K2; RAF1; PIK3CB; PIK3R1; MAPK11; PAK4; ITK; IL2; MAPK9; AKT2; PIK3CD; TEC; TNF; PIK3CA; CHUK; LCK; ZAP70; GSK3B; CDK4; MAPK1; MAPK13; AKT1	35	0.000801
5	Platelet activation	Organismal Systems	Immune system	AKT2; P2RY12; PRKACA; PLA2G4A; PIK3R2; PIK3R3; GP9; MAPK3; MYLK4; ROCK2; MAPK14; PRKG2; PIK3CB; PIK3CA; FYN; LYN; F2R; SYK; MYLK2; MYLK3; PIK3R1; MAPK11; BTK; COL1A1; PRKCI; PIK3CD; MYLK; PRKACB; NOS3; PIK3CG; SRC; ROCK1; MAPK1; GUCY1A2; MAPK13; AKT1	36	0.002098
6	Th17 cell differentiation	Organismal Systems	Immune system	IL2RB; RXRB; IL17A; TGFBR1; IKBKB; PRKCQ; JAK3; MAPK3; RXRG; TGFBR2; MAPK14; CHUK; IL2; AHR; MAPK11; STAT3; HSP90AB1; RXRA; JAK1; MAPK9;	34	0.004017

				HSP90AA1; STAT5B; IL6; MTOR; IL1B; TYK2; HIF1A; LCK; MAPK10; JAK2; MAPK13; ZAP70; RARA; MAPK1		
7	IL-17 signaling pathway	Organismal Systems	Immune system	MAPK4; IL6; IL17A; IKBKB; MAPK3; CSF2; MMP3; MAPK14; CHUK; TBK1; MMP13; MAPK15; MAPK11; HSP90AB1; IKBKE; MAPK9; HSP90AA1; MAPK7; HSP90B1; TNF; IL1B; PTGS2; MAPK10; CASP3; MMP1; GSK3B; MAPK13; MAPK1	28	0.004483
8	Chemokine signaling pathway	Organismal Systems	Immune system	IKBKB; PRKACA; PIK3R2; MAPK1; JAK3; PAK1; MAPK3; GSK3A; CHUK; PIK3CG; PTK2; ROCK2; LYN; RAF1; PIK3CB; PIK3R1; STAT3; ITK; AKT2; PIK3CD; FGR; PRKCD; STAT5B; BRAF; PTK2B; PIK3CA; PRKCB; PF4; JAK2; HCK; GSK3B; SRC; PIK3R3; ROCK1; AKT1; PRKACB	36	0.006909
9	Fc gamma R-mediated phagocytosis	Organismal Systems	Immune system	LIMK2; PLA2G4A; LIMK1; PIK3R3; PAK1; MAPK3; PIK3CA; LYN; PRKCG; SYK; PIK3R1; PRKCE; PIK3CB; AKT2; PIK3CD; PLA2G6; PRKCD; PIK3R2; PRKCB; ARPC3; HCK; RAF1; MAPK1; AKT1	24	0.007233
**Nervous system**						
1	Neurotrophin signaling pathway	Organismal Systems	Nervous system	CAMK2A; IKBKB; NTRK3; MAPKAPK2; MAPK1; IRAK4; MAPK3; CAMK2B; MAPK14; MAP2K5; CAMK2D; MAP3K3; PDPK1; RPS6KA1; MAP2K2; ABL1; RAF1; PIK3CB; RIPK2; PIK3R1; BCL2; NTRK2; MAPK11; NTRK1; RPS6KA3; MAPK9; PIK3R2; AKT2; PIK3CD; CAMK2G; PRKCD; MAPK7; BRAF; PIK3R3; MAP3K1; PIK3CA; MAPK10; RPS6KA6; GSK3B; IRAK1; IRAK3; AKT1; MAPK13; MATK	44	6.90E-07
2	Serotonergic synapse	Organismal Systems	Nervous system	PRKACA; PLA2G4A; HTR3B; DDC; HTR3D; HTR2B; MAPK3; SLC18A2; HTR3C; HTR6; HTR5A; HTR1B; ALOX5; HTR3E; CACNA1S; HTR1D; PRKCG; HTR2C; HTR7; HTR2A; CYP2C9; KCND2; SLC6A4; GABRB2; HTR1A; CACNA1F; CACNA1B; CACNA1A; HTR1E; HTR1F; PRKACB; MAOA; BRAF; GABRB1; PRKCB; HTR3A; MAOB; PTGS2; HTR4; KCNN2; GABRB3; CASP3; RAF1; CACNA1C; MAPK1	45	1.73E-06
3	Cholinergic synapse	Organismal Systems	Nervous system	KCNQ2; KCNQ4; CHRNB4; CHRM4; PRKACA; PIK3R2; PIK3R3; CHRNB2; CHRNA6; MAPK3; CAMK2B; PIK3CG; CAMK2D; FYN; CACNA1S; PRKCG; CACNA1F; CHRNA4; PIK3R1; CHRM2; CHRM1; PIK3CB; CACNA1B; CACNA1A; AKT2; CHRM3; PIK3CD; CAMK2G; PRKACB; CHRM5; PIK3CA; KCNQ1; PRKCB; CHRNA3; CACNA1C; CAMK2A; JAK2; KCNJ4; ACHE; BCL2; MAPK1; AKT1	42	2.13E-06
4	Dopaminergic synapse	Organismal Systems	Nervous system	DRD5; MAPK10; GRIA2; PRKACA; DDC; SCN1A; DRD1; CAMK2B; MAPK14; DRD3; CAMK2D; GRIN2B; DRD2; SLC18A2; PRKCG; MAPK11; SLC6A3; GSK3A; MAPK9; CACNA1B; CACNA1A; AKT2; CAMK2G; PRKACB; MAOA; DRD4; PRKCB; MAOB; CACNA1C; CAMK2A; COMT; GSK3B; MAPK13; AKT1	34	0.006909
5	GABAergic synapse	Organismal Systems	Nervous system	GABRQ; GABRD; PRKACA; PLCL1; GABRE; GABRA3; GABRR3; GABRB2; GABRG1; GABRP; CACNA1S; PRKCG; CACNA1F; GABRA2; GABRG3; GABRA1; CACNA1B; CACNA1A; GABRA5; PRKACB; GABRR1; GABRB1; PRKCB; GABRA4; GABRA6; CACNA1C; GABRB3; SRC; GABRG2	29	0.007268
**Circulatory system**						
1	Vascular smooth muscle contraction	Organismal Systems	Circulatory system	PRKACA; AGTR1; PLA2G4A; ADRA1D; MAPK1; PRKCQ; KCNMB3; MAPK3; MYLK4; ROCK2; KCNMA1; PLA2G1B; ADRA1A; MAP2K2; CACNA1S; PRKCG; CACNA1F; MYLK2; MYLK3; PRKCE; ADORA2B; AVPR1A; PLA2G6; KCNMB4; PRKCD; ADRA1B; BRAF; PLA2G2A; PRKCB; EDNRA; MYLK; PLA2G2E; CACNA1C; KCNMB1; RAF1; ROCK1; ADORA2A; GUCY1A2; PRKACB	39	0.000132
2	Adrenergic signaling in cardiomyocytes	Organismal Systems	Circulatory system	SCN5A; MAPK11; PRKACA; AGTR1; MAPK1; SLC9A1; MAPK3; CAMK2B; MAPK14; CACNB2; PIK3CG; CAMK2D; ATP1A1; ADRA1A; CACNA1S; CACNG1; CACNA1F; TNNC1; KCNQ1; CACNB3; CACNB1; ADRB2; AKT2; CAMK2G; PRKACB; ADRA1B; CACNB4; ADRB1; CACNA1C; CAMK2A; CACNA2D2; CACNA2D1; BCL2; AKT1; MAPK13; ADRA1D	36	0.005394
**Digestive system**						
1	Salivary secretion	Organismal Systems	Digestive system	ADRB1; KCNMA1; PRKG2; SLC9A1; PRKCB; ATP1A1; GUCY1A2; ADRA1A; LYZ; CHRM3; KCNN4; NOS1; PRKACB; ADRA1B; PRKACA; ADRB3; ADRA1D; ADRB2; PRKCG	19	0.020551
2	Gastric acid secretion	Organismal Systems	Digestive system	KCNQ1; PRKCB; HRH2; CAMK2D; MYLK; ATP4B; PRKACA; CAMK2A; CHRM3; CFTR; MYLK2; SLC9A1; CAMK2G; PRKACB; ATP1A1; ATP4A; CA2; MYLK3; MYLK4; CAMK2B; PRKCG	21	0.030471
**Aging**						
1	Longevity regulating pathway - multiple species	Organismal Systems	Aging	HSPA8; PRKACA; PRKAG3; PRKAA2; IGF1R; PRKAG1; IGF1; PRKAG2; PIK3CA; PRKAB1; PRKAB2; PIK3CB; HDAC2; AKT2; PIK3CD; PRKACB; INSR; PIK3R3; MTOR; PIK3R2; PRKAA1; SOD1; PIK3R1; AKT1	24	0.000167
2	Longevity regulating pathway	Organismal Systems	Aging	PRKACA; PIK3R2; PRKAG3; PRKAA2; IGF1R; PRKAG1; CAMKK2; IGF1; PRKAG2; PIK3CA; PRKAB1; PRKAB2; PIK3CB; PIK3R1; AKT2; PIK3CD; PRKACB; INSR; PIK3R3; MTOR; EIF4E; ULK1; PPARG; PRKAA1; AKT1	25	0.001976

**Others**						
1	Axon guidance	Organismal Systems	Development	LIMK2; GSK3B; EPHB2; EPHA4; LIMK1; PIK3R3; PAK2; PAK1; MAPK3; CAMK2B; PAK5; PAK3; CAMK2D; PTK2; FYN; ROCK2; ABL1; PIK3CB; EPHA2; SRC; EPHB4; PIK3R1; PAK4; PIK3R2; EPHA5; PIK3CD; EPHA1; CAMK2G; ROCK1; EPHB1; PIK3CA; BMPR1B; EPHA7; CAMK2A; FES; EPHA3; RAF1; EPHA6; BMPR2; MET; MAPK1; NRP1; EPHA8; EPHB6	44	2.50E-07
2	Inflammatory mediator regulation of TRP channels	Organismal Systems	Sensory system	CAMK2A; MAPK11; ASIC1; PRKACA; PLA2G4A; PIK3R2; PIK3R3; PRKCQ; MAP2K6; HTR2B; CAMK2B; MAPK14; PIK3CB; PIK3CA; CAMK2D; PRKCG; HTR2C; HTR2A; IGF1; PIK3R1; PRKCE; BDKRB1; NTRK1; PRKCB; MAPK9; TRPA1; PIK3CD; PLA2G6; CAMK2G; PRKCD; HRH1; MAP2K3; IL1B; MAPK10; SRC; MAPK13; PRKACB	37	0.000111
3	Taste transduction	Organismal Systems	Sensory system	PRKACA; HTR3B; GABRA3; HTR3D; HTR3C; HTR1B; HTR3E; HTR1D; GABRA2; HTR1A; CACNA1A; GABRA1; PDE1A; HTR1E; CHRM3; HTR1F; PDE1B; GABRA5; PRKACB; SCN2A; HTR3A; GABRA4; GABRA6; CACNA1C	24	0.000809
4	Olfactory transduction	Organismal Systems	Sensory system	PRKG2; CAMK2D; PDE2A; PRKACA; PDE2A; CAMK2A; PDE1B; CAMK2G; PRKACB; PDE1A; CAMK2B	11	0.005725
5	Aldosterone-regulated sodium reabsorption	Organismal Systems	Excretory system	IGF1; PRKCB; ATP1A1; PIK3CA; PDPK1; PIK3R2; PIK3CD; NR3C2; SGK1; PRKCG; PIK3CB; INSR; PIK3R3; MAPK3; PIK3R1; MAPK1	16	0.020551
6	Circadian rhythm	Organismal Systems	Environmental adaptation	PRKAG2; NR1D1; PRKAA1; PRKAG3; PRKAB1; PRKAA2; PRKAB2; PRKAG1	8	0.024595
7	Osteoclast differentiation	Organismal Systems	Development	TGFBR1; IKBKB; CSF1R; PIK3R3; MAP2K6; MAPK3; TGFBR2; MAPK14; CHUK; PIK3CB; PIK3CA; CTSK; IFNAR1; FYN; SYK; NFKB2; PIK3R1; MAPK11; BTK; JAK1; MAPK9; AKT2; PIK3CD; TEC; TNF; TNFRSF11A; PIK3R2; PPARG; TYK2; IL1B; LCK; MAPK10; MAPK1; MAPK13; AKT1	35	0.033585
**Cellular processes**						
1	Autophagy - animal	Cellular Processes	Transport and catabolism	BECN1; PRKACA; CTSD; MAPK1; PRKAA2; PRKCQ; IGF1R; CTSL; CAMKK2; MAPK3; ULK2; PIK3CA; PDPK1; ERN1; MAP2K2; PIK3CB; CTSB; PIK3R1; MAPK9; AKT2; PIK3CD; PRKACB; EIF2AK4; PIK3R3; PIK3C3; MTOR; PIK3R2; DAPK2; ULK1; HIF1A; DAPK3; PRKAA1; MAPK10; RAF1; BCL2; AKT1; DAPK1; PRKCD	38	1.51E-07
2	Signaling pathways regulating pluripotency of stem cells	Cellular Processes	Cellular community - eukaryotes	ACVR1; FGFR3; PIK3R3; JAK3; IGF1R; MAPK3; ACVR1B; FGFR1; ESRRB; MAPK14; PIK3CA; MAP2K2; RAF1; PIK3CB; BMPR1A; PIK3R1; MAPK11; STAT3; IGF1; JAK1; AKT2; PIK3CD; BMPR2; FGFR4; PIK3R2; FGFR2; BMPR1B; JAK2; GSK3B; FGF2; MAPK1; MAPK13; AKT1	33	4.76E-07
3	Regulation of actin cytoskeleton	Cellular Processes	Cell motility	LIMK2; SLC9A1; CHRM4; BDKRB1; INSRR; FGFR3; LIMK1; PIK3R3; PAK2; FGF19; PAK1; MAPK3; MYLK4; ROCK2; FGFR1; PAK5; FGFR4; PAK3; PTK2; PDGFRA; PDGFRB; MAP2K2; ITGA4; F2R; PIK3CB; MYLK2; FGF2; MYLK3; F2; PIK3R1; CHRM2; CHRM1; PAK4; PIK3R2; CHRM3; PIK3CD; MYLK; EGFR; CHRM5; BRAF; PIP4K2B; PIK3CA; FGFR2; ARPC3; FGF4; PIP4K2C; RAF1; SRC; FGF1; ROCK1; MYH14; FN1; MAPK1	53	1.68E-05
4	Focal adhesion	Cellular Processes	Cellular community - eukaryotes	ITGA4; VEGFA; MAPK3; PIK3R2; PIK3R3; PAK2; FLT4; IGF1R; XIAP; PAK1; FLT1; HGF; MYLK4; ROCK2; IGF1; PAK5; EGFR; PAK3; PDPK1; PTK2; PDGFRA; FYN; PDGFRB; GSK3B; PRKCG; PIK3CB; MYLK2; MYLK3; PIK3R1; PAK4; KDR; COL1A1; MAPK9; AKT2; PIK3CD; MYLK; BRAF; ROCK1; PIK3CA; PRKCB; MAPK10; RAF1; SRC; BCL2; MET; MAPK1; ERBB2; FN1; AKT1	49	0.000202
5	Adherens junction	Cellular Processes	Cellular community - eukaryotes	CSNK2A1; EGFR; TGFBR1; FER; MAPK3; CSNK2B; FYN; CSNK2A2; IGF1R; SRC; FGFR1; INSR; PTPN1; MET; MAPK1; ERBB2; YES1; TGFBR2	18	0.032955
6	Gap junction	Cellular Processes	Cellular community - eukaryotes	PRKACA; HTR2B; MAPK3; DRD1; PRKG2; EGFR; MAP2K5; PDGFRA; DRD2; PDGFRB; MAP2K2; PRKCG; HTR2C; HTR2A; CDK1; TUBB; PRKACB; MAPK7; ADRB1; PRKCB; TUBA1A; GJA1; RAF1; SRC; MAP3K2; GUCY1A2; MAPK1	27	0.038304

Pathways Enriched in Targets of Dual-modulators (p-value < 0.05)

**Signal transduction**						
**No.**	**Pathway**	**Class**	**Subclass**	**Targets**	**No. Targets**	**p-value**

1	Calcium signaling pathway	Environmental Information Processing	Signal transduction	PRKCA; DRD5; CYSLTR2; NOS2; HTR7; AGTR1; CXCR4; CHRM5; TNNC2; HTR2B; DRD1; CACNA1H; HTR6; EGFR; HTR5A; RYR1; PTGER1; ADRA1A; PDGFRB; CACNA1S; F2R; CACNA1D; PTGER3; CACNA1F; PDE1A; HTR2A; TNNC1; HRH2; ADORA2B; CHRM1; HTR2C; CAMK4; CACNA1B; CACNA1A; CHRM3; PDE1B; ADRB2; ADRA1B; NOS3; ADRB3; PTK2B; CACNA1I; HRH1; ADRB1; PRKCB; GRIN2C; HTR4; CACNA1C; ITPR3; PLCD1; CHRM2; SLC8A1; PRKACA; ADORA2A; LHCGR; CACNA1G; ADRA1D	57	3.73E-11
2	cAMP signaling pathway	Environmental Information Processing	Signal transduction	PDE4B; DRD5; MAPK8; GRIA2; PRKACA; PDE4A; MAP2K1; MAPK3; DRD1; HTR6; PIK3CA; HTR1B; AKT2; DRD2; CACNA1S; HTR1D; CACNA1D; PTGER3; CACNA1F; GRIA4; ADORA2A; CHRM1; HTR1A; GRIA1; CAMK4; PDE4D; HTR1E; ADRB2; HTR1F; PPARA; GRIA3; PIK3CD; ADORA1; ADRB1; PDE4C; GRIN2C; PDE3A; HTR4; CACNA1C; MAPK10; F2R; RAF1; CHRM2; AKT1	44	0.000179
3	cGMP-PKG signaling pathway	Environmental Information Processing	Signal transduction	PDE5A; ADRA2B; ADORA3; ADRA2C; AGTR1; INSR; MAP2K1; MAPK3; KCNMA1; ADRA1A; CACNA1S; CACNA1D; CACNA1F; ADRA2A; OPRD1; INS; NPPB; AKT2; ADRB2; KCNMB2; ADRA1B; NOS3; ADRB3; ADORA1; ADRB1; PDE3A; CACNA1C; ITPR3; RAF1; SLC8A1; ADRA1D; GUCY1A2; AKT1	33	0.000775
4	VEGF signaling pathway	Environmental Information Processing	Signal transduction	MAPK14; PRKCB; VEGFA; PTGS2; PRKCA; PIK3CA; PTK2; AKT2; PIK3CD; RAF1; SRC; NOS3; MAP2K1; HSPB1; MAPK3; AKT1	16	0.012364
5	HIF-1 signaling pathway	Environmental Information Processing	Signal transduction	PRKCA; IL6; VEGFA; NOS2; INSR; MAP2K1; IGF1R; FLT1; MAPK3; EGFR; PIK3CA; INS; SERPINE1; EGF; HK1; AKT2; PIK3CD; TLR4; NOS3; PRKCB; HIF1A; PGK1; BCL2; AKT1	24	0.01916
6	MAPK signaling pathway	Environmental Information Processing	Signal transduction	PRKCA; HSPB1; VEGFA; IKBKB; PRKACA; MAP2K1; IGF1R; FLT1; MAPK8IP1; MAPK3; CACNA1H; MAPK14; EGFR; CACNB2; NGF; PDGFRB; CACNA1S; CACNG1; CACNA1D; CACNB4; CACNA1F; INS; MAPT; EGF; CACNA1B; CACNA1A; CACNB3; CACNB1; AKT2; INSR; MAPK8; TNF; CACNA1I; PRKCB; IL1B; CACNA1C; MAPK10; CACNA2D3; CACNA2D2; RAF1; CACNA2D1; CACNA1G; AKT1	43	0.023873
7	Ras signaling pathway	Environmental Information Processing	Signal transduction	PRKCA; VEGFA; IKBKB; HTR7; MAP2K1; IGF1R; FLT1; MAPK3; MAPK8; EGFR; PIK3CA; PLA2G2D; NGF; PDGFRB; PRKACA; EGF; INS; AKT2; PIK3CD; INSR; PLA2G2A; PRKCB; MAPK10; ZAP70; RAF1; BCL2L1; PLA2G1B; PLA2G2E; AKT1	29	0.040438
8	Rap1 signaling pathway	Environmental Information Processing	Signal transduction	PRKCA; VEGFA; PRKCB; MAP2K1; IGF1R; FLT1; F2RL3; MAPK14; EGFR; PIK3CA; NGF; DRD2; PDGFRB; ITGB3; F2R; MAPK3; ADORA2B; EGF; INS; AKT2; PIK3CD; INSR; ACTB; RAF1; SRC; ADORA2A; AKT1	27	0.048829
**Human diseases**						
**Cancers: overview**						
1	Pathways in cancer	Human Diseases	Cancers: Overview	IL12B; PRKCA; MAPK8; IL6; VEGFA; NOS2; MAPK3; AGTR1; CXCR4; CDK2; CDK6; MAP2K1; IGF1R; CYCS; F2RL3; RXRG; HDAC1; EGFR; PIK3CA; EGF; PTGER1; MAPK10; CSF2RB; SMO; PDGFRB; IKBKB; RAF1; BIRC5; PRKACA; ESR2; PTK2; HDAC2; BCL2; HSP90AB1; SKP1; BMP4; ALK; RXRA; AR; AKT2; ESR1; HSP90AA1; RARB; PIK3CD; PTGER3; PTGS2; PRKCB; PPARG; NCOA1; HIF1A; MMP2; RXRB; F2R; GSK3B; PIM1; PPARD; BCL2L1; GSTP1; IL23A; CSF2RA; RARA; AKT1	62	0.018902
2	Transcriptional misregulation in cancer	Human Diseases	Cancers: Overview	PPARG; IL6; RXRA; ATM; RXRB; PTK2; RARA; CEBPB; FLT1; PLAU; IGF1R; BCL2L1; GRIA3; MPO; MMP3; RXRG; HDAC2; HDAC1	18	0.036238
**Cancers: specific types**						
1	Non-small cell lung cancer	Human Diseases	Cancers: Specific types	PRKCB; EGFR; PIK3CA; ALK; RXRA; PRKCA; RXRB; AKT2; MAP2K1; PIK3CD; CDK6; RAF1; FHIT; EGF; RARB; MAPK3; RXRG; AKT1	18	0.004115

2	Small cell lung cancer	Human Diseases	Cancers: Specific types	PTK2; PTGS2; IKBKB; RXRA; NOS2; PIK3CA; RXRB; AKT2; FHIT; PIK3CD; CDK2; CYCS; CDK6; BCL2; BCL2L1; RARB; RXRG; AKT1	18	0.019 16
3	Prostate cancer	Human Diseases	Cancers: Specific types	IKBKB; CDK2; PLAU; IGF1R; MAPK3; MMP3; EGFR; PIK3CA; PDGFRB; GSK3B; HSP90AB1; INS; AR; AKT2; PIK3CD; HSP90AA1; EGF; GSTP1; RAF1; BCL2; MAP2K1; AKT1	22	0.024 46
**Cardiovascular diseases**						
1	Dilated cardiomyop athy (DCM)	Human Diseases	Cardiovascul ar diseases	ADRB1; ACTB; CACNB2; CACNA2D2; PRKACA; CACNA1C; CACNB3; CACNB1; CACNA2D3; ITGB3; CACNA1S; CACNG1; CACNA1D; SLC8A1; CACNA1F; CACNA2D1; TNNC1; TNF; CACNB4	19	0.001 977
2	Arrhythmo genic right ventricular cardiomyop athy (ARVC)	Human Diseases	Cardiovascul ar diseases	ACTB; CACNB2; CACNA2D2; CACNA1C; CACNB3; CACNB1; CACNA2D3; ITGB3; CACNA1S; CACNG1; CACNA1D; SLC8A1; CACNA1F; CACNA2D1; CACNB4	15	0.002 841
3	Hypertroph ic cardiomyop athy (HCM)	Human Diseases	Cardiovascul ar diseases	ACTB; IL6; CACNB2; CACNA2D2; ACE; CACNA1C; CACNB3; CACNB1; CACNA2D3; ITGB3; CACNA1S; CACNG1; CACNA1D; SLC8A1; CACNA1F; CACNA2D1; TNNC1; TNF; CACNB4	19	0.003 716
**Endocrine and metabolic diseases**						
1	Type II diabetes mellitus	Human Diseases	Endocrine and metabolic diseases	MAPK10; HK1; PIK3CA; KCNJ11; IKBKB; CACNA1C; CACNA1A; PIK3CD; CACNA1B; CACNA1D; INSR; MAPK3; INS; TNF; CACNA1G; MAPK8	16	0.001 867
2	Insulin resistance	Human Diseases	Endocrine and metabolic diseases	PRKCB; MAPK8; IL6; MAPK10; PYGL; IKBKB; SREBF1; INS; INSR; AKT2; PIK3CD; GSK3B; PTPN1; PPARA; NR1H3; NOS3; CPT1A; PIK3CA; TNF; AKT1	20	0.035 492
3	AGE-RAGE signaling pathway in diabetic complicatio ns	Human Diseases	Endocrine and metabolic diseases	PRKCA; IL6; VEGFA; AGTR1; MAPK3; MAPK8; MAPK14; PIK3CA; THBD; SERPINE1; AKT2; PIK3CD; NOS3; TNF; PRKCB; IL1B; MMP2; MAPK10; PLCD1; PIM1; BCL2; AKT1	22	0.035 492
**Infectious diseases**						
1	Pathogenic Escherichia coli infection	Human Diseases	Infectious diseases: Bacterial	ACTB; TUBA1C; TUBB2B; PRKCA; TUBA1A; TUBB2A; TUBA1B; TUBA8; TUBA3E; TUBB4B; TUBB4A; TUBB6; TLR4; TUBB1; TUBB3; TUBB; TUBA4A	17	0.001 242
2	Human papillomavi rus infection	Human Diseases	Infectious diseases: Viral	VEGFA; IKBKB; PRKACA; HDAC9; HDAC8; CDK2; MAP2K1; PPP2R1A; CDK6; MAPK3; HDAC1; EGFR; PIK3CA; HDAC7; PTK2; PDGFRB; ITGB3; GSK3B; COMP; HDAC11; HDAC2; ATP6V1B2; HDAC6; ATM; AKT2; PIK3CD; HDAC5; HDAC3; TNF; EGF; HDAC4; PTGS2; HDAC10; RAF1; AKT1	35	0.010 036
3	Influenza A	Human Diseases	Infectious diseases: Viral	IL12B; PRKCA; IL6; MAPK8; IKBKB; PRSS1; MAP2K1; CYCS; MAPK3; MAPK14; FDPS; GSK3B; CXCL10; PRKCB; AKT2; PIK3CD; TLR4; PLG; TNF; PIK3CA; ACTB; IL1B; MAPK10; RAF1; AKT1	25	0.039 86
**Neural diseases**						
1	Alcoholism	Human Diseases	Substance dependence	PRKACA; HDAC9; HDAC8; MAP2K1; MAPK3; DRD1; SLC18A2; HDAC1; SLC18A1; HDAC7; DRD2; HDAC11; HDAC2; ADORA2B; SLC6A3; HDAC6; CAMK4; HDAC5; HDAC3; HDAC4; MAOB; GRIN2C; HDAC10; PKIA; RAF1; ADORA2A	26	0.000 127
2	Morphine addiction	Human Diseases	Substance dependence	PDE4B; PRKCA; OPRM1; PRKACA; PDE4A; GABRA3; DRD1; KCNJ9; PDE1A; GABRA1; PDE4D; CACNA1B; CACNA1A; PDE7B; PDE8B; GABRB1; ADORA1; PRKCB; PDE4C; PDE1B; PDE3A; KCNJ6	22	0.012 364
3	EGFR tyrosine kinase inhibitor resistance	Human Diseases	Drug resistance: Antineoplast ic	PRKCB; PIK3CD; EGFR; VEGFA; EGF; RAF1; PRKCA; PIK3CA; AKT2; PDGFRB; GSK3B; SRC; BCL2; BCL2L1; MAP2K1; IGF1R; MAPK3; IL6; AKT1	19	0.018 902
4	Endocrine resistance	Human Diseases	Drug resistance: Antineoplast ic	MAPK14; PIK3CD; EGFR; PIK3CA; MMP2; MAPK10; GPER1; AKT2; ESR1; MAPK8; RAF1; SRC; BCL2; PRKACA; MAP2K1; IGF1R; ESR2; PTK2; MAPK3; AKT1	20	0.019 439

5	Amphetamine addiction	Human Diseases	Substance dependence	PRKCB; SLC18A1; MAOB; GRIA2; GRIA1; GRIN2C; PRKCA; PRKACA; CACNA1C; CAMK4; SLC6A3; CACNA1D; GRIA3; GRIA4; DRD1; SLC18A2; HDAC1	17	0.020024
**Organismal systems**						
**Circulatory system**						
1	Adrenergic signaling in cardiomyocytes	Organismal Systems	Circulatory system	PRKCA; SCN5A; PRKACA; AGTR1; MAPK3; MAPK14; CACNB2; ADRA1A; CACNA1S; CACNG1; CACNA1D; CACNA1F; TNNC1; BCL2; SCN1B; CACNB3; CACNB1; ADRB2; AKT2; ADRA1B; CACNB4; ADRB1; CACNA1C; CACNA2D3; CACNA2D2; PPP2R1A; CACNA2D1; SLC8A1; AKT1; ADRA1D	30	0.000179
2	Vascular smooth muscle contraction	Organismal Systems	Circulatory system	PRKCA; PTGIR; AGTR1; MAP2K1; MAPK3; KCNMA1; PLA2G1B; PLA2G2D; ADRA1A; CACNA1S; CACNA1D; CACNA1F; PRKACA; ADORA2B; KCNMB2; ADRA1B; PLA2G2A; PRKCB; PLA2G2E; CACNA1C; ITPR3; RAF1; ADORA2A; GUCY1A2; ADRA1D	25	0.005001
**Endocrine system**						
1	Thyroid hormone signaling pathway	Organismal Systems	Endocrine system	PRKCA; THRB; PRKACA; PRKCB; MAP2K1; MAPK3; RXRG; HDAC1; PIK3CA; ITGB3; GSK3B; HDAC2; BMP4; RXRA; AKT2; ESR1; HDAC3; PIK3CD; NCOA2; ACTB; HIF1A; RXRB; PLCD1; RAF1; SRC; NCOA1; AKT1	27	0.000257
2	Estrogen signaling pathway	Organismal Systems	Endocrine system	OPRM1; PRKACA; PGR; MAP2K1; MAPK3; GPER1; EGFR; PIK3CA; KRT12; KCNJ9; HSP90AB1; AKT2; ESR1; HSP90AA1; NOS3; PIK3CD; ESR2; NCOA2; MMP2; ITPR3; RAF1; SRC; BCL2; KCNJ6; NCOA1; RARA; AKT1	27	0.000475
3	Renin secretion	Organismal Systems	Endocrine system	ADRB1; KCNMA1; REN; ACE; PDE3A; PRKACA; AGTR1; ITPR3; ADRB2; PDE1B; CACNA1S; CACNA1D; CACNA1F; PDE1A; ADRB3; GUCY1A2; CACNA1C; ADORA1	18	0.001665
4	Regulation of lipolysis in adipocytes	Organismal Systems	Endocrine system	ADRB1; PTGS2; PIK3CA; INS; AKT2; ADRB2; PTGS1; PRKACA; PTGER3; INSR; ADRB3; PIK3CD; ADORA1; AKT1	14	0.009018
5	Ovarian steroidogenesis	Organismal Systems	Endocrine system	PRKACA; PTGS2; CYP1B1; CYP1A1; LHCGR; CYP19A1; HSD17B2; ALOX5; AKR1C3; IGF1R; INSR; INS; HSD17B1	13	0.009692
6	Progesterone-mediated oocyte maturation	Organismal Systems	Endocrine system	MAPK14; MAPK8; MAPK10; PIK3CA; PRKACA; INS; PGR; AKT2; PIK3CD; CDK2; HSP90AB1; RAF1; IGF1R; MAP2K1; HSP90AA1; MAPK3; AKT1	17	0.023653
7	Oxytocin signaling pathway	Organismal Systems	Endocrine system	PRKCA; PRKACA; MAP2K1; MAPK3; EGFR; CACNB2; RYR1; KCNJ9; CAMK4; CACNA1S; CACNG1; CACNA1D; CACNA1F; CACNA2D1; PRKCB; CACNB3; CACNB1; NOS3; CACNB4; ACTB; PTGS2; CACNA1C; ITPR3; CACNA2D3; CACNA2D2; RAF1; SRC; KCNJ6; GUCY1A2	29	0.025354
8	Prolactin signaling pathway	Organismal Systems	Endocrine system	MAPK14; PIK3CD; GSK3B; PIK3CA; LHCGR; MAPK10; AKT2; ESR1; RAF1; SRC; MAP2K1; MAPK8; ESR2; INS; MAPK3; AKT1	16	0.026674
9	Parathyroid hormone synthesis, secretion and action	Organismal Systems	Endocrine system	PDE4B; PRKCB; CASR; RXRB; RXRA; PRKCA; PDE4D; ITPR3; PDE4A; EGFR; RAF1; BCL2; PRKACA; MAP2K1; MAPK3; RXRG; MMP13; PDE4C	18	0.03545
10	Insulin secretion	Organismal Systems	Endocrine system	KCNMA1; PRKCB; KCNN3; KCNJ11; PRKCA; PRKACA; KCNN2; ITPR3; FFAR1; CHRM3; KCNN4; CACNA1S; CACNA1D; CACNA1F; KCNMB2; INS; CACNA1C	17	0.03986
11	GnRH signaling pathway	Organismal Systems	Endocrine system	MAPK14; PRKCB; EGFR; MAPK10; MAPK8; PRKCA; MMP2; CACNA1C; ITPR3; CACNA1S; RAF1; CACNA1D; CACNA1F; PRKACA; MAP2K1; SRC; PTK2B; MAPK3	18	0.048786
**Immune system**						
1	IL-17 signaling pathway	Organismal Systems	Immune system	MAPK14; MMP3; IL6; PTGS2; S100A8; IKBKB; MAPK10; CEBPB; HSP90AB1; GSK3B; MAPK3; CXCL10; MAPK8; HSP90AA1; IL1B; TNF; MMP13; S100A9	18	0.03545
**Nervous system**						
1	Serotonergic synapse	Organismal Systems	Nervous system	PRKCA; HTR7; HTR3B; MAP2K1; HTR2B; MAPK3; SLC18A2; HTR6; SLC18A1; HTR5A; KCNJ9; HTR1B; ALOX5; CACNA1S; HTR1D; CACNA1D; CACNA1F; PRKACA; HTR2A; SLC6A4; HTR1A; HTR2C; CACNA1B; CACNA1A; HTR1E;	39	2.12E-09

				HTR1F; GABRB1; PRKCB; HTR3A; MAOB; PTGS2; HTR4; CACNA1C; ITPR3; PTGS1; RAF1; APP; KCNJ6; KCNN2		
2	Dopaminergic synapse	Organismal Systems	Nervous system	PRKCA; DRD5; MAPK10; MAPK8; GRIA2; PRKACA; CALY; SCN1A; DRD1; SLC18A2; MAPK14; SLC18A1; KCNJ9; DRD2; PPP2R1A; CACNA1D; GRIA4; SLC6A3; GRIA1; CACNA1B; CACNA1A; DRD3; AKT2; GRIA3; DRD4; PRKCB; MAOB; CACNA1C; ITPR3; COMT; GSK3B; KCNJ6; AKT1	33	1.15E-06
3	Cholinergic synapse	Organismal Systems	Nervous system	KCNQ2; PRKCA; CHRNA6; CHRM4; PRKACA; MAP2K1; MAPK3; PIK3CA; CACNA1S; CACNA1D; CACNA1F; CHRNA4; CHRM2; CHRM1; CAMK4; CACNA1B; CACNA1A; AKT2; CHRM3; PIK3CD; CHRM5; PRKCB; CHRNA3; CACNA1C; ITPR3; ACHE; KCNQ3; BCL2; KCNJ6; AKT1	30	2.73E-05
4	Long-term depression	Organismal Systems	Nervous system	PRKCB; ITPR3; RYR1; GRIA1; PRKCA; CACNA1A; MAP2K1; RAF1; IGF1R; PPP2R1A; GRIA3; GRIA2; MAPK3; GUCY1A2	14	0.023653
**Digestive system**						
1	Salivary secretion	Organismal Systems	Digestive system	ADRB1; KCNMA1; PRKCB; PRKCA; PRKACA; ITPR3; ADRA1A; CHRM3; KCNN4; ADRB2; ADRA1B; ADRB3; GUCY1A2; ADRA1D	14	0.023833
**Others**						
1	Circadian entrainment	Organismal Systems	Environmental adaptation	MTNR1B; PRKCB; MAPK3; RYR1; GRIA1; KCNJ9; PRKCA; PRKACA; CACNA1C; ITPR3; MTNR1A; GRIN2C; CACNA1D; CACNA1G; KCNJ6; GRIA3; GRIA2; GRIA4; GUCY1A2; CACNA1I; CACNA1H	21	0.000645
2	Taste transduction	Organismal Systems	Sensory system	PRKACA; HTR1A; HTR3A; GABRA1; ITPR3; HTR1B; HTR1D; CACNA1C; CACNA1A; HTR3B; HTR1E; CHRM3; HTR1F; PDE1B; GABRA3; PDE1A; SCN2A	17	0.004532
**Cellular processes**						
1	Gap junction	Cellular Processes	Cellular community - eukaryotes	PRKCA; TUBA1C; PRKACA; TUBB4A; MAP2K1; TUBB1; HTR2B; MAPK3; DRD1; TUBB3; EGFR; TUBB6; TUBA8; DRD2; PDGFRB; TUBB4B; HTR2C; HTR2A; TUBB; TUBB2B; EGF; TUBA1B; TUBA3E; ADRB1; PRKCB; TUBA1A; ITPR3; RAF1; SRC; TUBB2A; GUCY1A2; TUBA4A	32	3.12E-08
2	Apoptosis	Cellular Processes	Cell growth and death	ITPR3; TUBA1C; MAPK8; IKBKB; NGF; MAP2K1; PARP3; CYCS; MAPK3; PIK3CA; CTSK; TUBA8; CSF2RB; BIRC5; PARP2; ATM; TUBA1B; AKT2; PARP1; TUBA3E; PIK3CD; TNF; ACTB; TUBA1A; MAPK10; RAF1; BCL2; BCL2L1; TUBA4A; AKT1	30	0.000501
3	Phagosome	Cellular Processes	Transport and catabolism	TUBB4A; CANX; TUBB1; TUBB3; ITGB3; TUBB6; TUBA8; TUBB4B; COMP; TUBB2B; CALR; TUBB; ATP6V1B2; TUBA1B; TUBA3E; TLR4; MPO; ACTB; TUBA1A; TUBA1C; TUBB2A; TUBA4A	22	0.019248
**Metabolism**						
1	Steroid hormone biosynthesis	Metabolism	Lipid metabolism	SRD5A1; AKR1C1; UGT1A9; SULT2B1; CYP1B1; CYP1A1; CYP11B2; CYP19A1; HSD17B2; COMT; AKR1C3; HSD11B1; HSD17B1	13	0.048845
**Genetic information processing**						
1	Proteasome	Genetic Information Processing	Folding, sorting and degradation	PSMB6; PSMA2; PSMA4; PSMB7; PSMB4; PSMB8; PSMB5; PSMA1; PSMA6; PSMA3; PSMB10; PSMB9; PSMA7; PSMB1; PSMB2; PSMB3	16	3.35E-07

Supplementary References

1. Fernandez-Ramos, A. A., et al. (2017). "6-mercaptopurine promotes energetic failure in proliferating T cells." Oncotarget 8(26): 43048-43060.
2. Mohammadpour, R., et al. (2014). "Acetazolamide triggers death inducing autophagy in T-47D breast cancer cells." Cell Biology International 38(2): 228-238.
3. Zhao, Y. G. and H. Zhang (2016). "ULK1 cycling: The ups and downs of the autophagy response." Journal of Cell Biology 215(6): 757-759.
4. Hu, X., et al. (2017). "Blocking autophagy improves the anti-tumor activity of afatinib in lung adenocarcinoma with activating EGFR mutations in vitro and in vivo." Scientific Reports 7(1): 4559.
5. Wang, F., et al. (2015). "Alisertib induces cell cycle arrest and autophagy and suppresses epithelial-to-mesenchymal transition involving PI3K/Akt/mTOR and sirtuin 1-mediated signaling pathways in human pancreatic cancer cells." Drug Design, Development and Therapy 9: 575-601.
6. Lee, K. Y., et al. (2013). "Activation of autophagy rescues amiodarone-induced apoptosis of lung epithelial cells and pulmonary toxicity in rats." Toxicological Sciences 136(1): 193-204.
7. Castoldi, F., et al. (2018). "Aspirin induces autophagy via inhibition of the acetyltransferase EP300." Oncotarget 9(37): 24574-24575.
8. Petersen, W., et al. (2014). "Dasatinib suppression of medulloblastoma survival and migration is markedly enhanced by combining treatment with the aurora kinase inhibitor AT9283." Cancer Letters 354(1): 68-76.
9. Huuskonen, M. T., et al. (2016). "Bexarotene targets autophagy and is protective against thromboembolic stroke in aged mice with tauopathy." Scientific Reports 6: 33176.
10. Boutin, B., et al. (2013). "Androgen deprivation and androgen receptor competition by bicalutamide induce autophagy of hormone-resistant prostate cancer cells and confer resistance to apoptosis." Prostate 73(10): 1090-1102.
11. Huo, R., et al. (2016). "Cabazitaxel-induced autophagy via the PI3K/Akt/mTOR pathway contributes to A549 cell death." Molecular Medicine Reports 14(4): 3013-3020.
12. Lin, S. J., et al. (2017). "Pituitary Tumor Suppression by Combination of Cabergoline and Chloroquine." The Journal of Clinical Endocrinology and Metabolism 102(10): 3692-3703.
13. Lin, Y. T., et al. (2017). "Capsaicin Induces Autophagy and Apoptosis in Human Nasopharyngeal Carcinoma Cells by Downregulating the PI3K/AKT/mTOR Pathway." International Journal of Molecular Sciences 18(7).
14. Puls, F., et al. (2013). "Autophagy-enhancing drug carbamazepine diminishes hepatocellular death in fibrinogen storage disease." Journal of Hepatology 59(3): 626-630.
15. Zang, Y., et al. (2012). "The next generation proteasome inhibitors carfilzomib and oprozomib activate prosurvival autophagy via induction of the unfolded protein response and ATF4." Autophagy 8(12): 1873-1874.
16. Zhou, H., et al. (2014). "Ciclopirox induces autophagy through reactive oxygen species-mediated activation of JNK signaling pathway." Oncotarget 5(20): 10140-10150.
17. Lee, H. R., et al. (2015). "Cilostazol Upregulates Autophagy via SIRT1 Activation: Reducing Amyloid-beta Peptide and APP-CTFbeta Levels in Neuronal Cells." PLoS One 10(8): e0134486.
18. Li, C. L., et al. (2013). "Mechanism for clofarabine inducing autophagic death of acute myelocytic leukemia cell U937." Zhongguo Shi Yan Xue Ye Xue Za Zhi 21(2): 347-350.
19. Bhattacharya, S., et al. (2016). "Colchicine induces autophagy and senescence in lung cancer cells at clinically admissible concentration: potential use of colchicine in combination with autophagy inhibitor in cancer therapy." Tumour Biology 37(8): 10653-10664.
20. Sun, C. Y., et al. (2017). "Norcantharidin alone or in combination with crizotinib induces autophagic cell death in hepatocellular carcinoma by repressing c-Met-mTOR signaling." Oncotarget 8(70): 114945-114955.

21. Ke, D., et al. (2019). "Curcumin-activated autophagy plays a negative role in its anti-osteoclastogenic effect." Molecular and Cellular Endocrinology: 110637.
22. Chen, L. Y., et al. (2017). "Autophagy is an important event for low-dose cytarabine treatment in acute myeloid leukemia cells." Leukemia Research 60: 44-52.
23. Milano, V., et al. (2009). "Dasatinib-induced autophagy is enhanced in combination with temozolomide in glioma." Molecular Cancer Therapeutics 8(2): 394-406.
24. Zhou, R., et al. (2019). "Low-dose Dexamethasone Increases Autophagy in Cerebral Cortical Neurons of Juvenile Rats with Sepsis Associated Encephalopathy." Neuroscience 419: 83-99.
25. Meng, Z., et al. (2018). "Diazoxide ameliorates severity of experimental osteoarthritis by activating autophagy via modulation of the osteoarthritis-related biomarkers." Journal of Cellular Biochemistry 119(11): 8922-8936.
26. Choi, J., et al. (2015). "Dienogest enhances autophagy induction in endometriotic cells by impairing activation of AKT, ERK1/2, and mTOR." Fertility and Sterility 104(3): 655-664 e651.
27. Culig, Z. (2017). "Molecular Mechanisms of Enzalutamide Resistance in Prostate Cancer." Current Molecular Biology Reports 3(4): 230-235.
28. Jiang, X., et al. (2018). "Repurposing sertraline sensitizes non-small cell lung cancer cells to erlotinib by inducing autophagy." JCI Insight 3(11): 98921.
29. Polager, S., et al. (2008). "E2F1 regulates autophagy and the transcription of autophagy genes." Oncogene 27(35): 4860-4864.
30. Kim, S. H., et al. (2017). "Ezetimibe ameliorates steatohepatitis via AMP activated protein kinase-TFEB-mediated activation of autophagy and NLRP3 inflammasome inhibition." Autophagy 13(10): 1767-1781.
31. Gao, H., et al. (2016). "Rho-Kinase inhibitor fasudil suppresses high glucose-induced H9c2 cell apoptosis through activation of autophagy." Cardiovascular Therapeutics 34(5): 352-359.
32. Zhang, J., et al. (2016). "Fenofibrate increases cardiac autophagy via FGF21/SIRT1 and prevents fibrosis and inflammation in the hearts of Type 1 diabetic mice." Clinal Science (London) 130(8): 625-641.
33. Mahoney, E., et al. (2012). "ER stress and autophagy: new discoveries in the mechanism of action and drug resistance of the cyclin-dependent kinase inhibitor flavopiridol." Blood 120(6): 1262-1273.
34. Po, W. W., et al. (2019). "Fluoxetine Simultaneously Induces Both Apoptosis and Autophagy in Human Gastric Adenocarcinoma Cells." Biomolecules & Therapeutics (Seoul) 28(2): 202-210.
35. Cook, K. L., et al. (2014). "Mitochondria directly donate their membrane to form autophagosomes during a novel mechanism of parkin-associated mitophagy." Cell and Bioscience 4: 16.
36. Zhao, Z. Q., et al. (2016). "Gefitinib induces lung cancer cell autophagy and apoptosis via blockade of the PI3K/AKT/mTOR pathway." Oncology Letters 12(1): 63-68.
37. Pardo, R., et al. (2010). "Gemcitabine induces the VMP1-mediated autophagy pathway to promote apoptotic death in human pancreatic cancer cells." Pancreatology 10(1): 19-26.
38. Wang, Y., et al. (2018). "Genistein and Myd88 Activate Autophagy in High Glucose-Induced Renal Podocytes In Vitro." Medical Science Monitor 24: 4823-4831.
39. Zhou, J., et al. (2019). "Glibenclamide-Induced Autophagy Inhibits Its Insulin Secretion-Improving Function in beta Cells." International Journal of Endocrinology 2019: 1265175.
40. Palomer, X., et al. (2014). "PPARbeta/delta attenuates palmitate-induced endoplasmic reticulum stress and induces autophagic markers in human cardiac cells." International Journal of Cardiology 174(1): 110-118.
41. Saiprasad, G., et al. (2014). "Hesperidin induces apoptosis and triggers autophagic markers through inhibition of Aurora-A mediated phosphoinositide-3-kinase/Akt/mammalian target of rapamycin and glycogen synthase kinase-3 beta signalling cascades in experimental colon carcinogenesis." European Journal of Cancer 50(14): 2489-2507.

42. Molina, V., et al. (2020). "Patterns of Apoptosis and Autophagy Activation After Hydroxyurea Exposure in the Rat Cerebellar External Granular Layer: an Immunoperoxidase and Ultrastructural Analysis." Neurotoxicity Research 37(1): 93-99.
43. Xie, Q., et al. (2017). "Imatinib induces autophagy via upregulating XIAP in GIST882 cells." Biochemical and Biophysical Research Communications 488(4): 584-589.
44. Chang, S. H., et al. (2017). "Imiquimod-induced autophagy is regulated by ER stress-mediated PKR activation in cancer cells." Journal of Dermatological Science 87(2): 138-148.
45. Kim, J. J., et al. (2012). "Host Cell Autophagy Activated by Antibiotics Is Required for Their Effective Antimycobacterial Drug Action." Cell Host & Microbe 11(5): 457-468.
46. Liu, R., et al. (2014). "Itraconazole suppresses the growth of glioblastoma through induction of autophagy Involvement of abnormal cholesterol trafficking." Autophagy 10(7): 1241-1255.
47. Wang, K., et al. (2016). "Ivermectin induces PAK1-mediated cytostatic autophagy in breast cancer." Autophagy 12(12): 2498-2499.
48. Talaei, F., & Atyabi, F. (2013). "Anti-Aging Effects of Ketanserin; Ketanserin Extends Lifespan in Female Drosophila, Inhibits Cellular Senescence and Promotes Wound Healing In-vitro. " Annual Research & Review in Biology, 3(4): 888-902.
49. Wu, H., et al. (2015). "Lamotrigine Reduces beta-Site AbetaPP-Cleaving Enzyme 1 Protein Levels Through Induction of Autophagy." Journal of Alzheimer’s Disease 46(4): 863-876.
50. Huang, H. L., et al. (2011). "Lapatinib induces autophagy, apoptosis and megakaryocytic differentiation in chronic myelogenous leukemia K562 cells." PLoS One 6(12): e29014.
51. Yu, C., et al. (2018). "Autophagy: novel applications of nonsteroidal anti-inflammatory drugs for primary cancer." Cancer Medicine 7(2): 471-484.
52. Holczer, M., et al. (2015). "A Comprehensive Systems Biological Study of Autophagy-Apoptosis Crosstalk during Endoplasmic Reticulum Stress." Biomed Research International 2015: 319589.
53. Gara, R. K., et al. (2014). "Induction of autophagy by ormeloxifene and mevastatin through Protein Kinase D1 in prostate cancer cells." Cancer Research 74(19): 1334.
54. Zhang, L., et al. (2016). "Mifepristone increases mRNA translation rate, triggers the unfolded protein response, increases autophagic flux, and kills ovarian cancer cells in combination with proteasome or lysosome inhibitors." Molecular Oncology 10(7): 1099-1117.
55. Boumber, Y., et al. (2011). "Mocetinostat (MGCD0103): a review of an isotype-specific histone deacetylase inhibitor." Expert Opinion on Investigational Drugs 20(6): 823-829.
56. Kim, S. W., et al. (2016). "Niacin alleviates TRAIL-mediated colon cancer cell death via autophagy flux activation." Oncotarget 7(4): 4356-4368.
57. Bao, X. X., et al. (2012). "Nifedipine induced autophagy through Beclin1 and mTOR pathway in endometrial carcinoma cells." Chinese Medical Journal (English) 125(17): 3120-3126.
58. Yu, H. C., et al. (2013). "Nilotinib induces autophagy in hepatocellular carcinoma through AMPK activation." Journal of Biological Chemistry 288(25): 18249-18259.
59. Chang, W. L., et al. (2015). "Repurposing of nitroxoline as a potential anticancer agent against human prostate cancer: a crucial role on AMPK/mTOR signaling pathway and the interplay with Chk2 activation." Oncotarget 6(37): 39806-39820.
60. Guzman-Beltran, S., et al. (2016). "Nordihydroguaiaretic acid (NDGA) and alpha-mangostin inhibit the growth of Mycobacterium tuberculosis by inducing autophagy." International Immunopharmacology 31: 149-157.
61. Altinoz, M. A., et al. (2006). "Noscapine and diltiazem augment taxol and radiation-induced S-phase arrest and clonogenic death of C6 glioma in vitro." Surgical Neurology 65(5): 478-484; discussion 485.

62. Cournoyer, S., et al. (2019). "GX15-070 (Obatoclax), a Bcl-2 family proteins inhibitor engenders apoptosis and pro-survival autophagy and increases Chemosensitivity in neuroblastoma." BMC Cancer 19(1): 1018.
63. Zhu, Y., et al. (2019). "Olanzapine induced autophagy through suppression of NF-kappaB activation in human glioma cells." CNS Neuroscience & Therapeutics 25(9): 911-921.
64. Arun, B., et al. (2015). "The PARP inhibitor AZD2281 (Olaparib) induces autophagy/mitophagy in BRCA1 and BRCA2 mutant breast cancer cells." International Journal of Oncology 47(1): 262-268.
65. Gandesiri, M., et al. (2016). "Erratum to: DAPK plays an important role in panobinostat-induced autophagy and commits cells to apoptosis under autophagy deficient conditions." Apoptosis 21(5): 671-674.
66. Tavallai, S., et al. (2014). "Pazopanib and HDAC inhibitors interact to kill sarcoma cells." Cancer Biology & Therapy 15(5): 578-585.
67. Park, J. H., et al. (2014). "The role of autophagy induced by pemetrexed in lung adenocarcinoma cells." Oncology Reports 31(5): 2365-2370.
68. Sharma, K., et al. (2016). "Pentoxifylline triggers autophagy via ER stress response that interferes with Pentoxifylline induced apoptosis in human melanoma cells." Biochemical Pharmacology 103: 17-28.
69. Tong, Y., et al. (2012). "Perifosine induces protective autophagy and upregulation of ATG5 in human chronic myelogenous leukemia cells in vitro." Acta Pharmacologica Sinica 33(4): 542-550.
70. Siedlecka-Kroplewska, K., et al. (2019). "Induction of autophagy, apoptosis and aquisition of resistance in response to piceatannol toxicity in MOLT-4 human leukemia cells." Toxicology In Vitro 59: 12-25.
71. Liu, J., et al. (2016). "Piperine induces autophagy by enhancing protein phosphotase 2A activity in a rotenone-induced Parkinson's disease model." Oncotarget 7(38): 60823-60843.
72. Gorski, S. M., et al. (2012). "Targeting autophagy: the Achilles' heel of cancer." Autophagy 8(8): 1279-1280.
73. Kim, H. N., et al. (2012). "The neurosteroids, allopregnanolone and progesterone, induce autophagy in cultured astrocytes." Neurochemistry International 60(2): 125-133.
74. Coker-Gurkan, A., et al. (2015). "Purvalanol induces endoplasmic reticulum stress-mediated apoptosis and autophagy in a time-dependent manner in HCT116 colon cancer cells." Oncology Reports 33(6): 2761-2770.
75. Cao, H., et al. (2019). "Quercetin has a protective effect on atherosclerosis via enhancement of autophagy in ApoE(-/-) mice." Experimental and Therapeutic Medicine 18(4): 2451-2458.
76. Ouchida, A. T., et al. (2018). "Synergistic effect of a novel autophagy inhibitor and Quizartinib enhances cancer cell death." Cell Death & Disease 9(2): 138.
77. Weng, Z., et al. (2015). "Regorafenib impairs mitochondrial functions, activates AMP-activated protein kinase, induces autophagy, and causes rat hepatocyte necrosis." Toxicology 327: 10-21.
78. Wang, N., et al. (2019). "Resveratrol Activates Autophagy via the AKT/mTOR Signaling Pathway to Improve Cognitive Dysfunction in Rats With Chronic Cerebral Hypoperfusion." Frontiers in Neuroscience 13: 859.
79. Bagca, B. G., et al. (2016). "Ruxolitinib induces autophagy in chronic myeloid leukemia cells." Tumor Biology 37(2): 1573-1579.
80. Mutlu, Z., et al. (2016). "Upregulation of transmembrane transcription factor, ATF6 and cAMP response element-binding protein 3, CREB3 gene expression levels by ruxolitinib leads to ER stress-induced autophagy in CML." The FEBS Journal 283: 100-101.
81. Wang, X. D., et al. (2016). "Salicylic acid promotes autophagy via NPR3 and NPR4 in Arabidopsis senescence and innate immune response." Acta Physiologiae Plantarum 38(10): 1-12.
82. Shin, J. H., et al. (2012). "Sertindole, a Potent Antagonist at Dopamine D-2 Receptors, Induces Autophagy by Increasing Reactive Oxygen Species in SH-SY5Y Neuroblastoma Cells." Biological & Pharmaceutical Bulletin 35(7): 1069-1075.
83. Atef, M. M., et al. (2019). "Ameliorative effects of autophagy inducer, simvastatin on alcohol-induced liver disease in a rat model." Journal of Cellular Biochemistry 120(5): 7679-7688.

84. Prieto-Dominguez, N., et al. (2016). "Modulation of Autophagy by Sorafenib: Effects on Treatment Response." Frontiers in Pharmacology 7: 151.
85. Li, D., et al. (2016). "Spironolactone promotes autophagy via inhibiting PI3K/AKT/mTOR signalling pathway and reduce adhesive capacity damage in podocytes under mechanical stress." Bioscience Reports 36(4): e00355.
86. Han, H. Y., et al. (2014). "Sulfasalazine induces autophagic cell death in oral cancer cells via Akt and ERK pathways." Asian Pac J Cancer Prev 15(16): 6939-6944.
87. Gurpinar, E., et al. (2013). "A Novel Sulindac Derivative Inhibits Lung Adenocarcinoma Cell Growth through Suppression of Akt/mTOR Signaling and Induction of Autophagy." Molecular Cancer Therapeutics 12(5): 663-674.
88. Fields, J. A., et al. (2017). "The anticancer drug sunitinib promotes autophagy and protects from neurotoxicity in an HIV-1 Tat model of neurodegeneration." Journal of Neurovirology 23(2): 290-303.
89. Xu, X. S., et al. (2018). "Tacrolimus alleviates Ox-LDL damage through inducing vascular endothelial autophagy." European Review for Medical and Pharmacological Sciences 22(10): 3199-3206.
90. Torres-Lopez, L., et al. (2019). "Tamoxifen induces toxicity, causes autophagy, and partially reverses dexamethasone resistance in Jurkat T cells." Journal of Leukocyte Biology 105(5): 983-998.
91. Yang, L., et al. (2019). "Taurine protects against arsenic trioxide-induced insulin resistance via ROS-Autophagy pathway in skeletal muscle." International Journal of Biochemistry & Cell Biology 112: 50-60.
92. Kozako, T., et al. (2016). "Angiotensin II type 1 receptor blocker telmisartan induces apoptosis and autophagy in adult T-cell leukemia cells." FEBS Open Bio 6(5): 442-460.
93. Wu, Y., et al. (2015). "Mechanisms of tolvaptan-induced toxicity in HepG2 cells." Biochem Pharmacol 95(4): 324-336.
94. Li, D. D., et al. (2009). "The pivotal role of c-Jun NH2-terminal kinase-mediated Beclin 1 expression during anticancer agents-induced autophagy in cancer cells." Oncogene 28(6): 886-898.
95. Nazim, U. M., et al. (2017). "PPARgamma activation by troglitazone enhances human lung cancer cells to TRAIL-induced apoptosis via autophagy flux." Oncotarget 8(16): 26819-26831.
96. Xia, Q., et al. (2016). "Valproic acid induces autophagy by suppressing the Akt/mTOR pathway in human prostate cancer cells." Oncology Letters 12(3): 1826-1832.
97. Ma, X. H., et al. (2014). "Targeting ER stress-induced autophagy overcomes BRAF inhibitor resistance in melanoma." Journal of Clinal Investigation 124(3): 1406-1417.
98. Sun, W. L., et al. (2015). "Autophagy facilitates multidrug resistance development through inhibition of apoptosis in breast cancer cells." Neoplasma 62(2): 199-208.
99. Zeng, X. and D. W. Ju (2018). "Hedgehog Signaling Pathway and Autophagy in Cancer." International Journal of Molecular Sciences 19(8): e2279.
100. Zhang, F. and C. M. Ma (2019). "Kaempferol suppresses human gastric cancer SNU-216 cell proliferation, promotes cell autophagy, but has no influence on cell apoptosis." Brazilian Journal of Medical and Biological Research 52(2): e7843.
101. Zhao, X. L., et al. (2018). "Nicotine induced autophagy of Leydig cells rather than apoptosis is the major reason of the decrease of serum testosterone." International Journal of Biochemistry & Cell Biology 100: 30-41.
102. Kohli, L., et al. (2013). "4-Hydroxytamoxifen Induces Autophagic Death through K-Ras Degradation." Cancer Research 73(14): 4395-4405.
103. Zhao, M., et al. (2013). "Acetylcholine mediates AMPK-dependent autophagic cytoprotection in H9c2 cells during hypoxia/reoxygenation injury." Cellular Physiology and Biochemistry 32(3): 601-613.
104. Hu, M. B., et al. (2018). "Atorvastatin induces autophagy in MDA-MB-231 breast cancer cells." Ultrastructural Pathology 42(5): 409-415.
105. Shi, X., et al. (2019). "Metronomic photodynamic therapy with 5-aminolevulinic acid induces apoptosis and autophagy in human SW837 colorectal cancer cells." Journal of Photochemistry and Photobiology B 198: 111586.

106. Wang, X. Y., et al. (2017). "Bardoxolone methyl (CDDO-Me or RTA402) induces cell cycle arrest, apoptosis and autophagy via PI3K/Akt/mTOR and p38 MAPK/Erk1/2 signaling pathways in K562 cells." American Journal of Translational Research 9(10): 4652-4672.
107. Li, Y., et al. (2016). "A cell-based quantitative high-throughput image screening identified novel autophagy modulators." Pharmacological Research 110: 35-49.
108. Zschocke, J. and T. Rein (2011). "Antidepressants encounter autophagy in neural cells." Autophagy 7(10): 1247-1248.
109. Apolloni, S., et al. (2016). "Actions of the antihistaminergic clemastine on presymptomatic SOD1-G93A mice ameliorate ALS disease progression." Journal of Neuroinflammation 13(1): 191.
110. Wu, Y., et al. (2010). "Neuroprotection of deferoxamine on rotenone-induced injury via accumulation of HIF-1 alpha and induction of autophagy in SH-SY5Y cells." Neurochemistry International 57(3): 198-205.
111. Park R., et al. (2017). "Reserpine treatment activates AMP activated protein kinase (AMPK)." Natural Product Sciences. 2017 Sep;23(3):157-161.
112. Forbes, A., et al. (2016). "Relative cytotoxic potencies and cell death mechanisms of α1-adrenoceptor antagonists in prostate cancer cell lines." Prostate 76(8): 757-66.
113. Belur, N. A., et al. (2018). "Evaluating class III antiarrhythmic agents as novel MYC targeting drugs in ovarian cancer." Gynecologic Oncology 151(3): 525-532.
114. Yang, Z., et al. (2017). "Fluvastatin prevents lung adenocarcinoma bone metastasis by triggering autophagy." EBioMedicine 19: 49-59.
115. Ristic, B., et al. (2014). "Idarubicin induces mTOR-dependent cytotoxic autophagy in leukemic cells." Experimental Cell Research 326(1): 90-102.
116. Jose, C., et al. (2018). "Redox mechanism of levobupivacaine cytostatic effect on human prostate cancer cells." Redox Biology 18: 33-42.
117. Juarez, E., et al. (2016). "Loperamide restricts intracellular growth of mycobacterium tuberculosis in lung macrophages." American Journal of Respiratory Cell and Molecular Biology 55(6): 837-847.
118. Cloonan, S. M., et al. (2011). "The antidepressants maprotiline and fluoxetine induce type II autophagic cell death in drug-resistant Burkitt's lymphoma." International Journal of Cancer 128(7): 1712-23.
119. Hirano, K., et al. (2019). "Neuroprotective effects of memantine via enhancement of autophagy." Biochemical and Biophysical Research Communications 518(1): 161-170.
120. Chen, Z., et al. (2019). "Metformin treatment alleviates polycystic ovary syndrome by decreasing the expression of MMP-2 and MMP-9 via H19/miR-29b-3p and AKT/mTOR/autophagy signaling pathways." Journal of Cellular Physiology 234(11): 19964-19976.
121. Correia, I., et al. (2014). "Effects of naproxen on cell proliferation and genotoxicity in MG-63 osteosarcoma cell line." Journal of Toxicology and Environmental Health Part A 77(14-16): 916-23.
122. Ochi, M., et al. (2015). "Protective effect of N-acetylcysteine against nicardipine hydrochloride-induced autophagic cell death of human vascular endothelial cells." The Journal of Toxicological Sciences 40(5): 551-8.
123. Son, M. J., et al. (2010). "Sodium nitroprusside induces autophagic cell death in glutathione-depleted osteoblasts." Journal of Biochemical and Molecular Toxicology 24(5): 313-22.
124. Sundaramurthy, V., et al. (2013). "Integration of chemical and RNAi multiparametric profiles identifiers triggers of intracellular mycobacterial killing." Cell Host & Microbe 13(2): 129-42.
125. Han, W., et al. (2011). "Autophagy inhibition enhances daunorubicin-induced apoptosis in K562 cells." PLoS One 6(12): e28491.
126. Zhou, Y., et al. (2019). "miR-223 overexpression inhibits doxorubicin-induced autophagy by targeting FOXO3a and reverses chemoresistance in hepatocellular carcinoma cells." Cell Death & Disease 10(11): 843.

127. Carames, B., et al. (2013). "Glucosamine activates autophagy in vitro and in vivo." Arthritis & Rheumatology 65(7): 1843-52.
128. Song, H. L., et al. (2019). "Ouabain activates transcription factor EB and exerts neuroprotectin in models of Alzheimer's disease." Molecular and Cellular Neuroscience 95: 13-24.
129. Alcocer-Gomez, E., et al. (2017). "Antidepressants induce autophagy dependent-NLRP3-inflammasone inhibition in major depressive disorder." Pharmacological Research 121: 114-121.
130. Yang, Y. F., et al. (2011). "Prazosin induces p53-mediated autophagic cell death in H9C2 cells." Naunyn-Schmiedeberg's Archives of Pharmacology 384(2): 209-16.
131. Zhang, M. S., et al. (2015). "Proflavin suppresses the growth of human osteosarcoma MG63 cells through apoptosis and autophagy." Oncology Letters 10(1): 463-468.
132. Huang, C., et al. (2010). "Autophagy induced by ischemic preconditioning is essential for cardioprotection." Journal of Cardiovascular Translational Research 3(4): 365-73.
133. Kang, S. Y., et al. (2017). "Autophagic modulation by rosuvastatin prevents rotenone-induced neurotoxicity in an in vitro model of Parkinson's disease." Neuroscience Letters 642: 20-26.
134. Vucicevic, L., et al. (2018). "Mechanisms and therapeutic significance of autophagy modulation by antipsychotic drugs." Cell Stress 2(11): 282-291.
135. Yang, Y., et al. (2019). "Trimetazidine ameliorates sunitinib-induced cardiotoxicity in mice via the AMPK/mTOR/autophagy pathway." Pharmaceutical Biology 57(1): 625-631.
136. Albert, J. M., et al. (2007). "Inhibition of poly (ADP-ribose) polymerase enhances cell death and improves tumor growth delay in irradiated lung cancer models." Clinical Cancer Research 13(10): 3033-42.
137. Wang, I. T., et al. (2014). "Zoledronic acid induces apoptosis and autophagy in cervical cancer cells." Tumor Biology 35(12): 11913-20.
138. Morissette, G., et al. (2008). "Intense pseudotransport of a cationic drug mediated by vacuolar ATPase: procainamide-induced autophagic cell vacuolization." Toxicology and Applied Pharmacology 228(3): 364-77.
139. Zhou, F., et al. (2018). "Raloxifene, a promising estrogen replacement, limits TDP-25 cell death by enhancing autophagy and suppressing apoptosis." Brain Research Bulletin 140: 281-290.
140. Aranguiz-Urroz, P., et al. (2011). "Beta (2) - adrenergic receptor regulates cardiac fibroblast autophagy and collagen degradation." Biochimica et Biophysica Acta 1812(1): 23-31.
141. Park, S., et al. (2020). "Selective autophagy of cytosolic protein aggregates involves ribosome-free rough endoplasmic reticulum." Histochemistry and Cell Biology 153(2): 89-99.
142. Vervliet, T., et al. (2017). "Basal ryanodine receptor activity suppresses autophagic flux." Biochemical Pharmacology 132: 133-142.
143. Liu, H., et al. (2019). "Methylxanthine derivatives promote autophagy in gastric cancer cells targeting PTEN." Anticancer Drugs 30(4): 347-355.
144. Katsuyama, Y., et al. (2017). "Disruption of melanosome transport in melanocytes treated with theophylline causes their degradation by autophagy." Biochemical and Biophysical Research Communications 485(1): 126-130.
145. Sarkar, S., et al. (2009). "Rapamycin and mTOR-independent autophagy inducers ameliorate toxicity of polyglutamine-expanded huntingtin and related proteinopathies." Cell Death and Differentiation 16(1): 46-56.
146. Ye, F. and Z. Y. Zuo (2017). "Anesthetic effects on autophagy." Medical Gas Research 7(3): 204-211.
147. Oikarinen, A. (2009). "Hydroxychloroquine Induces Autophagic Cell Death of Human Dermal Fibroblasts: Implications for Treating Fibrotic Skin Diseases." Journal of Investigative Dermatology 129(10): 2333-2335.
148. Zheng, Z. Y., et al. (2018). "Induction of N-Ras degradation by flunarizine-mediated autophagy." Scientific Reports 8.
149. Gravina, G. L., et al. (2016). "Dual PI3K/mTOR inhibitor, XL765 (SAR245409), shows superior effects to sole PI3K [XL147 (SAR245408)] or mTOR [rapamycin] inhibition in prostate cancer cell models." Tumor Biology 37(1): 341-351.

150. Siddiqi, F. H., et al. (2019). "Felodipine induces autophagy in mouse brains with pharmacokinetics amenable to repurposing." Nature Communications 10(1): 1817.
151. Anekonda, T. S. and J. F. Quinn (2011). "Calcium channel blocking as a therapeutic strategy for Alzheimer's disease: the case for isradipine." Biochimica Biophysica Acta 1812(12): 1584-1590.
152. Mori, M., et al. (2015). "Hsp90 inhibitor induces autophagy and apoptosis in osteosarcoma cells." International Journal of Oncology 46(1): 47-54.
153. Gao, S., et al. (2009). "Mechanism of thalidomide to enhance cytotoxicity of temozolomide in U251-MG glioma cells in vitro." Chinese Medical Journal (English) 122(11): 1260-1266.
154. Yu, K. N., et al. (2013). "Zinc oxide nanoparticle induced autophagic cell death and mitochondrial damage via reactive oxygen species generation." Toxicology In Vitro 27(4): 1187-1195.
155. Trejo-Solis, C., et al. (2012). "Copper compound induces autophagy and apoptosis of glioma cells by reactive oxygen species and JNK activation." BMC Cancer 12: 156.
156. Meschini, S., et al. (2008). "The plant alkaloid voacamine induces apoptosis-independent autophagic cell death on both sensitive and multidrug resistant human osteosarcoma cells." Autophagy 4(8): 1020-1033.
157. Konstat-Korzenny, E., et al. (2018). "Artemisinin and Its Synthetic Derivatives as a Possible Therapy for Cancer." Medical Sciences (Basel) 6(1): 19.
158. Vakifahmetoglu-Norberg, H., et al. (2015). "Pharmacologic agents targeting autophagy." The Journal of Clinical Investigation 125(1): 5-13.
159. Galluzzi, L., et al. (2017). "Pharmacological modulation of autophagy: therapeutic potential and persisting obstacles." Nature Reviews Drug Discovery 16(7): 487-511.
160. Rubinsztein, D. C., et al. (2012). "Autophagy modulation as a potential therapeutic target for diverse diseases." Nature Reviews Drug Discovery 11(9): 709-30.
161. Niu, J. F., et al. (2019). "Insight into the role of autophagy in osteosarcoma and its therapeutic implication." Frontiers in Oncology 9: 1232.
162. Vucicevic, L., et al. (2018). "Mechanisms and therapeutic significance of autophagy modulation by antipsychotic drugs." Cell Stress 2(11): 282-291.
163. Liu, Y. P., et al. (2018). "Cantharidin suppresses cell growth and migration, and activates autophagy in human non-small cell lung cancer cells." Oncology Letters 15(5): 6527-6532.
164. Caimmi, P. P., et al. (2011). "Intracoronary levosimendan prevents myocardial ischemic damages and activates survival signaling through ATP-sensitive potassium channel and nitric oxide." European Journal of Cardio-Thoracic Surgery 39(4): e59-67.
165. Marino, M. L., et al. (2010). "Proton pump inhibition induces autophagy as a survival mechanism following oxidative stress in human melanoma cells." Cell Death & Disease 1: e87.
166. Park, H. W., et al. (2014). "Pharmacological correction of obesity-induced autophagy arrest using calcium channel blockers." Nature Communications 5: 4834.
167. Renna, M., et al. (2011). "Azithromycin blocks autophagy and may predispose cystic fibrosis patients to mycobacterial infection." Journal of Clinical Investigation 121(9): 3554-3563.
168. Zheng, Q., et al. (2017). "Heparin-binding Hemagglutinin of Mycobacterium tuberculosis Is an Inhibitor of Autophagy." Front Cell Infect Microbiol 7: 33; "Heparin interaction with a receptor on hyperglycemic dividing cells prevents intracellular hyaluronan synthesis and autophagy responses in models of type 1 diabetes." Matrix Biology 48: 36-41.
169. Daiber, A. and T. Munzel (2015). "Organic Nitrate Therapy, Nitrate Tolerance, and Nitrate-Induced Endothelial Dysfunction: Emphasis on Redox Biology and Oxidative Stress." Antioxidants & Redox Signaling 23(11): 899-942.
170. Li, Y. N., et al. (2015). "Inhibition of HIF-1alpha Affects Autophagy Mediated Glycosylation in Oral Squamous Cell Carcinoma Cells." Disease Markers 2015: 239479.

171. Sharma, N., et al. (2012). "Inhibition of autophagy and induction of breast cancer cell death by mefloquine, an antimalarial agent." Cancer Letters 326(2): 143-154.
172. Hansen, A. R., et al. (2019). "Pantoprazole Affecting Docetaxel Resistance Pathways via Autophagy (PANDORA): Phase II Trial of High Dose Pantoprazole (Autophagy Inhibitor) with Docetaxel in Metastatic Castration-Resistant Prostate Cancer (mCRPC)." Oncologist 24(9): 1188-1194.
173. Ou, C., et al. (2019). "Chloroquine promotes gefitinibinduced apoptosis by inhibiting protective autophagy in cutaneous squamous cell carcinoma." Molecular Medicine Reports 20(6): 4855-4866.
174. Muller, G. and G. Weindl (2016). "Propranolol induces Th17-related cytokines and inhibits late-stage autophagy in cutaneous dendritic cells." Naunyn-Schmiedebergs Archives of Pharmacology 389(1): S65-S65.
175. Lobo, M. R., et al. (2014). "Combined Efficacy of Cediranib and Quinacrine in Glioma Is Enhanced by Hypoxia and Causally Linked to Autophagic Vacuole Accumulation." PLoS One 9(12): e114110.
176. Lu, J., et al. (2015). "Combining Epinephrine and Esmolol Attenuates Excessive Autophagy and Mitophagy in Rat Cardiomyocytes After Cardiac Arrest." Journal of Cardiovascular Pharmacology 66(5): 449-456.
177. Carella, A. M., et al. (2012). "Inhibition of autophagy with clarithromycin: a new strategy to enhance sensitivity of CML stem cells to tyrosine kinase inhibitors." Leukemia Supplement 1(Supplement 2): S49-50.
178. Furtado, C. M., et al. (2015). "Phosphatidylinositol-3-kinase as a putative target for anticancer action of clotrimazole." The International Journal of Biochemistry & Cell Biology 62: 132-141.
179. Li, Y., et al. (2015). "Ebselen reduces autophagic activation and cell death in the ipsilateral thalamus following focal cerebral infarction." Neuroscience Letters 600: 206-212.
180. Moriya, S., et al. (2013). "Macrolide antibiotics block autophagy flux and sensitize to bortezomib via endoplasmic reticulum stress-mediated CHOP induction in myeloma cells." International Journal of Oncology 42(5): 1541-1550.
181. Ryabaya, O. O., et al. (2017). "Autophagy inhibitors chloroquine and LY294002 enhance temozolomide cytotoxicity on cutaneous melanoma cell lines in vitro." Anticancer Drugs 28(3): 307-315.
182. Chen, H. C., et al. (2012). "Autophagy is activated in injured neurons and inhibited by methylprednisolone after experimental spinal cord injury." Spine (Phila Pa 1976) 37(6): 470-475.
183. Li, Y., et al. (2019). "Apoptotic effects of rhein through the mitochondrial pathways, two death receptor pathways, and reducing autophagy in human liver L02 cells." Environmental Toxicology 34(12): 1292-1302.
184. Lv, X. X., et al (2017). "Cigarette smoke promotes COPD by activating platelet-activating factor receptor and inducing neutrophil autophagic death in mice." Oncotarget 8(43): 74720-74735.
185. Xiao, R., et al (2012). "Myocardial autophagy after severe burn in rats." PLoS One 7(6): e39488.
186. Kandemir, F. M., et al. (2015). "Rutin attenuates gentamicin-induced renal damage by reducing oxidative stress, inflammation, apoptosis, and autophagy in rats." Renal Failure 37(3): 518-525.
187. Zhang, P. D., et al. (2017). "Rutin increases the cytotoxicity of temozolomide in glioblastoma via autophagy inhibition." Journal of Neuro-Oncology 132(3): 393-400.
188. Samidurai, A., et al. (2015). "PDE5 Inhibition with Sildenafil Blocks Induction of Carboxylesteras3 and Reduces Cell Necrosis and Autophagy in Acute Alcohol-Induced Injury in Heart." The FASEB Journal 29: 1.
189. Mishra, P., et al. (2017). "Discovery of pan autophagy inhibitors through a high-throughput screen highlights macroautophagy as an evolutionarily conserved process across 3 eukaryotic kingdoms." Autophagy 13(9): 1556-1572.
190. Jin, L. Y., et al. (2018). "Estradiol Alleviates Intervertebral Disc Degeneration through Modulating the Antioxidant Enzymes and Inhibiting Autophagy in the Model of Menopause Rats." Oxidative Medicine and Cellular Longevity 2018: 7890291.
191. Vallecillo-Hernandez, J., et al. (2018). "Indomethacin disrupts autophagic flux by inducing lysosomal dysfunction in gastric cancer cells and increases their sensitivity to cytotoxic drugs." Scientific Reports 8: 3593.

192. Shinde, A., et al. (2019). "Spleen tyrosine kinase-mediated autophagy is required for epithelial-mesenchymal plasticity and metastasis in breast cancer." Cancer Research 79(8): 1831-1843.
193. Sulli, G., et al. (2018). "Pharmacological activation of REV-ERBs is lethal in cancer and oncogene induced senescence." Nature 553(7688): 351-355.
194. Zhang, W. and J. Q. Zhang (2017). "Dexmedetomidine preconditioning protects against lung injury induced by ischemia-reperfusion through inhibition of autophagy." Experimental and Therapeutic Medicine 14(2): 973-980.
195. Zhu, C., et al. (2019). "Dexmedetomidine Protects Against Oxygen-Glucose Deprivation-Induced Injury Through Inducing Astrocytes Autophagy via TSC2/mTOR Pathway." Neuromolecular Medicine, *in press*.
196. Zheng, X. Y., et al. (2019). "Emodin-induced autophagy against cell apoptosis through the PI3K/AKT/mTOR pathway in human hepatocytes." Drug Design, Development and Therapy 13: 3171-3180.
197. Yu, X., et al. (2018). "Emodin Attenuates Autophagy Response to Protect the Pancreas From Acute Pancreatitis Failure." Pancreas 47(7): 892-897.
198. Wang, X. M., et al. (2015). "Attenuating Hypoxia-Induced Apoptosis and Autophagy of Mesenchymal Stem Cells: the Potential of Sitagliptin in Stem Cell-Based Therapy." Cellular Physiology and Biochemistry 37(5): 1914-1926.
199. Dai, X., et al. (2018). "Sitagliptin-mediated preservation of endothelial progenitor cell function via augmenting autophagy enhances ischaemic angiogenesis in diabetes." Journal of Cellular and Molecular Medicine 22(1): 89-100.
200. Xin, X. Y., et al. (2011). "2-Methoxyestradiol Attenuates Autophagy Activation After Global Ischemia." Canadian Journal of Neurological Sciences 38(4): 631-638.
201. Yang, C. H., et al. (2013). "RNA-Dependent Protein Kinase Is Essential for 2-Methoxyestradiol-Induced Autophagy in Osteosarcoma Cells." PLoS One 8(3): e59406.
202. Qiao, L., et al. (2017). "Corrigendum: Ginsenoside Rb1 Enhances Atherosclerotic Plaque Stability by Improving Autophagy and Lipid Metabolism in Macrophage Foam Cells." Frontiers in Pharmacology 8: 964.
203. Yang, T., et al. (2018). "Ginsenoside Rb1 inhibits autophagy through regulation of Rho/ROCK and PI3K/mTOR pathways in a pressure-overload heart failure rat model." Journal of Pharmacy and Pharmacology 70(6): 830-838.
204. Duan, X. C. and F. Zhang (2019). "Rosiglitazone prevents fibrosis after glaucoma filtration surgery by promoting autophagy: in vitro and in vivo." Investigative Ophthalmology & Visual Science 60(9).
205. Li, H. P., et al. (2017). "PPAR-gamma agonist rosiglitazone reduces autophagy and promotes functional recovery in experimental traumaticspinal cord injury." Neuroscience Letters 650: 89-96.
206. Mohammadinejad, R., et al. (2019). "Berberine as a potential autophagy modulator." Journal of Cellular Physiology 1-13.
207. Mahli, A., et al. (2018). "ERK activation and autophagy impairment are central mediaters of irinotecan-induced steatohepatitis." Gut 67(4): 746-756.
208. Stanislav, J., et al. (2013). "The role of autophagic cell death and apoptosis in irinotecan-treated p53 null colon cancer cells." Anti-Cancer Agents in Medicinal Chemistry 13(5): 811-29.
209. Chueca, E., et al. (2016). "Proton pump inhibitors display antitumor effects in Barrett's adenocarcinoma cells." Frontiers in Pharmacology 7: 452.
210. Johannessen, T. C., et al. (2019). "Thioridazine inhibits autophagy and sensitizes glioblastoma cells to temozolomide." International Journal of Cancer 144(7): 1735-1745.
211. Israeli, T., et al. (2018). "Opposing effects of intracellular versus extracellular adenine nucleotides on autophagy: implications for β-cell function." Journal of Cell Science 131(15): jcs212969.
212. Min, H., et al. (2014). "Bortezomib induces protective autophagy through AMP-activated protein kinase activation in cultured pancreatic and colorectal cancer cells." Cancer Chemotherapy and Pharmacology 74(1): 167-176.
213. Periyasamy-Thandavan, S., et al. (2010). "Bortezomib blocks the catabolic process of autophagy via a cathepsin-dependent mechanism, affects endoplasmic reticulum stress and induces caspase-dependent cell death in antiestrogen-sensitive and resistant ER+ breast cancer cells." Autophagy 6(1): 19-35.

214. Mackeh, R., et al. (2013). "Autophagy and microtubules - new story, old players." Journal of Cell Science 126(Pt 5): 1071-1080.
215. Yu, Y. F., et al. (2017). "Paclitaxel induces autophagy in gastric cancer BGC823 cells." Ultrastructural Pathology 41(4): 284-290.
216. Hsieh, M. J., et al. (2017). "Transcriptional regulation of Mcl-1 plays an important role of cellular protective effector of vincristine-triggered autophagy in oral cancer cells (vol 19, pg 455, 2015)." Expert Opinion on Therapeutic Targets 21(5): 557-558.
217. Zheng, X. Y., et al. (2015). "Low concentrations of chloroquine and 3-methyladenine suppress the viability of retinoblastoma cells synergistically with vincristine independent of autophagy inhibition." Graefe’s Archive for Clinical and Experimental Ophthalmology 253(12): 2309-2315.
218. Wang, S. H., et al. (2008). "Cadmium-induced autophagy and apoptosis are mediated by a calcium signaling pathway." Cellular and Molecular Life Sciences 65(22): 3640-3652.
219. Li, Q., et al. (2010). "Lithium reduces apoptosis and autophagy after neonatal hypoxia-ischemia." Cell Death & Disease 1: e56
220. Sarkar, S., et al. (2005). "Lithium induces autophagy by inhibiting inositol monophosphatase." Journal of Cell Biology 170(7): 1101-1111.
221. Hu, M., et al. (2017). "Nimodipine activates neuroprotective signaling events and inactivates autophages in the VCID rat hippocampus." Neurological Research 39(10): 904-909.
222. Williams, A., et al. (2008). "Novel targets for Huntington's disease in an mTOR-independent autophagy pathway." Nature Chemical Biology 4(5): 295-305.
223. Zhang, X., et al. (2017). "Trifluoperazine, a novel autophagy inhibitor, increases radiosensitivity in glioblastoma by impairing homologous recombination." Journal of Experimental Clinical Cancer Research 36:118.
224. Zhang, Y., et al. (2017). "Rescue of Pink1 deficiency by stress-dependent activation of autophagy." Cell Chemical Biology 24(4): 471-480.
225. Dupere-Richer, D., et al. (2013). "Vorinostat-induced autophagy switches from a death-prompting to a cytoprotective signal to drive acquired resistance." Cell Death & Disease 4: e486.
226. Stankov, M. V., et al. (2014). "Histone deacetylase inhibitors induce apoptosis in myeloid leukemia by suppressing autophagy." Leukemia 28(3): 577-88.
